# Efficient Heterogeneous Copper-Catalyzed Alder-Ene Reaction of Allenynamides to Pyrrolines

**DOI:** 10.1021/acscatal.1c05147

**Published:** 2022-01-18

**Authors:** Zhiyao Zheng, Luca Deiana, Daniels Posevins, Abdolrahim A. Rafi, Kaiheng Zhang, Magnus J. Johansson, Cheuk-Wai Tai, Armando Córdova, Jan-E. Bäckvall

**Affiliations:** † Department of Organic Chemistry, Arrhenius Laboratory, Stockholm University, SE-10691 Stockholm, Sweden; ‡ Department of Natural Sciences, Holmgatan 10, Mid Sweden University, 85179 Sundsvall, Sweden; § AstraZeneca R&D, Innovative Medicines, Cardiovascular and Metabolic Disorders, Medicinal Chemistry, Pepparedsleden 1, SE-431 83 Mölndal, Sweden; ∥ Department of Materials and Environmental Chemistry, Arrhenius Laboratory, Stockholm University, SE-10691 Stockholm, Sweden

**Keywords:** cellulose, heterogeneous, nanocopper, Alder-ene reaction, pyrrolines

## Abstract

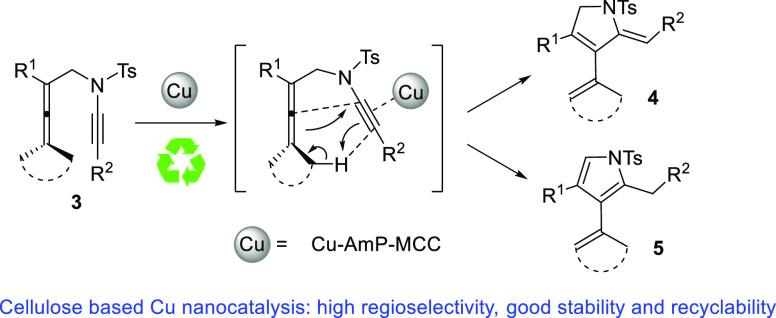

Herein, we describe an efficient nanocopper-catalyzed Alder-ene reaction of allenynamides. The copper nanoparticles were immobilized on amino-functionalized microcrystalline cellulose. A solvent-controlled chemoselectivity of the reaction was observed, leading to the chemodivergent synthesis of pyrrolines (2,5-dihydropyrroles) and pyrroles. The heterogeneous copper catalyst exhibits high efficiency and good recyclability in the Alder-ene reaction, constituting a highly attractive catalytic system from an economical and environmental point of view.

The Alder-ene reaction has been recognized as a powerful synthetic tool for the rapid construction of C–C bonds with high atom economy and efficiency.^1^ Since the seminal work by Trost on the palladium-catalyzed intramolecular ene reactions of 1,6-enynes,^2^ the ene-type cycloisomerizations of various 1,*n*-unsaturated systems, such as dienes,^3^ enynes,^4^ triynes,^5^ and enallenes,^6^ have been reported. However, the corresponding Alder-ene reaction of allenynes is less investigated.^4f,7^ In 2002, Brummond reported the rhodium(I)-catalyzed formal Alder-ene-type reaction of 1,6-allenynes for stereoselective synthesis of cross-conjugated trienes (Scheme 1a).^8^ Malacria, Fensterbank, and Aubert disclosed the platinum-, gold-, and silver-catalyzed cycloisomerizations of 1,6-allenynes to provide the corresponding trienes (Scheme 1a).^9^ Despite the above-mentioned progress, studies on catalytic Alder-ene reactions of 1,*n*-allenynes using green synthetic protocols via nonprecious metal catalysis are still rare and are in high demand.

**Scheme 1:**
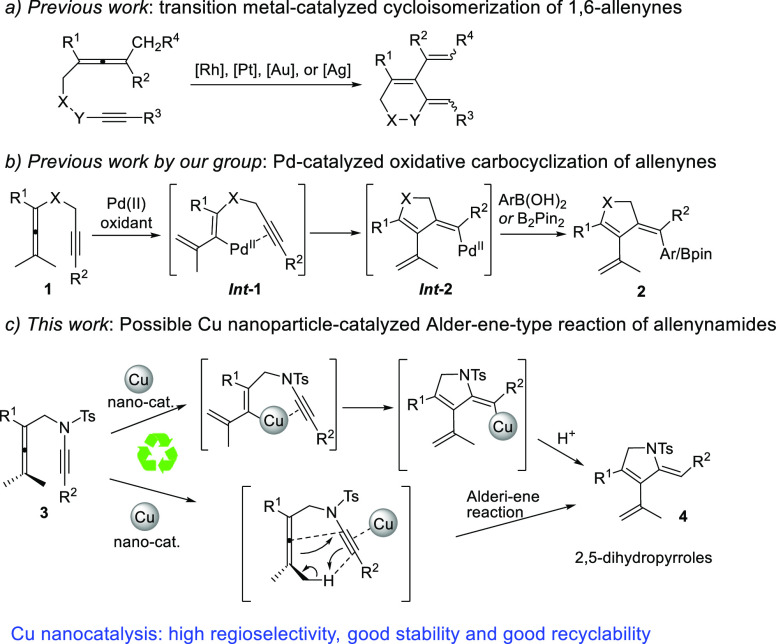
Cycloisomerization and Oxidative Carbocyclization of Allenynes: (a) [Rh], Ref (8); [Pt], [Au], and [Ag], Ref (9). (b) Refs (10f)–^10i^. (c) This Work

Our group has a long-standing involvement in palladium-catalyzed oxidative functionalization of allenes.^10^ In the case of alkyne-assisted palladium-catalyzed oxidative carbocyclization of allenynes,^10f,10i^ the nucleophilic attack on palladium by the allene and the subsequent alkyne insertion lead to the construction of a variety of 5-membered ring compounds (Scheme 1b). However, in our initial attempts to examine the reactivity of allenynamides, an analogous Pd(II)-catalyzed cycloisomerization without the aid of oxidant was observed, leading to the formation of pyrrolines (2,5-dihydropyrroles) and pyrroles.^11^ The latter reaction^11^ may proceed via a similar pathway as in Scheme 1b with generation of a vinylpalladium intermediate such as ***Int*-1**. We envisioned that first-row transition metals, such as copper could also promote such cycloisomerizations leading to Alder-ene-type products.

Transition metal nanoparticles immobilized on heterogeneous materials have shown to be promising catalysts for a wide range of organic transformations with good stability and recyclability.^12^ Cellulose as one of the most abundant organic biopolymers has been recognized as an excellent choice for immobilization of various transition metal catalysts.^12d,12f,13^ Our group has previously employed a heterogeneous amino-functionalized crystalline nanocellulose-based palladium catalyst (Pd-AmP-CNC) in the oxidative carbonylation of allene amide.^12f^ In comparison to homogeneous palladium catalysts such as Pd(OAc)_2_, Pd-AmP-CNC exhibits higher efficiency with good recyclability.^12d^

Given the surging interest in green and sustainable nanocatalysts, we were motivated to investigate the catalytic activity of copper nanoparticles immobilized on microcrystalline cellulose (MCC) in the Alder-ene reaction of allenynamides (Scheme 1c). Commercially available MCC as Avicel PH-101 has a very low price^14^ and can serve as a sustainable support in heterogeneous catalysis.^15^ We postulated that this reaction can occur through a dienyl copper intermediate (Scheme 1c, upper part) in analogy with the palladium-catalyzed reaction shown in Scheme 1b, or via a copper-catalyzed concerted Alder-ene reaction (Scheme 1c, lower part). Herein, we report on a nanocopper-catalyzed Alder-ene reaction of allenynamides **3** to pyrrolines **4** using mixed Cu(I/II) nanoparticles immobilized on aminopropyl-functionalized MCC (Cu-AmP-MCC). The easy handling of this simple catalyst and its efficient recycling (6 cycles with maintained high activity demonstrated) makes this novel catalytic Alder-ene reaction highly practical.

A schematic overview of the synthesis of the Cu-AmP-MCC nanocatalyst is outlined in Scheme 2. Amino-functionalized MCC (AmP-MCC), which had been prepared by organocatalytic silylation,^13e^ was subjected to an aqueous solution of Cu(OTf)_2_ (pH 9) at room temperature for 24 h to furnish a Cu(II)-precatalyst. This precatalyst was subsequently reduced by NaBH_4_ in H_2_O at ambient temperature to generate the mixed valence Cu(I/II) Cu-AmP-MCC nanocatalyst.^16^ To obtain information regarding the oxidation state of the copper nanoparticles, the catalyst was analyzed by XPS, and it provided evidence for the presence of both Cu(I) and Cu(II) in an estimated ratio of 1.1:1, respectively.^17^ The copper nanocatalyst was characterized by scanning transmission emission microscopy (STEM) to determine the size of the supported nanoparticles. According to the STEM, the nanoparticles of the Cu-AmP-MCC catalyst are well-dispersed and have an average particle size of 3–8 nm (Figure 1).

**Scheme 2:**
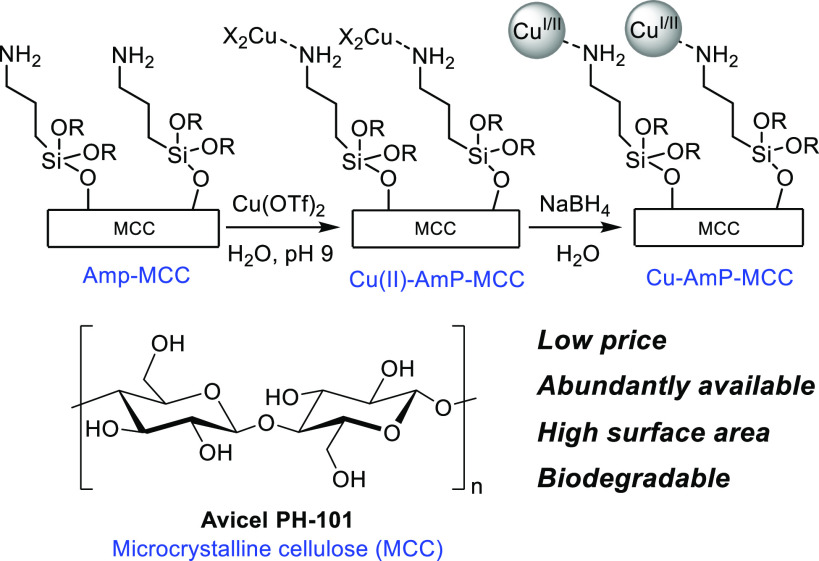
General Procedure for the Synthesis of Cu-AmP-MCC Catalyst

**Figure 1:**
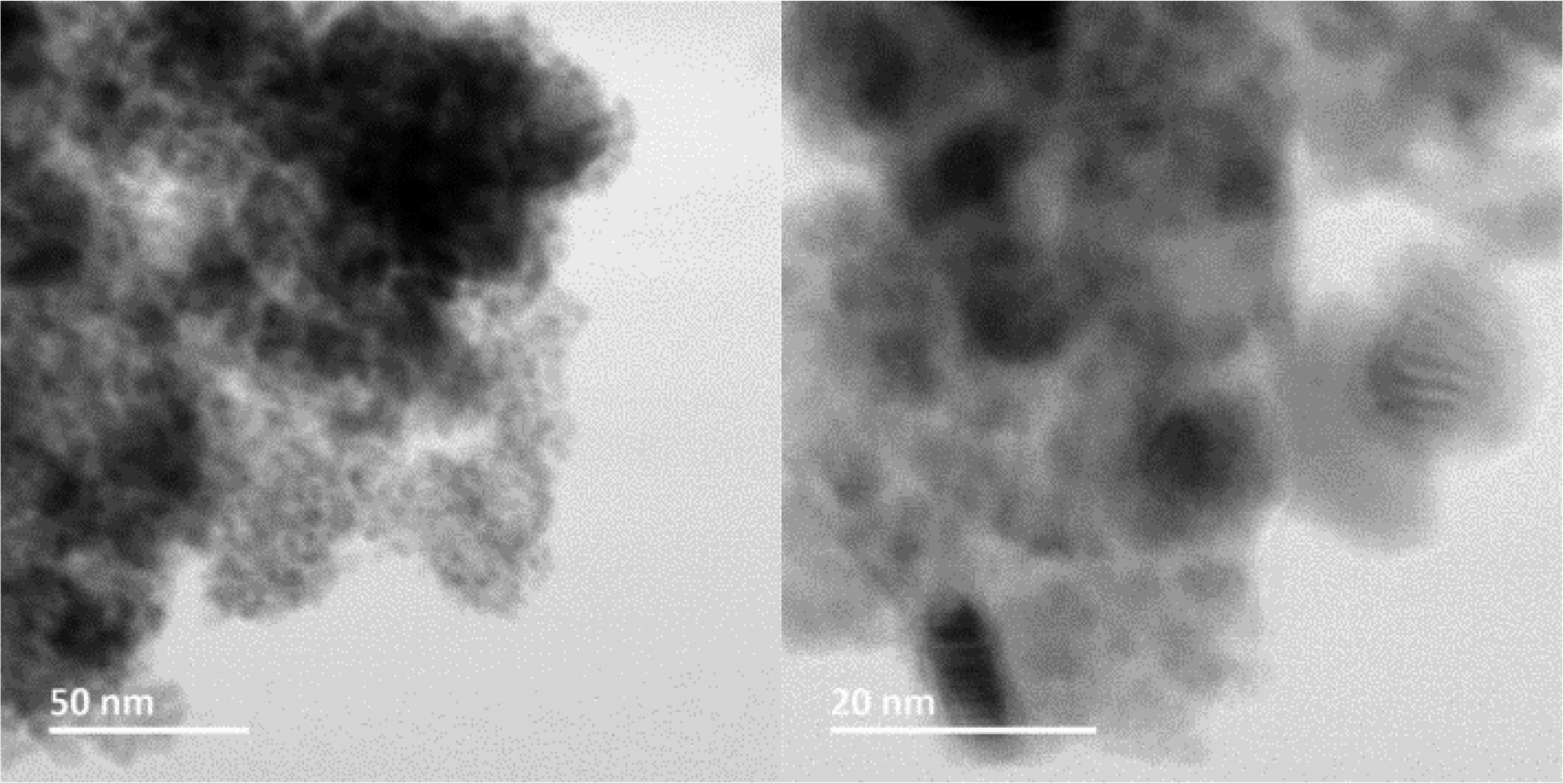
STEM bright-field images of Cu-AmP-MCC catalyst, (a) with 50 nm scale bar and (b) with 20 nm scale bar. Moiré fringes given by overlapping of crystalline particles are observed.

To test our hypothesis, we initially investigated the reaction of allenynamide **3a** bearing a *n*-pentylated ynamide and a trisubstituted allene by using Cs_2_CO_3_ (2.0 equiv) as the base and Cu-AmP-MCC as the catalyst (5.4 mol %). To our delight, the cyclization reaction proceeded smoothly to give the Alder-ene product 2,5-dihydropyrrole **4a** in high selectivity in 65% NMR yield within 24 h at 60 °C in toluene (Table 1, entry 1). In the absence of Cs_2_CO_3_, a 61% yield of **4a** was observed together with trace amounts of the pyrrole product **5a**, which was probably generated from the isomerization of **4a**. Switching the catalyst to homogeneous copper salt Cu(OTf)_2_ afforded **4a** in a similar yield (entry 2). Other transition metal π-acids such as AgOTf and Sc(OTf)_3_ were also effective for this transformation, leading to **4a** in 48% and 53% yield, respectively (entries 3 and 4). These results indicate that the heterogeneous Cu catalyst displayed comparable or even superior reactivity for this reaction, compared with that of the homogeneous catalysts. A control experiment in the absence of catalyst gave 9% yield of **4a** (entry 5). Solvent screening showed that significantly lower yields were observed in the nanocopper-catalyzed reaction when it was carried out in THF, MeOH, or CH_3_CN (18–36% yields entries 6–8). Interestingly, by using chlorinated solvents, such as DCE and CHCl_3_, we observed the formation of the pyrrole product **5a** in 19% and 35% yield, respectively, (entries 9 and 10). These results demonstrate that solvent plays an important role in controlling the chemoselectivity of this cyclization reaction. The relatively acidic solvent CHCl_3_ favors the isomerization of **4a** to **5a**, which is in accordance with previous observations.^18^ As the substrate **3a** was partially recovered at 60 °C, attempts were made to improve the conversion of **3a** by increasing the reaction temperature. At 80 °C, **4a** was obtained as the exclusive product in 88% isolated yield by using toluene as the solvent (entry 11), while in CHCl_3_, **5a** was obtained in 68% isolated yield as the predominant product (entry 12). A control experiment in the absence of catalyst showed that at 80 °C the thermal Alder-ene reaction afforded only 29% NMR yield of **4a** (entry 13).^19^

**Table 1:**
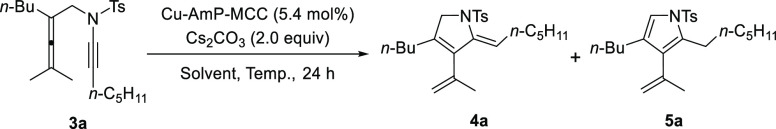
Optimization of Reaction Conditions for the Nanocopper-Catalyzed Reaction of **3a**^a^

entry	catalyst	solvent	*T* (°C)	yield of **4a** (%)^b^	yield of **5a** (%)^b^
1	Cu-AmP-MCC	toluene	60	65 (61)^c^	0 (<5)^c^
2^d^	Cu(OTf)_2_	toluene	60	63	0
3^d^	AgOTf	toluene	60	48	0
4^d^	Sc(OTf)_3_	toluene	60	53	0
5	-	toluene	60	9	0
6	Cu-AmP-MCC	THF	60	36	0
7	Cu-AmP-MCC	MeOH	60	21	0
8	Cu-AmP-MCC	CH_3_CN	60	18	0
9^c^	Cu-AmP-MCC	DCE	60	28	19
10^c^	Cu-AmP-MCC	CHCl_3_	60	18	35
11	Cu-AmP-MCC	toluene	80	91 (88)^e^	0
12^c^	Cu-AmP-MCC	CHCl_3_	80	3	71 (68)^e^
13	-	toluene	80	29	0

a The reaction was carried out in the indicated solvent (1 mL) using **3a** (0.1 mmol) and Cs_2_CO_3_ (0.2 mmol) in the presence of copper nanocatalyst (5.4 mol %).

b Determined by NMR using 1,1,2,2-tetrachloroethane as the standard.

c Without Cs_2_CO_3_.

d 5.0 mol % metal catalyst was used.

e Isolated yield.

With the optimized reaction conditions in hand, we focused our attention on the scope of the reaction as well as the divergent synthesis of **4** and **5** (Scheme 3). By using toluene as solvent, allenynamide **3** with phenylethyl, phenyl, and methyl groups in the R^1^ position worked equally well, furnishing **4b**, **4c**, and **4d** in 85%, 90%, and 81% yield, respectively. The presence of an ester substituent resulted in a lower yield of the desired product **4e** (48%), possibly due to undesired side reactions caused by the ethoxycarbonylmethyl group in the presence of base. Also, a substrate where the two methyl substituents on the allene had been replaced by a cyclopentylidene group worked well, affording the corresponding product **4f** in good yield (78%). Substrates bearing aryl, cyclohexyl, trimethylsilyl, and (*tert*-butyldimethylsilyloxy)methyl groups in the R^2^ position of **3** were well tolerated in the reaction, furnishing **4g**–**4k** in 70–82% yields. The (*Z*)-configuration of the exocyclic double bond in 2,5-dihydropyrroles **4** was established by comparison of the NMR spectra with those of the known products previously reported.^18^ By using CHCl_3_ as solvent, the cascade cycloisomerization-isomerization reaction of various allenynamide substrates worked well to give pyrroles **5**. In this way, pyrrole products **5a**–**5g** and **5j** were prepared in good yields (41–76%).

**Scheme 3:**
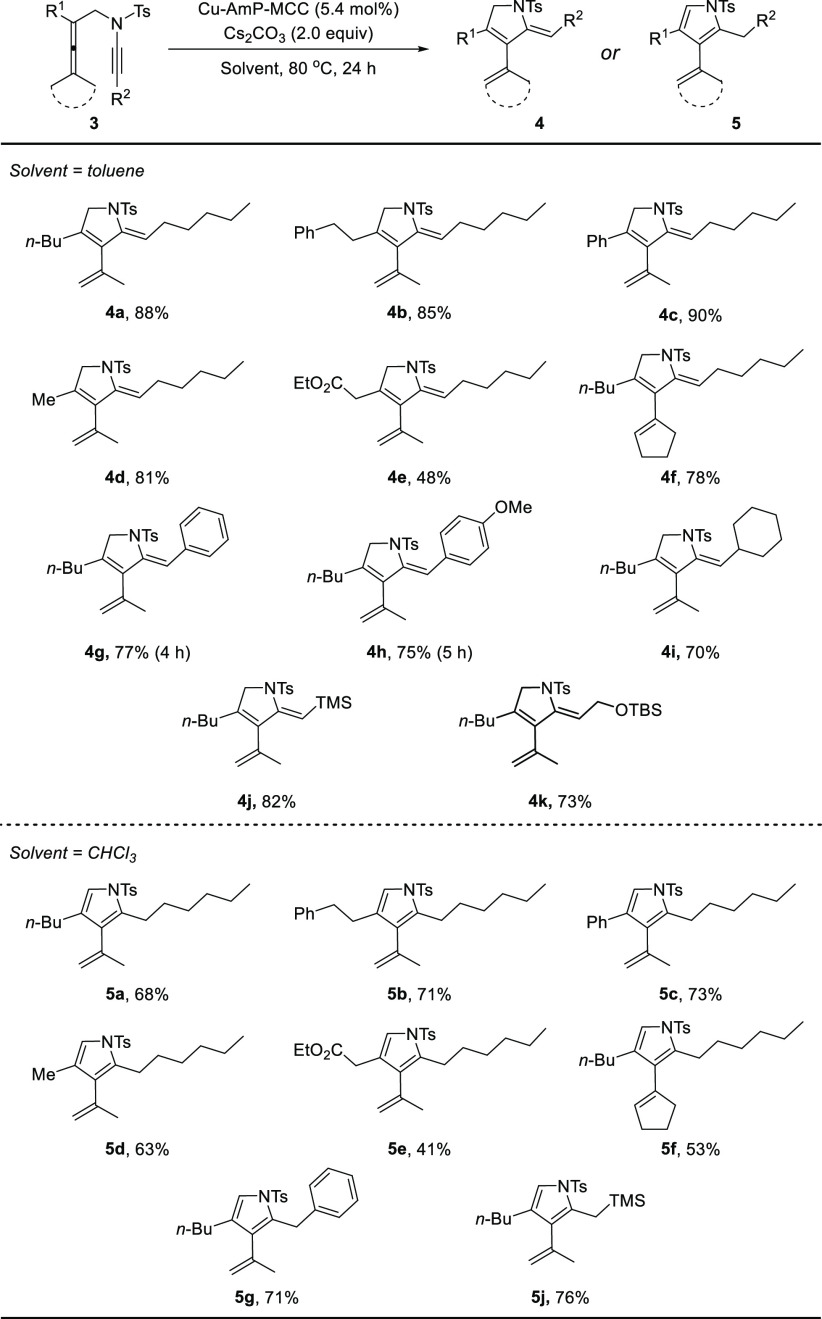
Regioselective and Divergent Synthesis of **4** and **5** from Allenynamides **3** Reaction conditions: **3** (0.20 mmol), Cu-AmP-MCC (5.4 mol %), Cs_2_CO_3_ (0.40 mmol), toluene (2.0 mL) or CHCl_3_ (2.0 mL), 80 °C, 24 h.

To further evaluate the efficiency of the heterogeneous nanocopper catalyst in the reaction of **3** to Alder-ene products **4**, we conducted catalyst recycling experiments using **3a** as substrate. The recycling experiments revealed that the high efficiency of the catalyst could be maintained from the first to the sixth run (Figure 2).^20^ Inductively coupled plasma optical emission spectroscopy (ICP-OES) analysis showed that the copper concentration in the recovered solution from the first run was <1 ppm, which shows that leaching is neglectable.

**Figure 2:**
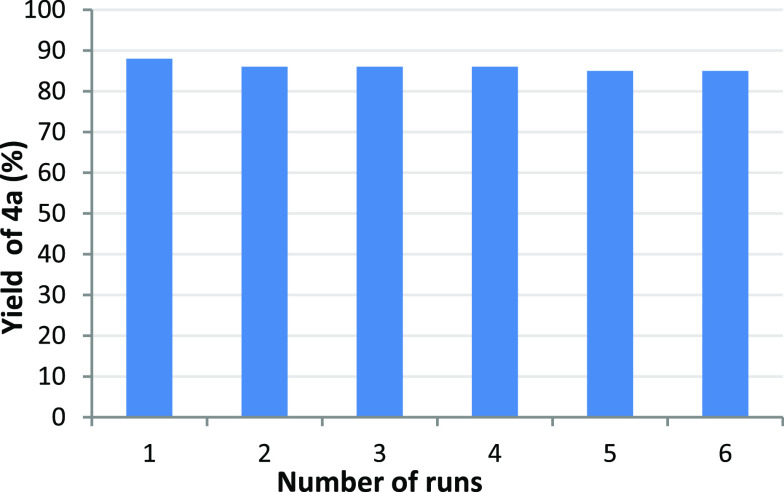
Recycling experiments of Cu-AmP-MCC-catalyzed reaction of **3a** to Alder-ene product **4a**

To gain insight into the mechanism of this reaction, deuterium-labeling experiments were conducted (Scheme 4). When D_2_O (2 equiv) was added to the reaction, no deuterium incorporation of the corresponding product **4a** was observed (Scheme 4a), which excludes the possibility that the H atom in the newly formed alkene comes from protonation of a vinylcopper intermediate (cf. upper part of Scheme 1c). Moreover, when deuterated substrate *d*^*6*^-**3a** was subjected to the reaction in the presence of H_2_O (2 equiv), *d*^*6*^-**4a** was obtained as the single product (65% yield) with complete D atom transfer (100%) from the terminal methyl group in *d*^*6*^-**3a** to the alkenyl position in *d*^*6*^-**4a** (Scheme 4b). These results rule out the mechanism via a dienylcopper intermediate (upper part of Scheme 1c) and provide strong support for a concerted Alder-ene reaction (lower part of Scheme 1c).

**Scheme 4:**
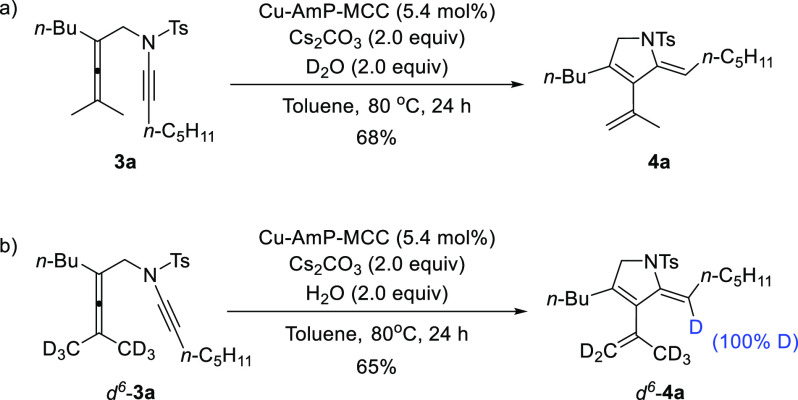
Deuterium-Labeling Experiments: (a) D_2_O as Additive and (b) Deuterated Substrate with H_2_O as Additive

Based on the observed stereochemical outcome and the deuterium-labeling experiments, a possible mechanism for the reaction is proposed in Scheme 5. The strong electrophilic activation of the alkyne in the ynamide moiety by coordination to copper would trigger the cyclization process in which the allenic double bond acts as the “ene” and generates a new carbon–carbon bond with the enophile (activated ynamide) with synchronous allenic H atom migration. The coordination of copper to the polarized ynamide triple bond (***Int*-3** and ***Int*-4**) is essential for lowering the activation barrier of the ene-type cycloisomerization to give 2,5-dihydropyrrole **4**. Although a rhodium-directed metallacycle pathway was proposed in Brummond’s work^8^ and a pathway via external allene attack on an alkyne-metal complex (metal = Au, Ag) was suggested in the work by Malacria, Fensterbank, and Aubert,^9b,9d^ the likely pathway for the nanocopper-catalyzed carbocyclization of **3** to **4** described in the present work involves a concerted Alder-ene reaction. Afterward, **4** can undergo further isomerization to afford pyrrole **5**. The formation of the (*Z*)-exocyclic double bond of **4** together with the outcome of the deuterium experiments to give *d*^*6*^-**4a** is in accordance with a concerted Alder-ene reaction proceeding via π-acid catalysis.^21^

**Scheme 5:**
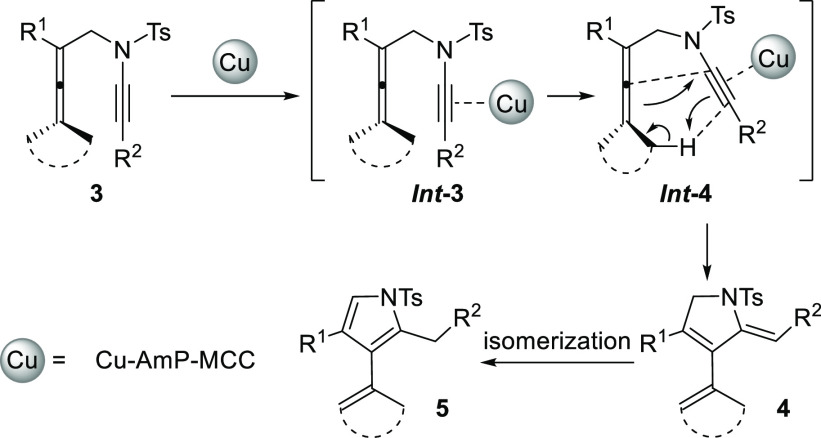
Proposed Mechanism for the Copper-Catalyzed Alder-Ene Reaction of Allenynamides **3**

In conclusion, we have reported an efficient nanocopper-catalyzed Alder-ene reaction of allenynamide for the chemodivergent synthesis of 2,5-dihydropyrroles and pyrroles in which nanocopper particles are immobilized on microcrystalline cellulose (Cu-AmP-MCC). Experimental data support a concerted Alder-ene reaction. The comparative studies of various catalysts showcased the good catalytic performance of Cu-AmP-MCC, with an efficiency similar or superior to other homogeneous metal catalysts. The Cu-AmP-MCC displayed excellent recyclability that enabled it to be used at least six times without any significant loss in activity. Further studies on the mechanism of this reaction as well as the use of the heterogeneous Cu-AmP-MCC catalyst for other ynamide transformations are currently underway in our laboratory along with studies on other Cu-catalyzed transformations that would benefit from the heterogeneous nature of this catalyst.

## Supporting Information Available

Detailed experimental procedures; STEM images; XPS spectra; compound characterization data and NMR spectra (PDF)

## References

[ref1] aAlder K.; Pascher F.; Schmitz A. Über die Anlagerung von Maleinsäure-anhydrid und Azodicarbonsäure-ester an einfach ungesättigte Koh an einfach ungesättigte Kohlenwasserstoffe. Zur Kenntnis von Substitutionsvorgängen in der Allyl-Stellung. Berichte der deutschen chemischen Gesellschaft (A and B Series) 1943, 76, 27–53. 10.1002/cber.19430760105.

[ref2] Trost B. M.; Lautens M. Cyclization via isomerization: a palladium(2+)-catalyzed carbocyclization of 1,6-enynes to 1,3- and 1,4-dienes. J. Am. Chem. Soc. 1985, 107, 1781–1783. 10.1021/ja00292a065.

[ref3] aNarasaka K.; Hayashi Y.; Shimada S.; Yamada J. Asymmetric Intramolecular Ene Reaction Catalyzed by a Chiral Titanium Reagent and Synthesis of (—)-δ-Cadinene. Isr. J. Chem. 1991, 31, 261–271. 10.1002/ijch.199100030.

[ref4] aSturla S. J.; Kablaoui N. M.; Buchwald S. L. A Titanocene-Catalyzed Intramolecular Ene Reaction: Cycloisomerization of Enynes and Dienynes. J. Am. Chem. Soc. 1999, 121, 1976–1977. 10.1021/ja9839567.

[ref5] aCho E. J.; Lee D. Selectivity in the Ruthenium-Catalyzed Alder Ene Reactions of Di- and Triynes. J. Am. Chem. Soc. 2007, 129, 6692–6693. 10.1021/ja0719430. 17488017 PMC2518692

[ref6] aNärhi K.; Franzén J.; Bäckvall J.-E. An Unexpectedly Mild Thermal Alder-Ene-Type Cyclization of Enallenes. J. Org. Chem. 2006, 71, 2914–2917. 10.1021/jo060013g. 16555856

[ref7] aTrost B. M.; Pinkerton A. B. A Ruthenium-Catalyzed Two-Component Addition To Form 1,3-Dienes. J. Am. Chem. Soc. 1999, 121, 4068–4069. 10.1021/ja990291f. 11741409

[ref8] Brummond K. M.; Chen H.; Sill P.; You L. A Rhodium(I)-Catalyzed Formal Allenic Alder Ene Reaction for the Rapid and Stereoselective Assembly of Cross-Conjugated Trienes. J. Am. Chem. Soc. 2002, 124, 15186–15187. 10.1021/ja027588p. 12487589

[ref9] aCadran N.; Cariou K.; Hervé G.; Aubert C.; Fensterbank L.; Malacria M.; Marco-Contelles J. PtCl_2_-Catalyzed Cycloisomerizations of Allenynes. J. Am. Chem. Soc. 2004, 126, 3408–3409. 10.1021/ja031892g. 15025452

[ref10] aPersson A. K. Å.; Jiang T.; Johnson M. T.; Bäckvall J.-E. Palladium-Catalyzed Oxidative Borylative Carbocyclization of Enallenes. Angew. Chem., Int. Ed. 2011, 50, 6155–6159. 10.1002/anie.201008032. 21591031

[ref11] The cycloisomerization of **3a** (0.2 mmol) took place in the presence of Pd(OAc)_2_ in toluene at 60 °C to give **4a** in 23% yield and **5a** in 9% yield. 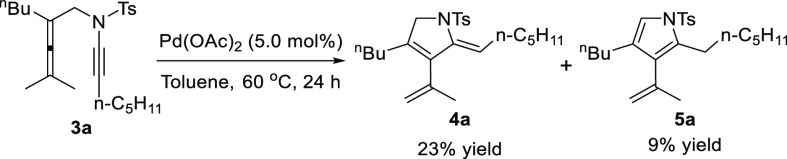

[ref12] aChng L. L.; Erathodiyil N.; Ying J. Y. Nanostructured Catalysts for Organic Transformations. Acc. Chem. Res. 2013, 46, 1825–1837. 10.1021/ar300197s. 23350747

[ref13] aShaghaleh H.; Xu X.; Wang S. Current progress in production of biopolymeric materials based on cellulose, cellulose nanofibers, and cellulose derivatives. RSC Adv. 2018, 8, 825–842. 10.1039/C7RA11157F. PMC9076966 35538958

[ref14] Avicel Ph 101 can be obtained for 2.00 US$-3.50 US$/ kilogram (20 kilograms min order) and for laboratory use (Avicel PH-101, Merck) for 137.00 US$/kilogram.

[ref15] Deiana L.; Rafi A. A.; Naidu V. R.; Tai C.-W.; Bäckvall J.-E.; Córdova A. Artificial plant cell walls as multi-catalyst systems for enzymatic cooperative asymmetric catalysis in non-aqueous media. Chem. Commun. 2021, 57, 8814–8817. 10.1039/D1CC02878B. 34382975

[ref16] Rafi A. A.; Ibrahem I.; Córdova A. Copper nanoparticles on controlled pore glass (CPG) as highly efficient heterogeneous catalysts for “click reactions. Sci. Rep. 2020, 10, 20547 10.1038/s41598-020-77629-3. 33239720 PMC7688963

[ref17] See Supporting Information.

[ref18] Li M.-B.; Grape E. S.; Bäckvall J.-E. Palladium-Catalyzed Stereospecific Oxidative Cascade Reaction of Allenes for the Construction of Pyrrole Rings: Control of Reactivity and Selectivity. ACS Catal. 2019, 9, 5184–5190. 10.1021/acscatal.9b01041.

[ref19] Zriba R.; Gandon V.; Aubert C.; Fensterbank L.; Malacria M. Alkyne versus Allene Activation in Platinum- and Gold-Catalyzed Cycloisomerization of Hydroxylated 1,5-Allenynes. Chem.—Eur. J. 2008, 14, 1482–1491. 10.1002/chem.200701522. 18034446

[ref20] Scott S. L. A Matter of Life(time) and Death. ACS Catal. 2018, 8, 8597–8599. 10.1021/acscatal.8b03199.

[ref21] Saito Y.; Kobayashi S. Chiral Heterogeneous Scandium Lewis Acid Catalysts for Continuous-Flow Enantioselective Friedel-Crafts Carbon-Carbon Bond-Forming Reactions. Angew. Chem., Int. Ed. 2021, 60, 26566 10.1002/anie.202112797. 34661969

