# Supplementary material for: Efficient Heterogeneous Copper-Catalyzed Alder-Ene Reaction of Allenynamides to Pyrrolines
Source: ACS Catal. 2022 Jan 18;12(3):1791–6. doi: 10.1021/acscatal.1c05147 (PMC8822631; doi:10.1021/acscatal.1c05147)

Supporting Information

Efficient Heterogeneous Copper-Catalyzed Alder-Ene Reaction of Allenynamides to Pyrrolines

Zhiyao Zheng,[a] Luca Deiana,[b] Daniels Posevins,[a] Abdolrahim A. Rafi,[b] Kaiheng Zhang,[b] Magnus J. Johansson,[c] Cheuk-Wai Tai,[d] Armando Córdova,*[b] and Jan-E. Bäckvall*[ab]

[a] Department of Organic Chemistry, Arrhenius Laboratory, Stockholm University, SE-10691 Stockholm, Sweden

[b] Department of Natural Sciences, Holmgatan 10, Mid Sweden University, SE-85179, Sundsvall, Sweden

[c] AstraZeneca R&D, Innovative Medicines, Cardiovascular and Metabolic Disorders, Medicinal Chemistry, Pepparedsleden 1, SE-431 83 Mölndal, Sweden

[d] Department of Materials and Environmental Chemistry, Arrhenius Laboratory, Stockholm University, SE-10691 Stockholm, Sweden

E-mail: jeb@organ.su.se; armando.cordova@miun.se

Table of Contents

General information S2

Preparation of Cu-AmP-MCC S3

Characterizations of Cu-AmP-MCC by STEM S4

Characterizations of Cu-AmP-MCC by XPS S5

General procedure for the preparation of allenynamide **3** S7

Optimization of reaction conditions S13

General procedure for the Cu-AmP-MCC-catalyzed Alder-ene reaction of **3** to 2,5-dihydropyrroles **4** S14

General procedure for the Cu-AmP-MCC-catalyzed Alder-ene reaction of **3** to pyrroles **5** S20

Deuterium-labeling experiments S24

Recycling experiments and leaching test S25

References S26

NMR spectra S27

General information

Unless otherwise noted, all reagents were used as received from commercial suppliers. Reactions were monitored using thin-layer chromatography (SiO_2). TLC plates were visualized with UV light (254 nm) or $KMnO_4$ stain. Flash chromatography was carried out with 60Å (particle size 35-70 μm) normal flash silica gel. NMR spectra were recorded on a Bruker 400 or 500 MHz spectrometer unless otherwise stated. Chemical shifts (δ) are reported in ppm, using the residual solvent peak in $CDCl_3$ (^1H = 7.26 ppm and ^{13}C = 77.0 ppm) as internal standard, and coupling constants (*J*) are given in Hz. HRMS were recorded on a Bruker micrOTOF instrument using ESI technique. Scanning transmission electron microscopy (STEM) was carried out using a 200 kV JEOL 2100F microscope, which equips with a Schottky field-emission gun and ultrahigh-resolution pole-piece (Cs=0.5 mm). Bright-field (BF) and High angle annular dark field (HAADF) were acquired simultaneously using Gatan BF and JEOL ADF detectors, respectively, through Gatan DigitalMicrograph. The samples in vials were shaken, in order to have small pieces. Then the samples were dispersed onto Cu TEM supporting grid with holey carbon films without using solvent. X-ray photoelectron spectroscopy (XPS) was used to determine the oxidation state of the Cu nanoparticles. All XPS data were processed using Kratos software. XPS spectral data were collected using a Kratos Axis Ultra DLD electron spectrometer with a monochromatized Al-Kα source operating at 120W. The binding energies (BE) were scaled with regards to the aliphatic C 1s carbon set to 285.0 eV. The concentration of copper was determined with inductively coupled plasmaoptical emission spectrometry (ICP-OES) by Medac Ltd. (UK) on Varian Vista MPZ.

Preparation of Cu-AmP-MCC

Preparation of amino-functionalized microcrystalline cellulose (AmP-MCC):[1] In an oven dried flask, MCC (1.0 g), tartaric acid (97.6 mg, 5 mol% to silane) was dispersed in dry toluene (20 mL). Next, 3-aminopropyltrimethoxysilane (2.27 mL, 13.0 mmol) was added and the mixture was stirred at 82 °C for 48 h. The suspension was then centrifuged and the crude AmP-MCC was washed using soxholated extraction with acetone. After 16h, the resulting AmP-MCC was dried under vacuum for 24 h.

Preparation of the mixed valence Cu(I/II) nanocatalyst Cu-AmP-MCC:[2] To a suspension of AmP-MCC (1.0 g) in pH-adjusted H_2O solution (25 ml, pH 9) by the use of 0.1 N LiOH, was added a suspension of copper(II) trifluoromethanesulfonate ($Cu(OTf)_2$, 0.3 g) in deionized water (20 mL, pH 9) at room temperature. After stirring for 24 h, the formed Cu(II)-AmP-MCC with pale-blue color was recovered by centrifugation and was washed with deionized H_2O (3 × 30 mL) and acetone (3 × 30 mL) by using centrifuge. The washed Cu(II)-AmP-MCC was collected by decantation and dried overnight under vacuum.

In the next step, the dry Cu(II)-AmP-MCC was suspended in deionized water (35 mL), and $NaBH_4$ (190.0 mg, 5.0 mmol, 3.7 equiv to copper) in deionized water (15 mL) was added slowly at room temperature. After vigorous stirring for 1 h, the resulting mixed valence Cu(I/II) nanocatalyst Cu-AmP-MCC was recovered by centrifugation and was washed with deionized H_2O (3×30 mL) and acetone (3×30 mL) by the use of a centrifuge. The washed Cu-AmP-MCC was collected by decantation, dried for 48 h under vacuum and obtained as dark blue amorphous powder. The Cu-AmP-MCC was characterized by STEM and XPS (see Figures S1 and S2).

Characterizations of Cu-AmP-MCC by STEM

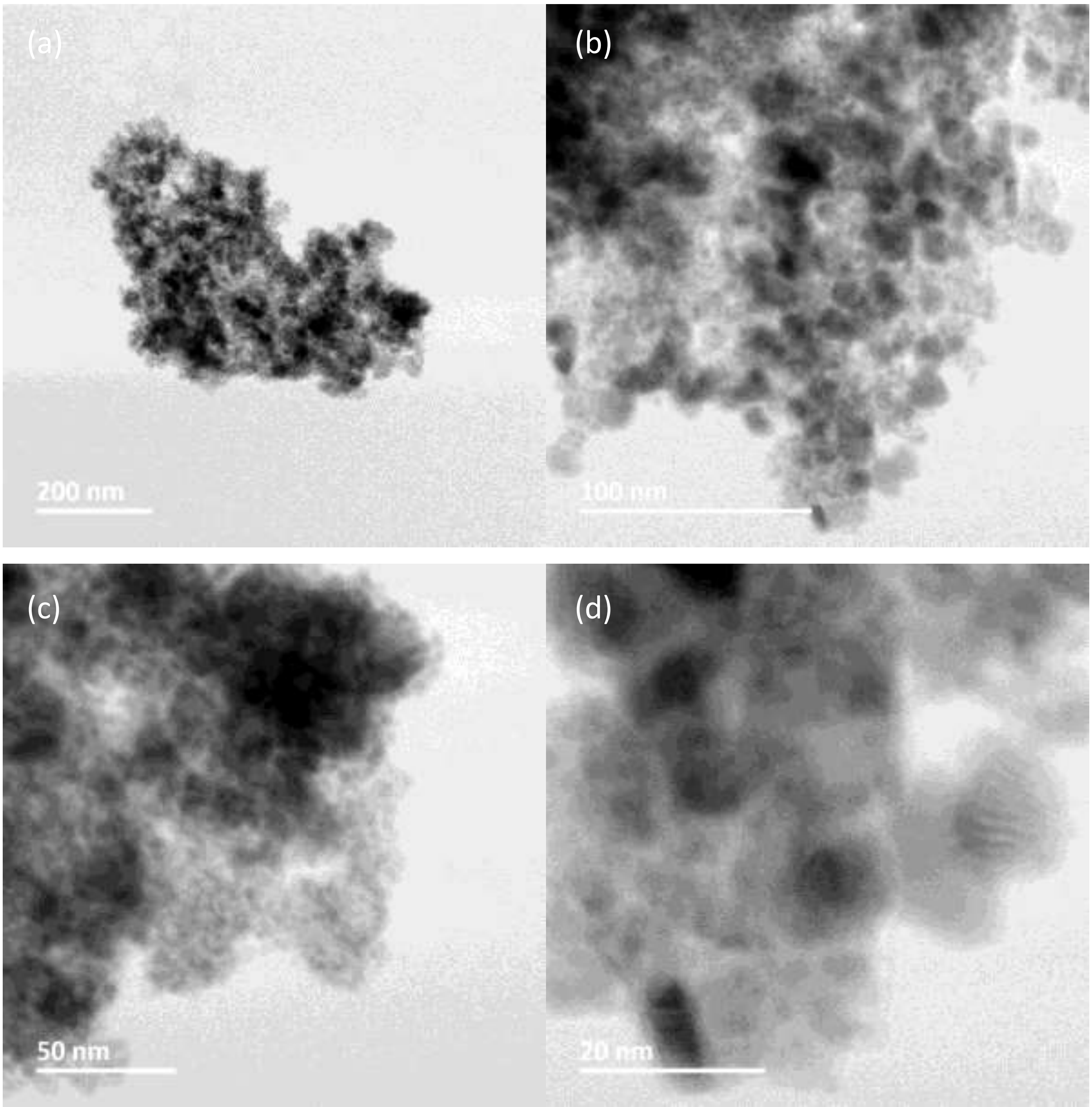

Figure S1. STEM bright-field images of Cu-AmP-MCC catalyst, a) with 200 nm scale bar and b) with 100 nm scale bar, c) with 50 nm scale bar and d) with 20 nm scale bar. Moiré fringes given by overlapping of crystalline particles are observed.

Characterizations of Cu-AmP-MCC by XPS

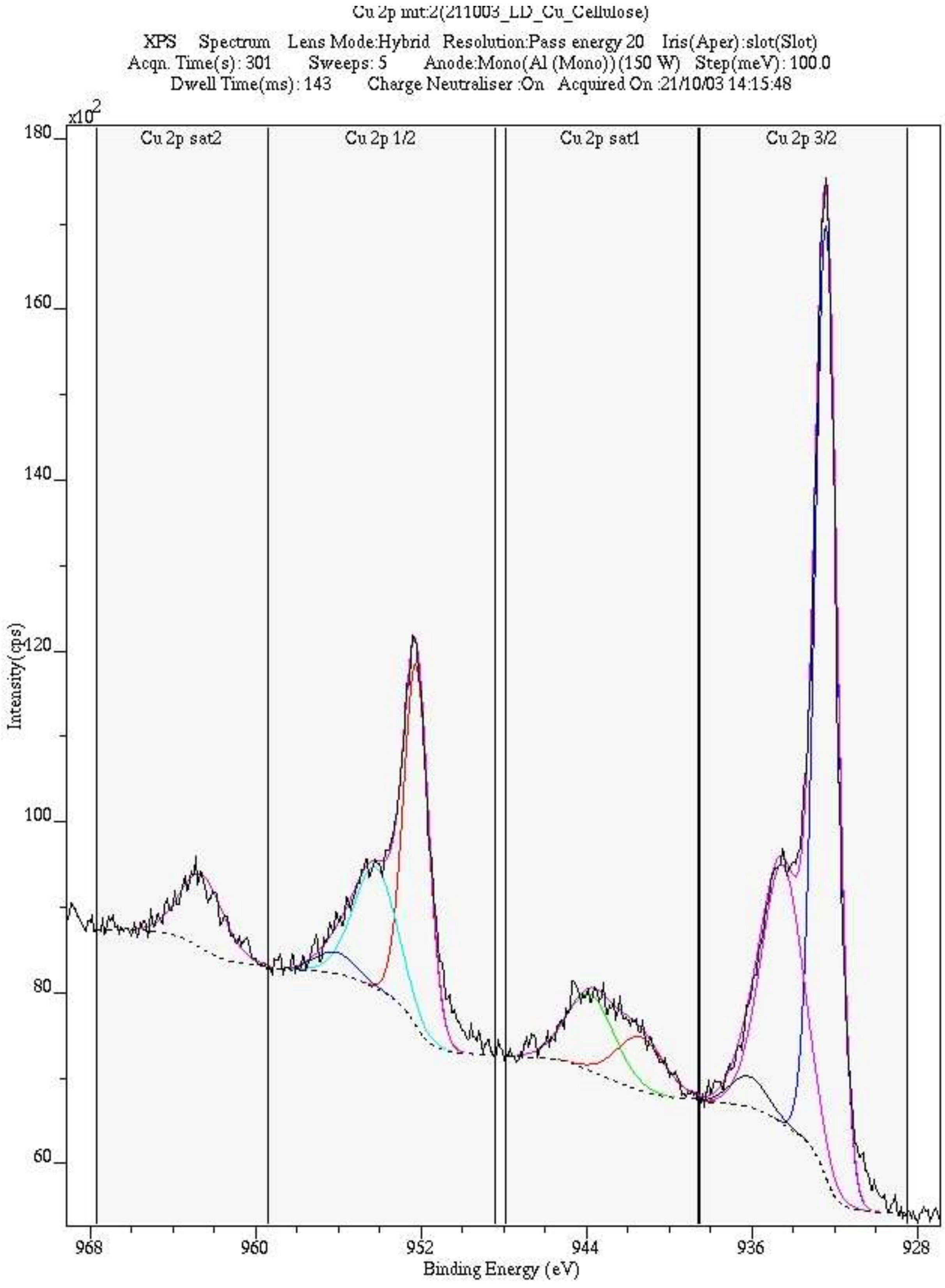

Figure S2. XPS spectrum of the Cu2p state of Cu-AmP-MCC nanocatalyst

Table S1. Cu 2p spectrum components of Cu-AmP-MCC nanocatalyst

BE, eV	FWHM, eV	AC, at%	
932.5	1.25	4.05	Cu 2p 3/2 Cu(I)
952.3	1.4	1.66	Cu 2p 1/2 Cu(I)
		∑ = 5.71	
934.6	2.4	2.1	Cu 2p 3/2 Cu(II)
954.2	2.45	0.98	Cu 2p 1/2 Cu(II)
936.2	2.1	0.21	Cu 3/2 Cu(II) "tail"
956.2	2.25	0.16	Cu 1/2 Cu(II) "tail"
941.4	2.7	0.48	Cu 2p 3/2 Cu(II) satellite
943.9	2.7	0.68	Cu 2p 3/2 Cu(II) satellite
962.6	2.45	0.59	Cu 2p 1/2 Cu(II) satellite
		∑ = 5.2	

The high resolution spectra for the Cu2p region revealed two main peaks located at 932.5 eV and 952.3 eV belonging to Cu(I) as well as peaks at 934.6 eV and 954.2 eV that are characteristic of Cu(II) (Figure S2). Moreover, a collection of satellite features of these peaks are clearly observed at 936.2 eV, 941.4 eV, 943.9 eV, 956.2 eV and 962.6 eV, which also indicated the presence of Cu(II) species. The atomic ratio between Cu(I) and Cu(II) can be calculated from their atomic concentrations based on the ratio of the combined integrals of the peaks belonging to Cu(I) to those of the peaks belonging to Cu(II). Thus Cu(I) : Cu(II) = 5.71 : 5.2 = 1.1 : 1 (Table S1).

General procedure for the preparation of allenynamide 3

*The allenic sulfonamides **S1** were prepared according to a procedure described in the literature.*[3]

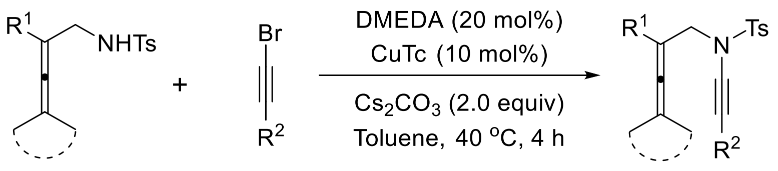

To a flame-dried 25 mL Schlenk flask were added (in the following order) allenic sulfonamide **S1** (2.0 mmol, 1.0 equiv), copper(I) thiophene-2-carboxylate (CuTc) (38.1 mg, 0.20 mmol, 10 mol%) and Cs_2CO_3 (1.30 g, 4.0 mmol) under argon atmosphere. The flask was evacuated under vacuum and flushed with argon for three times. To this mixture toluene (10.0 mL) was added, followed by DMEDA (43.1 µL, 0.40 mmol, 20 mol%) and the alkynyl bromide **S2** (2.4 mmol, 1.2 equiv). The reaction mixture was stirred under argon at 40 °C. After 4 h, the reaction mixture was cooled to rt, filtered through a pad of celite, concentrated in vacuo and purified by flash silica gel column chromatrography (petroleum ether/EtOAc with 3% Et_3N as eluent) to give allenynamide **3**.

N-(hept-1-yn-1-yl)-4-methyl-N-(2-(2-methylprop-1-en-1-ylidene)hexyl)benzenesulfonamide (**3a**)

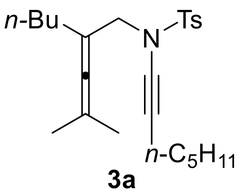

3a

85% isolated yield, colorless oil. ^{1}H NMR (400 MHz, $CDCl_3$) δ ppm 7.77 (d, J = 8.3 Hz, 2H), 7.31 (d, J = 7.9 Hz, 2H), 3.78 (s, 2H), 2.44 (s, 3H), 2.24 (t, J = 7.0 Hz, 2H), 1.90 (t, J = 6.9 Hz, 2H), 1.63 (s, 2H), 1.49 – 1.42 (m, 2H), 1.37 – 1.28 (m, 8H), 0.88 (t, J = 7.0 Hz, 2H), 0.87 (t, J = 7.2 Hz, 2H); ^{13}C NMR (100 MHz, $CDCl_3$) δ 201.43, 144.03, 134.86, 129.47, 127.65, 96.91, 96.65, 73.04, 70.21, 55.13, 30.94, 29.59, 29.37, 28.71, 22.20 (2C), 21.58, 20.55, 18.49, 13.96, 13.95; HRMS (ESI): calc. for $C_{24}H_{35}NNaO_2S$ $[M+Na]^+$ 424.2281; found: 424.2283.

N-(hept-1-yn-1-yl)-4-methyl-N-(2-(2-(methyl-d_3)prop-1-en-1-ylidene-3,3,3-d_3)hexyl)benzene-sulfonamide (d^6-**3a**)

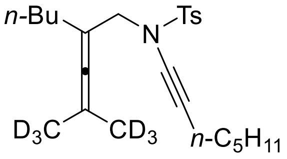

*d*6-**3a**

83% isolated yield, colorless oil. ^{1}H NMR (500 MHz, $CDCl_3$) δ ppm 7.77 (d, *J* = 8.3 Hz, 2H), 7.31 (d, *J* = 8.0 Hz, 2H), 3.77 (s, 2H), 2,43 (s, 3H), 2.23 (t, *J* = 7.1 Hz, 2H), 1.90 (t, *J* = 6.9 Hz, 2H), 1.49 – 1.43 (m, 2H), 1.37 – 1.26 (m, 8H), 0.88 (t, *J* = 7.1 Hz, 2H), 0.87 (t, *J* = 7.2 Hz, 2H); ^{13}C NMR (126 MHz, $CDCl_3$) δ 201.42, 144.02, 134.82, 129.45, 127.61, 96.57 (2C), 77.25, 77.00, 76.75, 73.02, 70.17, 55.12, 30.90, 29.57, 29.33, 28.67, 22.17 (2C), 21.54, 18.46, 13.93, 13.92; HRMS (ESI): calc. for $C_{24}H_{29}D_6NNaO_2S$ $[M+Na]^+$ 430.2657; found: 430.2654.

N-(hept-1-yn-1-yl)-4-methyl-N-(4-methyl-2-phenethylpenta-2,3-dien-1-yl)benzenesulfonamide (**3b**)

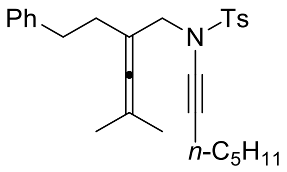

3b

92% isolated yield, colorless oil. ^{1}H NMR (500 MHz, $CDCl_3$) δ ppm δ 7.77 (d, *J* = 8.2 Hz, 2H), 7.30 (d, *J* = 8.3 Hz, 2H), 7.28 – 7.23 (m, 3H), 7.16 (d, *J* = 6.8 Hz, 2H), 3.80 (s, 2H), 2.75 – 2.63 (m, 2H), 2.42 (s, 3H), 2.31 – 2.18 (m, 4H), 1.56 (s, 6H), 1.45 (dt, *J* = 14.2, 7.0 Hz, 2H), 1.36 – 1.27 (m, 4H), 0.88 (t, J = 7.0 Hz, 3H); ^{13}C NMR (126 MHz, $CDCl_3$) δ 201.89, 144.26, 142.06, 134.94, 129.66, 128.55, 128.27, 127.79, 125.74 , 97.80, 96.28, 73.15, 70.47, 55.33, 33.81, 31.17, 28.86, 22.34, 21.75, 20.63, 18.64, 14.14; HRMS (ESI): calc. for $C_{28}H_{35}NNaO_2S$ $[M+Na]^+$ 472.2281; found: 472.2283.

N-(hept-1-yn-1-yl)-4-methyl-N-(4-methyl-2-phenylpenta-2,3-dien-1-yl)benzenesulfonamide (**3c**)

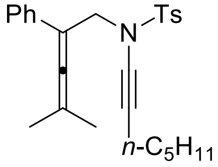

3c

76% isolated yield, colorless oil. ^{1}H NMR (400 MHz, $CDCl_3$) δ ppm 7.77 (d, *J* = 8.4 Hz, 2H), 7.39 (d, *J* = 7.1 Hz, 2H), 7.33 – 7.28 (m, 4H), 7.19 (t, *J* = 7.2 Hz, 1H), 4.25 (s, 2H), 2.45 (s, 3H), 2.20 (t, *J* = 7.0 Hz, 2H), 1.77 (s, 6H), 1.44 – 1.38 (m, 2H), 1.30 – 1.24 (m, 4H), 0.87 (t, *J*

= 7.0 Hz, 3H); ^{13}C NMR (100 MHz, $CDCl_3$) δ 204.39, 144.15, 135.46, 134.45, 129.52, 128.34, 127.77, 126.63, 126.21, 99.84, 98.28, 72.92, 70.66, 52.56, 30.90, 28.64, 22.18, 21.60, 20.12, 18.51, 13.95; HRMS (ESI): calc. for $C_{26}H_{31}NNaO_2S$ $[M+Na]^+$ 444.1968; found: 444.1971.

N-(2,4-dimethylpenta-2,3-dien-1-yl)-N-(hept-1-yn-1-yl)-4-methylbenzenesulfonamide (**3d**)

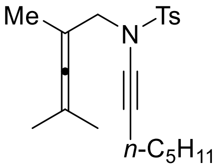

3d

81% isolated yield, colorless oil. ^{1}H NMR (400 MHz, $CDCl_3$) δ ppm 7.75 (d, J = 8.4 Hz, 2H), 7.29 (d, J = 8.1 Hz, 2H), 3.75 (s, 2H), 2.41 (s, 3H), 2.22 (t, J = 7.0 Hz, 2H), 1.61 (s, 3H), 1.60 (s, 6H), 1.48 – 1.41 (m, 2H), 1.33 – 1.24 (m, 4H), 0.86 (t, J = 7.0 Hz, 3H); ^{13}C NMR (100 MHz, $CDCl_3$) δ 201.58, 144.02, 134.70, 129.40, 127.48, 95.49, 91.65, 72.89, 70.00, 56.10, 30.79, 28.57, 22.05, 21.43, 20.43, 18.34, 16.37, 13.84; HRMS (ESI): calc. for $C_{21}H_{29}NNaO_2S$ $[M+Na]^+$ 382.1811; found: 382.1813.

Ethyl 3-(((N-(hept-1-yn-1-yl)-4-methylphenyl)sulfonamido)methyl)-5-methylhexa-3,4-dieno-ate (**3e**)

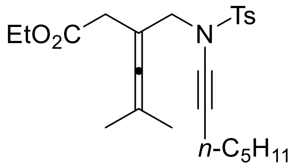

3e

72% isolated yield, colorless oil. ^{1}H NMR (400 MHz, $CDCl_3$) δ ppm 7.77 (d, J = 8.1 Hz, 2H), 7.31 (d, J = 8.0 Hz, 2H), 4.14 (q, J = 7.1 Hz, 2H), 3.89 (s, 2H), 2.98 (s, 2H), 2.43 (s, 3H), 2.23 (t, J = 7.0 Hz, 2H), 1.65 (s, 6H), 1.49 – 1.42 (m, 2H), 1.31 – 1.29 (m, 4H), 1.26 (t, J = 7.1 Hz, 3H), 0.88 (t, J = 7.0 Hz, 3H); ^{13}C NMR (100 MHz, $CDCl_3$) δ 202.96, 171.18, 144.18, 134.68, 129.54, 127.64, 97.88, 90.72, 72.96, 70.30, 60.60, 54.80, 35.92, 30.94, 28.66, 22.17, 21.59, 20.26, 18.45, 14.17, 13.96; HRMS (ESI): calc. for $C_{24}H_{33}NNaO_4S$ $[M+Na]^+$ 454.2023; found: 454.2021.

N-(2-(cyclopentylidenemethylene)hexyl)-N-(hept-1-yn-1-yl)-4-methylbenzenesulfonamide (**3f**)

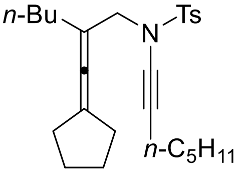

3f

78% isolated yield, colorless oil. ^{1}H NMR (500 MHz, $CDCl_3$) δ ppm 7.77 (d, *J* = 8.3 Hz, 2H), 7.30 (d, *J* = 8.0 Hz, 2H), 3.81 (s, 2H), 2.42 (s, 3H), 2.29 – 2.26 (m, 4H), 2.23 (t, *J* = 7.0 Hz, 2H), 1.90 (t, *J* = 7.0 Hz, 2H), 1.64 – 1.61 (m, 4H), 1.48 – 1.42 (m, 2H), 1.35 – 1.25 (m, 8H), 0.88 (t, *J* = 7.1 Hz, 3H), 0.86 (t, *J* = 7.1 Hz, 3H); ^{13}C NMR (126 MHz, $CDCl_3$) δ 196.63, 143.98, 134.92, 129.41, 127.59, 105.60, 99.12, 73.14, 70.09, 55.30, 31.06, 30.87, 29.53, 29.50, 28.66, 26.94, 22.17, 22.15, 21.51, 18.45, 13.92, 13.90; HRMS (ESI): calc. for $C_{26}H_{37}NNaO_2S$ $[M+Na]^+$ 450.2437; found: 450.2436.

4-methyl-N-(2-(2-methylprop-1-en-1-ylidene)hexyl)-N-(phenylethynyl)benzenesulfonamide (**3g**)

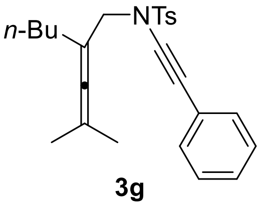

3g

61% isolated yield, colorless oil. ^{1}H NMR (500 MHz, $CDCl_3$) δ ppm 7.78 (d, *J* = 8.1 Hz, 2H), 7.31 (d, *J* = 8.3 Hz, 2H), 3.78 (s, 2H), 2.43 (s, 3H), 1.90 (t, *J* = 7.2 Hz, 2H), 1.71 (dd, *J* = 21.8, 13.4 Hz, 3H), 1.63 (s, 6H), 1.45 – 1.19 (m, 11H), 0.87 (t, *J* = 7.1 Hz, 3H); ^{13}C NMR (126 MHz, $CDCl_3$) δ 201.63, 144.58, 134.98, 131.43, 129.83, 128.33, 127.84, 127.68, 123.34, 97.56, 96.61, 82.75, 70.96, 55.35, 29.76, 22.35, 21.78, 20.75, 14.11; HRMS (ESI): calc. for $C_{25}H_{29}NNaO_2S$ $[M+Na]^+$ 430.1811; found: 430.1810.

N-((4-methoxyphenyl)ethynyl)-4-methyl-N-(2-(2-methylprop-1-en-1-ylidene)hexyl)benzenesu-lfonamide (**3h**)

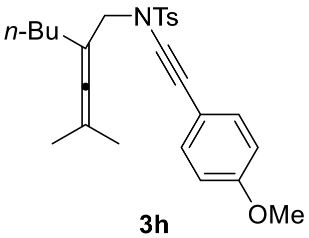

3h

55% isolated yield, colorless oil. ^{1}H NMR (500 MHz, $CDCl_3$) δ ppm 7.86 (d, *J* = 8.3 Hz, 2H), 7.36 (d, *J* = 8.4 Hz, 2H), 7.33 – 7.29 (m, 2H), 6.83 (d, *J* = 8.6 Hz, 2H), 3.92 (s, 2H), 3.83 (s, 3H), 2.47 (s, 3H), 1.96 (q, *J* = 6.7 Hz, 2H), 1.63 (s, 6H), 1.40 – 1.30 (m, 4H), 0.90 (t, *J* = 7.2 Hz, 3H); ^{13}C NMR (126 MHz, $CDCl_3$) δ 201.69, 159.43, 144.46, 134.99, 133.43, 130.72, 129.77, 129.18, 128.35, 127.65, 113.96, 97.42, 96.62, 81.17, 70.52, 55.41 , 29.77, 22.35, 21.77, 20.72, 14.11; HRMS (ESI): calc. for $C_{26}H_{31}NNaO_3S$ $[M+Na]^+$ 460.1917; found: 460.1919.

N-(cyclohexylethynyl)-4-methyl-N-(2-(2-methylprop-1-en-1-ylidene)hexyl)benzenesulfonami-de (**3i**)

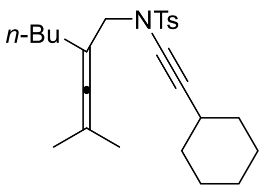

3i

50% isolated yield, colorless oil. ^{1}H NMR (500 MHz, $CDCl_3$) δ ppm 7.78 (d, *J* = 8.1 Hz, 2H), 7.31 (d, *J* = 8.3 Hz, 2H), 3.78 (s, 2H), 2.43 (s, 3H), 1.90 (t, *J* = 7.2 Hz, 2H), 1.71 (dd, *J* = 21.8, 13.4 Hz, 3H), 1.63 (s, 6H), 1.45 – 1.19 (m, 11H), 0.87 (t, *J* = 7.1 Hz, 3H); ^{13}C NMR (126 MHz, $CDCl_3$) δ 201.43, 144.17, 134.93, 129.56, 127.83, 96.98, 74.18, 73.61, 55.29, 33.02, 29.73, 29.48, 28.98, 26.05, 24.83, 22.34, 21.72, 20.71, 14.09; HRMS (ESI): calc. for $C_{25}H_{35}NNaO_2S$ $[M+Na]^+$ 436.2281; found: 436.2280.

4-Methyl-N-(2-(2-methylprop-1-en-1-ylidene)hexyl)-N-((trimethylsilyl)ethynyl)benzenesulfon-amide (**3j**)

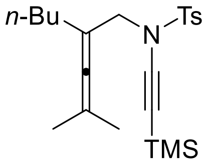

3j

71% isolated yield, colorless oil. ^{1}H NMR (500 MHz, $CDCl_3$) δ ppm 7.77 (d, *J* = 8.3 Hz, 2H), 7.31 (d, *J* = 8.0 Hz, 2H), 3.82 (s, 2H), 2.43 (s, 3H), 1.87 (t, *J* = 7.0 Hz, 2H), 1.63 (s, 6H), 1.36 – 1.25 (m, 4H), 0.86 (t, *J* = 7.1 Hz, 3H), 0,13 (s, 9H); ^{13}C NMR (126 MHz, $CDCl_3$) δ 201.29, 144.36, 134.70, 129.43, 127.73, 97.10, 96.32, 95.14, 77.26, 77.00, 76.75, 72.92, 54.93, 29.52, 29.31, 22.13, 21.55, 20.55, 13.89, 0.11; HRMS (ESI): calc. for $C_{22}H_{33}NNaO_2SSi$ $[M+Na]^+$ 426.1893; found: 426.1895.

N-(3-((tert-butyldimethylsilyl)oxy)prop-1-yn-1-yl)-4-methyl-N-(2-(2-methylprop-1-en-1-ylidene)hexyl)benzenesulfonamide (**3k**)

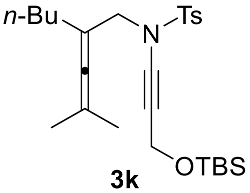

3k

83% isolated yield, colorless oil. ^{1}H NMR (500 MHz, $CDCl_3$) δ ppm 7.78 (d, *J* = 8.4 Hz, 2H), 7.31 (d, *J* = 8.0 Hz, 2H), 4.42 (s, 2H), 3.83 (s, 2H), 2.43 (s, 3H), 1.88 (t, *J* = 6.9 Hz, 2H), 1.64 (s, 6H), 1.37 – 1.27 (m, 4H), 0.87 (s, 9H), 0.06 (s, 6H); ^{13}C NMR (126 MHz, $CDCl_3$) δ 201.40, 144.30, 135.01, 129.62, 127.61, 97.29, 96.43, 78.06, 77.25, 77.00, 76.75, 70.07, 54.99, 51.83,

29.55, 29.38, 25.76, 22.19, 21.57, 20.54, 18.18, 13.94, -5.17; HRMS (ESI): calc. for $C_{26}H_{41}NNaO_3SSi$ $[M+Na]^+$ 498.2469; found: 498.2471.

Optimization of reaction conditions

Table S2. Optimization of reaction conditions for the Alder-ene reaction of **3a**.[a]

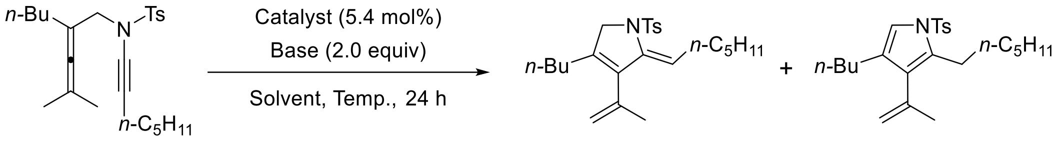

3a **4a** **5a**

Entry	Catalyst	Base	Solvent	T (°C)	Yield of **4a** (%)[b]	Yield of **5a** (%)[b]
1	Cu-AmP-MCC	Cs_2CO_3	Toluene	60	65	0
2	Cu-AmP-MCC	-	Toluene	60	61	<5
3	Cu-AmP-CPG	Cs_2CO_3	Toluene	60	61	0
4[c]	$Cu(OTf)_2$	Cs_2CO_3	Toluene	60	63	0
5[c]	AgOTf	Cs_2CO_3	Toluene	60	48	0
6[c]	$Sc(OTf)_3$	Cs_2CO_3	Toluene	60	53	0
7	-	Cs_2CO_3	Toluene	60	9	0
8	Cu-AmP-MCC	K_2CO_3	Toluene	60	57	0
9	Cu-AmP-MCC	K_3PO_4	Toluene	60	49	0
10	Cu-AmP-MCC	Na_2CO_3	Toluene	60	42	0
11	Cu-AmP-MCC	Et_3N	Toluene	60	64	0
12	Cu-AmP-MCC	DIPEA	Toluene	60	61	0
13	Cu-AmP-MCC	Cs_2CO_3	THF	60	36	0
14	Cu-AmP-MCC	Cs_2CO_3	MeOH	60	21	0
15	Cu-AmP-MCC	Cs_2CO_3	CH_3CN	60	18	0
16	Cu-AmP-MCC	Cs_2CO_3	DCE	60	43	8
17	Cu-AmP-MCC	-	DCE	60	28	19
18	Cu-AmP-MCC	-	$CHCl_3$	60	18	35
19	Cu-AmP-MCC	Cs_2CO_3	Toluene	80	91(88)[d]	0
20[e]	Cu-AmP-MCC	Cs_2CO_3	Toluene	80	78	0
21	Cu-AmP-MCC	-	$CHCl_3$	80	3	71 (68)[d]
22	-	Cs_2CO_3	Toluene	80	29	0
23	-	-	Toluene	80	18	8

[a] The reaction was carried out in the indicated solvent (1 mL) using **3a** (0.1 mmol) and base (0.2 mmol) in the presence of metal catalyst (5.4 mol%). [b] Determined by NMR using 1,1,2,2-tetrachloroethane as the standard. [c] 5.0 mol%) metal catalyst was used [d] Isolated yield. [e] Reaction time: 12 h. CPG = controlled pore glass.

General procedure for the Cu-AmP-MCC-catalyzed Alder-ene reaction of 3 to 2,5-dihydropyrroles 4

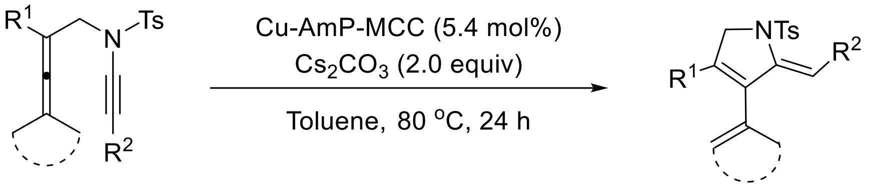

To an oven-dried microwave vial equipped with a magnetic stir bar were added Cu-AmP-MCC (8.0 mg, 5.4 mol% of Cu) and Cs_2CO_3 (130.3 mg, 0.40 mmol, 2.0 equiv). The vial was then sealed and evacuated under vacuum and flushed with argon three times before the solution of allenynamide **3** (0.20 mmol, 1.0 equiv) in toluene (2.0 mL) was added. The reaction mixture was stirred under argon at 80 °C. After 24 h, the reaction mixture was cooled to rt, filtered through a pad of celite, concentrated in vacuo and purified by flash column chromatography (petroleum ether/EtOAc as eluent) to give 2,5-dihydropyrroles **4**.

(Z)-4-Butyl-2-hexylidene-3-(prop-1-en-2-yl)-1-tosyl-2,5-dihydro-1H-pyrrole (**4a**)

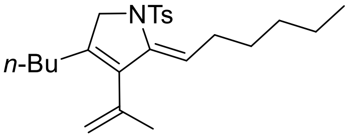

4a

The general procedure was followed using **3a** (80.2 mg, 0.2 mmol). Purification by column chromatography on silica gel (petroleum ether/EtOAc = 30 : 1) yielded **4a** (70.6 mg, 88%) as a colorless oil. ^{1}H NMR (400 MHz, $CDCl_3$) δ ppm 7.64 (d, *J* = 8.2 Hz, 2H), 7.20 (d, *J* = 7.8Hz, 2H), 5.07 – 5.06 (m, 1H), 5.04 (t, *J* = 7.3 Hz, 1H), 4.57 – 4.56 (m, 1H), 4.11 (s, 2H), 2.52 (dt, *J* = 7.5, 7.3 Hz, 2H), 2.37 (s, 3H), 1.88 (t, *J* = 7.0 Hz, 2H), 1.59 (s, 3H), 1.45 – 1.38 (m, 2H), 1.34 – 1.30 (m, 4H), 1.02 – 0.92 (m, 4H), 0.89 (t, *J* = 7.0 Hz, 3H), 0.75 (t, *J* = 6.9 Hz, 3H); ^{13}C NMR (100 MHz, $CDCl_3$) δ 143.58, 142.44, 138.26, 137.54, 134.99, 133.77, 129.00, 127.95, 118.12, 116.98, 58.10, 31.67, 30.06, 29.78, 29.27, 26.29, 22.57, 22.43, 22.24, 21.46, 14.07, 13.85; HRMS (ESI): calc. for $C_{24}H_{35}NNaO_2S$ $[M+Na]^+$ 424.2281; found: 424.2282.

(Z)-2-Hexylidene-4-phenethyl-3-(prop-1-en-2-yl)-1-tosyl-2,5-dihydro-1H-pyrrole (**4b**)

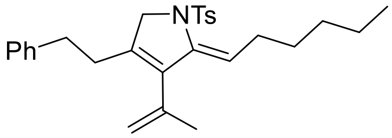

4b

The general procedure was followed using **3b** (89.8 mg, 0.2 mmol). Purification by column chromatography on silica gel (petroleum ether/EtOAc = 20 : 1) yielded **4b** (76.3 mg, 85%) as a colorless oil. ^{1}H NMR (500 MHz, $CDCl_3$) δ ppm 7.67 (d, *J* = 8.2 Hz, 2H), 7.29 – 7.20 (m, 5H), 7.05 (d, *J* = 7.1 Hz, 2H), 5.14 – 5.05 (m, 2H), 4.53 (d, *J* = 1.0 Hz, 1H), 4.22 (s, 2H), 2.62 – 2.51 (m, 2H), 2.41 (s, 3H), 2.36 – 2.29 (m, 2H), 2.26 – 2.18 (m, 2H), 1.58 (d, *J* = 13.4 Hz, 3H), 1.50 – 1.42 (m, 2H), 1.39 – 1.32 (m, 4H), 0.96 – 0.90 (m, 3H); ^{13}C NMR (126 MHz, $CDCl_3$) δ 143.82, 142.34, 141.20, 139.03, 137.50, 134.23, 133.89, 129.20, 128.60, 128.15, 126.33, 118.87, 117.28, 58.38, 34.52, 31.82, 29.89, 29.39, 28.95, 22.73, 22.53, 21.67, 14.26; HRMS (ESI): calc. for $C_{28}H_{35}NNaO_2S$ $[M+Na]^+$ 472.2281; found: 472.2280.

(Z)-2-Hexylidene-4-phenyl-3-(prop-1-en-2-yl)-1-tosyl-2,5-dihydro-1H-pyrrole (**4c**)

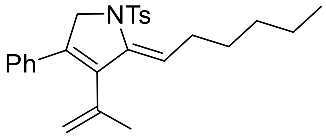

4c

The general procedure was followed using **3c** (84.2 mg, 0.2 mmol). Purification by column chromatography on silica gel (petroleum ether/EtOAc = 20 : 1) yielded **4c** (75.8 mg, 90%) as a colorless oil. ^{1}H NMR (400 MHz, $CDCl_3$) δ ppm 7.62 (d, *J* = 8.2 Hz, 2H), 7.26 – 7.20 (m, 3H), 7.15 (d, *J* = 7.8 Hz, 2H), 7.13 – 7.10 (m, 2H), 5.35 (t, *J* = 7.4 Hz, 1H), 5.16 – 5.15 (m, 1H), 4.67 – 4.66 (m, 1H), 4.60 (s, 2H), 2.60 (dt, *J* = 7.5, 7.3 Hz, 2H), 2.34 (s, 3H), 1.53 (s, 3H), 1.49 – 1.42 (m, 2H), 1.37 – 1.33 (m, 4H), 0.91 (t, *J* = 7.1 Hz, 3H); ^{13}C NMR (100 MHz, $CDCl_3$) δ 143.73, 143.35, 138.69, 138.29, 133.55, 133.45, 131.29, 129.08, 128.26, 127.85, 127.69, 126.51, 120.73, 118.04, 58.19, 31.68, 29.67, 29.37, 22.57, 22.00, 21.46, 14.09; HRMS (ESI): calc. for $C_{26}H_{31}NNaO_2S$ $[M+Na]^+$ 444.1968; found: 444.1969.

(Z)-2-Hexylidene-4-methyl-3-(prop-1-en-2-yl)-1-tosyl-2,5-dihydro-1H-pyrrole (**4d**)

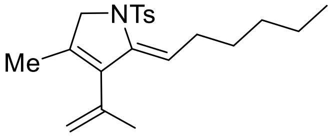

4d

The general procedure was followed using **3d** (71.8 mg, 0.2 mmol). Purification by column chromatography on silica gel (petroleum ether/EtOAc = 30 : 1) yielded **4d** (58.2 mg, 81%) as a colorless oil. ^{1}H NMR (400 MHz, $CDCl_3$) δ ppm 7.61 (d, *J* = 8.3 Hz, 2H), 7.19 (d, *J* = 7.9 Hz, 2H), 5.07 – 5.05 (m, 1H), 5.02 (d, *J* = 7.3 Hz, 1H), 5.54 – 5.53 (m, 1H), 4.12 (s, 2H), 2.51 (dt, *J* = 7.5, 7.2 Hz, 2H), 2.37 (s, 3H), 1.52 (s, 3H), 1.46 (s, 3H), 1.42 – 1.37 (m, 2H), 1.35 – 1.29 (m, 4H), 0.88 (t, *J* = 7.0 Hz, 3H); ^{13}C NMR (100 MHz, $CDCl_3$) δ 143.55, 142.24, 138.15, 137.37, 133.73, 130.83, 128.98, 127.76, 118.14, 117.04, 60.00, 31.64, 29.73, 29.11, 22.54, 22.05, 21.47, 14.06, 11.90; HRMS (ESI): calc. for $C_{21}H_{29}NNaO_2S$ $[M+Na]^+$ 382.1811; found: 382.1810.

Ethyl (Z)-2-(5-hexylidene-4-(prop-1-en-2-yl)-1-tosyl-2,5-dihydro-1H-pyrrol-3-yl)acetate (**4e**)

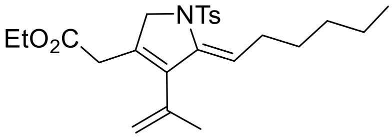

4e

The general procedure was followed using **3e** (86.2 mg, 0.2 mmol). Purification by column chromatography on silica gel (petroleum ether/EtOAc = 10 : 1) yielded **4e** (41.5 mg, 48%) as a colorless oil. ^{1}H NMR (500 MHz, $CDCl_3$) δ ppm 7.63 (d, *J* = 8.3 Hz, 2H), 7.20 (d, *J* = 8.0 Hz, 2H), 5.11 – 5.08 (m, 2H), 4.60 – 4.59 (m, 1H), 4.33 (s, 2H), 4.06 (q, *J* = 7.2 Hz, 2H), 2.92 (s, 2H), 2.52 (dt, *J* = 7.5, 7.4 Hz, 2H), 2.38 (s, 3H), 1.52 (s, 3H), 1.45 – 1.39 (m, 2H), 1.35 – 1.31 (m, 4H), 1.22 (t, *J* = 7.1 Hz, 3H), 1.22 (t, *J* = 7.1 Hz, 3H), 0.89 (t, *J* = 7.1 Hz, 3H); ^{13}C NMR (126 MHz, $CDCl_3$) δ 169.49, 143.58, 141.60, 141.52, 136.78, 133.77, 129.01, 127.94, 126.93, 120.06, 117.87, 60.93, 58.53, 32.99, 31.66, 29.63, 29.19, 22.55, 22.14, 21.50, 14.13, 14.07; HRMS (ESI): calc. for $C_{24}H_{33}NNaO_4S$ $[M+Na]^+$ 454.2023; found: 454.2023.

(Z)-4-Butyl-3-(cyclopent-1-en-1-yl)-2-hexylidene-1-tosyl-2,5-dihydro-1H-pyrrole (**4f**)

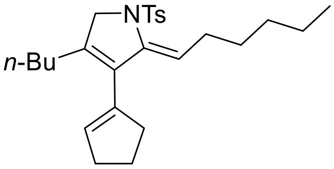

4f

The general procedure was followed using **3f** (85.5 mg, 0.2 mmol). Purification by column chromatography on silica gel (petroleum ether/EtOAc = 30 : 1) yielded **4f** (66.6 mg, 78%) as a colorless oil. ^{1}H NMR (500 MHz, $CDCl_3$) δ ppm 7.62 (d, *J* = 8.3 Hz, 2H), 7.19 (d, *J* = 8.1 Hz, 2H), 5.45 – 5.43 (m, 1H), 5.05 (t, *J* = 7.3 Hz, 1H), 4.08 (s, 2H), 2.51 (dt, *J* = 7.5, 7.4 Hz, 2H),

2.41 – 2.37 (m, 2H), 2.38 (s, 3H), 2.19 – 2.15 (m, 2H), 2.19 – 1.84 (m, 4H), 1.44 – 1.38 (m, 2H), 1.34 – 1.31 (m, 4H), 0.94 – 0.86 (m, 7H), 0.73 (t, *J* = 6.9 Hz, 3H); ^{13}C NMR (126 MHz, $CDCl_3$) δ 143.48, 142.76, 135.85, 135.58, 133.77, 133.69, 131.43, 128.95, 127.92, 118.45, 58.04, 35.36, 32.93, 31.71, 30.08, 29.79, 29.33, 26.45, 23.56, 22.57, 22.18, 21.48, 14.07, 13.83; HRMS (ESI): calc. for $C_{26}H_{37}NNaO_2S$ $[M+Na]^+$ 450.2437; found: 450.2434.

(Z)-2-Benzylidene-4-butyl-3-(prop-1-en-2-yl)-1-tosyl-2,5-dihydro-1H-pyrrole (**4g**)

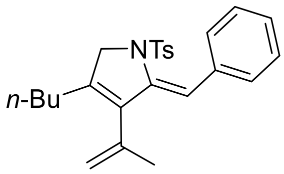

4g

The general procedure was followed using **3g** (81.4 mg, 0.2 mmol). The reaction was performed for 4 h. Purification by column chromatography on silica gel (petroleum ether/EtOAc = 20 : 1) yielded **4g** (62.7 mg, 77%) as a colorless oil. The NMR data are in accordance with those reported in the literature;[3] ^{1}H NMR (500 MHz, $CDCl_3$) δ ppm 7.78 (d, *J* = 7.3 Hz, 2H), 7.67 (d, *J* = 8.3 Hz, 2H), 7.33 (t, *J* = 7.7 Hz, 2H), 7.23 (d, *J* = 8.0 Hz, 2H), 7.19 (t, *J* = 7.4 Hz, 1H), 6.01 (s, 1H), 5.18 (s, 1H), 4.65 (s, 1H), 4.25 (s, 2H), 2.39 (s, 3H), 1.95 (t, *J* = 7.3 Hz, 2H), 1.72 (s, 3H), 1.03 – 0.93 (m, 4H), 0.79 (t, *J* = 7.0 Hz, 3H); ^{13}C NMR (126 MHz, $CDCl_3$) δ 143.98, 143.17, 139.42, 137.80, 137.24, 136.81, 133.15, 129.26, 129.11, 128.17, 127.75, 126.62, 117.68, 115.04, 58.38, 30.18, 26.57, 22.72, 22.33, 21.53, 13.93; HRMS (ESI): calc. for $C_{25}H_{29}NNaO_2S$ $[M+Na]^+$ 430.1811; found: 430.1814.

(Z)-4-Butyl-2-(4-methoxybenzylidene)-3-(prop-1-en-2-yl)-1-tosyl-2,5-dihydro-1H-pyrrole (**4h**)

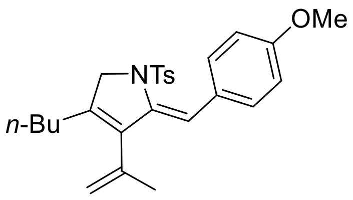

4h

The general procedure was followed using **3h** (87.4 mg, 0.2 mmol). The reaction was performed for 5 h. Purification by column chromatography on silica gel (petroleum ether/EtOAc = 20 : 1) yielded **4h** (65.6 mg, 75%) as a colorless oil. ^{1}H NMR (500 MHz, $CDCl_3$) δ ppm 7.71 (d, *J* = 8.8 Hz, 2H), 7.66 (d, *J* = 8.2 Hz, 2H), 7.22 (d, *J* = 8.4 Hz, 2H), 6.86 (d, *J* = 8.7 Hz, 2H), 5.93 (s, 1H), 5.15 (d, *J* = 1.5 Hz, 1H), 4.61 (s, 1H), 4.22 (s, 2H), 3.81 (s, 3H), 2.38

(s, 3H), 1.91 (t, J = 7.0 Hz, 2H), 1.68 (s, 3H), 1.04 – 0.90 (m, 4H), 0.77 (t, J = 6.8 Hz, 3H); ^{13}C NMR (126 MHz, $CDCl_3$) δ 158.44, 143.97, 141.58, 139.57, 137.46, 136.71, 133.30, 130.70, 129.36, 129.15, 128.33, 117.62, 115.12, 58.46, 55.28, 30.30, 26.65, 22.81, 22.45, 21.61, 14.00; HRMS (ESI): calc. for $C_{26}H_{31}NNaO_3S$ $[M+Na]^+$ 460.1917; found: 460.1916.

(Z)-4-Butyl-2-(cyclohexylmethylene)-3-(prop-1-en-2-yl)-1-tosyl-2,5-dihydro-1H-pyrrole (**4i**)

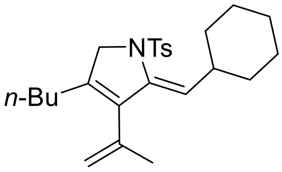

4i

The general procedure was followed using **3i** (82.6 mg, 0.2 mmol). Purification by column chromatography on silica gel (petroleum ether/EtOAc = 30 : 1) yielded **4i** (57.9 mg, 70%) as a colorless oil. The NMR data are in accordance with those reported in the literature;[3] ^{1}H NMR (500 MHz, $CDCl_3$) δ ppm 7.64 (d, J = 8.2 Hz, 2H), 7.20 (d, J = 8.3 Hz, 2H), 5.05 (s, 1H), 4.87 (d, J = 10.5 Hz, 1H), 4.55 (d, J = 1.0 Hz, 1H), 4.11 (s, 2H), 3.02 – 2.94 (m, 1H), 1.87 (t, J = 7.4 Hz, 4H), 1.72 – 1.65 (m, 3H), 1.57 (s, 3H), 1.39 (dt, J = 22.2, 9.6 Hz, 2H), 1.21 – 1.13 (m, 1H), 1.10 – 0.82 (m, 7H), 0.75 (t, J = 7.1 Hz, 3H); ^{13}C NMR (126 MHz, $CDCl_3$) δ 143.69, 140.66, 138.51, 137.74, 135.12, 133.98, 129.17, 128.08, 123.86, 117.14, 58.36, 37.33, 33.60, 30.22, 26.46, 25.96, 22.62, 22.44, 21.64, 14.03; HRMS (ESI): calc. for $C_{25}H_{35}NNaO_2S$ $[M+Na]^+$ 436.2281; found: 436.2276.

(Z)-4-Butyl-3-(prop-1-en-2-yl)-1-tosyl-2-((trimethylsilyl)methylene)-2,5-dihydro-1H-pyrrole (**4j**)

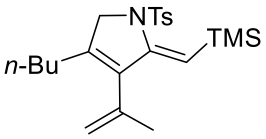

4j

The general procedure was followed using **3j** (80.6 mg, 0.2 mmol). Purification by column chromatography on silica gel (petroleum ether/EtOAc = 30 : 1) yielded **4j** (66.1 mg, 82%) as a colorless oil. ^{1}H NMR (400 MHz, $CDCl_3$) δ ppm 7.62 (d, J = 8.3 Hz, 2H), 7.20 (d, J = 7.8 Hz, 2H), 5.12 – 5.10 (m, 1H), 4.93 (s, 1H), 4.56 – 4.55 (m, 1H), 4.08 (s, 2H), 2.37 (s, 3H), 1.92 (t, J = 7.6 Hz, 2H), 1.61 (s, 3H), 1.01 – 0.97 (m, 2H), 0.90 – 0.82 (m, 2H), 0.74 (t, J = 7.1 Hz, 4H), 0.25 (s, 9H); ^{13}C NMR (100 MHz, $CDCl_3$) δ 154.40, 143.64, 139.95, 138.08, 137.64,

133.74, 129.01, 127.91, 117.21, 111.73, 57.40, 29.99, 26.56, 22.56, 22.12, 21.48, 13.85, 0.43; HRMS (ESI): calc. for $C_{22}H_{33}NNaO_2SSi$ $[M+Na]^+$ 426.1893; found: 426.1899.

(Z)-4-Butyl-2-(2-((tert-butyldimethylsilyl)oxy)ethylidene)-3-(prop-1-en-2-yl)-1-tosyl-2,5-dihydro-1H-pyrrole (**4k**)

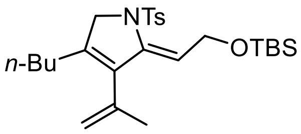

4k

The general procedure was followed using **3k** (95.1 mg, 0.2 mmol). Purification by column chromatography on silica gel (petroleum ether/EtOAc = 20 : 1) yielded **4k** (69.4 mg, 73%) as a colorless oil. ^{1}H NMR (400 MHz, $CDCl_3$) δ ppm 7.60 (d, J = 7.6 Hz, 2H), 7.21 (d, J = 7.9 Hz, 2H), 5.19 (t, J = 6.0 Hz, 1H), 5.10 (s, 1H), 4.63 (d, J = 6.0 Hz, 2H), 4.60 (s, 1H), 4.09 (s, 2H), 2.38 (s, 3H), 1.90 (t, J = 7.2 Hz, 2H), 1.62 (s, 3H), 1.00 – 0.86 (m, 4H), 0.90 (s, 9H), 0.74 (t, J = 7.3 Hz, 3H), 0.08 (s, 6H); ^{13}C NMR (100 MHz, $CDCl_3$) δ 143.89, 142.46, 137.99, 136.96, 136.74, 133.40, 129.12, 127.96, 117.44, 116.44, 61.53, 58.01, 30.06, 26.32, 26.00, 22.39, 22.16, 21.49, 18.35, 13.84, -5.01; HRMS (ESI): calc. for $C_{26}H_{41}NNaO_3SSi$ $[M+Na]^+$ 498.2469; found: 498.2470.

General procedure for the Cu-AmP-MCC-catalyzed Alder-ene reaction of 3 to pyrroles 5

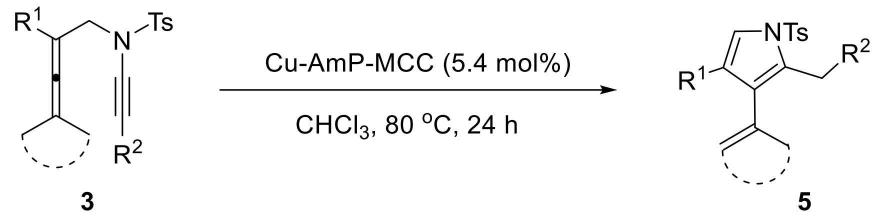

To an oven-dried microwave vial equipped with a magnetic stir bar were added Cu-AmP-MCC (8.0 mg, 5.4 mol% of Cu). The vial was then sealed and evacuated under vacuum and flushed with argon for three times before the solution of allenynamide **3** (0.20 mmol, 1.0 equiv) in $CHCl_3$ (2.0 mL) was added. The reaction mixture was stirred under argon at 80 °C. After 24 h, the reaction mixture was cooled to rt, filtered through a pad of celite, concentrated in vacuo and purified by flash column chromatrography (petroleum ether/EtOAc as eluent) to give pyrroles **5**.

4-Butyl-2-hexyl-3-(prop-1-en-2-yl)-1-tosyl-1H-pyrrole (**5a**)

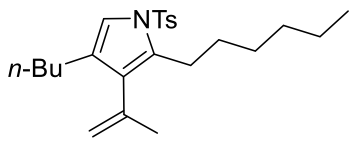

5a

The general procedure was followed using **3a** (80.2 mg, 0.2 mmol). Purification by column chromatography on silica gel (petroleum ether/EtOAc = 50 : 1) yielded **5a** (54.5 mg, 68%) as a colorless oil. ^{1}H NMR (400 MHz, $CDCl_3$) δ ppm 7.59 (d, J = 8.1 Hz, 2H), 7.26 (d, J = 7.9 Hz, 2H), 6.96 (s, 1H), 5.14 (s, 1H), 4.73 (s, 1H), 2.57 (t, J = 8.0 Hz, 2H), 2.40 (s, 3H), 2.31 (t, J = 7.6 Hz, 2H), 1.87 (s, 3H), 1.54 – 1.46 (m, 2H), 1.34 – 1.20 (m, 10H), 0.90 (t, J = 7.3 Hz, 3H), 0.86 (t, J = 7.1 Hz, 3H); ^{13}C NMR (100 MHz, $CDCl_3$) δ 144.21, 138.76, 137.09, 131.57, 130.08, 129.74, 126.68, 126.47, 118.33, 116.48, 31.47, 31.39, 31.21, 29.35, 25.85, 25.33, 24.23, 22.58, 22.47, 21.57, 14.07, 13.92; HRMS (ESI): calc. for $C_{24}H_{35}NNaO_2S$ [M+Na]$^+$ 424.2281; found: 424.2280.

2-Hexyl-4-phenethyl-3-(prop-1-en-2-yl)-1-tosyl-1H-pyrrole (**5b**)

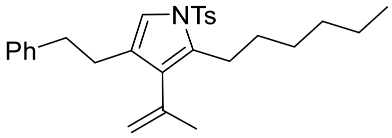

5b

The general procedure was followed using **3b** (89.8 mg, 0.2 mmol). Purification by column chromatography on silica gel (petroleum ether/EtOAc = 30 : 1) yielded **5b** (63.8 mg, 71%) as a colorless oil. ^{1}H NMR (400 MHz, $CDCl_3$) δ ppm 7.54 (d, J = 8.4 Hz, 2H), 7.29 – 7.25 (m, 4H), 7.21 – 7.14 (m, 3H), 6.96 (s, 1H), 5.17 – 5.16 (m, 1H), 4.75 – 4.74 (m, 1H), 2.84 (t, J = 7.3 Hz, 2H), 2.64 (t, J = 8.1 Hz, 2H), 2.57 (t, J = 8.1 Hz, 2H), 2.42 (s, 3H), 1.89 (s, 3H), 1.43 – 1.36 (m, 2H), 1.28 – 1.19 (m, 6H), 0.87 (t, J = 7.1 Hz, 3H); ^{13}C NMR (100 MHz, $CDCl_3$) δ 144.26, 141.88, 138.58, 137.01, 131.61, 129.84, 129.79, 128.38, 128.27, 126.50, 125.84, 125.50, 118.59, 116.77, 35.61, 31.47, 31.16, 29.34, 27.62, 25.81, 24.24, 22.58, 21.59, 14.07; HRMS (ESI): calc. for $C_{28}H_{35}NNaO_2S$ $[M+Na]^+$ 472.2281; found: 472.2279.

2-Hexyl-4-phenyl-3-(prop-1-en-2-yl)-1-tosyl-1H-pyrrole (**5c**)

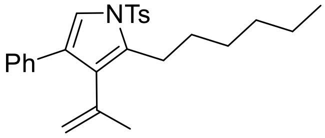

5c

The general procedure was followed using **3c** (84.2 mg, 0.2 mmol). Purification by column chromatography on silica gel (petroleum ether/EtOAc = 20 : 1) yielded **5c** (61.5 mg, 73%) as a colorless oil. ^{1}H NMR (400 MHz, $CDCl_3$) δ ppm 7.69 (d, J = 8.4 Hz, 2H), 7.49 – 7.46 (m, 2H), 7.36 (s, 1H), 7.35 – 7.23 (m, 5H), 5.22 – 5.21 (m, 1H), 4.94 – 4.93 (m, 1H), 2.69 (t, J = 8.0 Hz, 2H), 2.42 (s, 3H), 1.69 (s, 3H), 1.47 – 1.40 (m, 2H), 1.30 – 1.21 (m, 6H), 0.88 (t, J = 7.2 Hz, 3H); ^{13}C NMR (100 MHz, $CDCl_3$) δ 144.67, 139.26, 136.69, 134.36, 132.52, 129.93, 128.44, 128.36, 127.11, 126.73, 126.71, 126.42, 118.91, 116.96, 31.46, 31.29, 29.36, 25.50, 24.15, 22.57, 21.59, 14.07; HRMS (ESI): calc. for $C_{26}H_{31}NNaO_2S$ $[M+Na]^+$ 444.1968; found: 444.1970.

2-hexyl-4-methyl-3-(prop-1-en-2-yl)-1-tosyl-1H-pyrrole (**5d**)

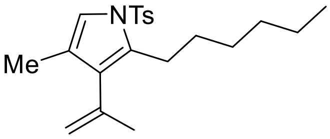

5d

The general procedure was followed using **3d** (71.8 mg, 0.2 mmol). Purification by column chromatography on silica gel (petroleum ether/EtOAc = 30 : 1) yielded **5d** (45.4 mg, 63%) as a colorless oil. ^{1}H NMR (400 MHz, $CDCl_3$) δ ppm 7.61 (d, *J* = 8.4 Hz, 2H), 7.26 (d, *J* = 8.0 Hz, 2H), 6.97 (q, *J* = 1.1 Hz, 1H), 5.16 –5.14 (m, 1H), 4.74 –4.73 (m, 1H), 2.60 (t, *J* = 8.0 Hz, 2H), 2,40 (s, 3H), 1.94 (d, *J* = 1.2 Hz, 3H), 1.88 (s, 3H), 1.44 – 1.37 (m, 2H), 1.29 – 1.19 (m, 6H), 0.86 (t, *J* = 7.2 Hz, 3H); ^{13}C NMR (100 MHz, $CDCl_3$) δ 144.27, 138.62, 137.06, 131.63, 130.37, 129.77, 126.53, 121.35, 118.83, 116.33, 31.46, 31.22, 29.33, 25.91, 23.99, 22.58, 21.55, 14.06, 10.80; HRMS (ESI): calc. for $C_{21}H_{29}NNaO_2S$ $[M+Na]^+$ 382.1811; found: 382.1813.

Ethyl 2-(5-hexyl-4-(prop-1-en-2-yl)-1-tosyl-1H-pyrrol-3-yl)acetate (**5e**)

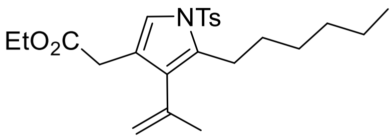

5e

The general procedure was followed using **3e** (86.2 mg, 0.2 mmol). Purification by column chromatography on silica gel (petroleum ether/EtOAc = 15 : 1) yielded **5e** (35.5 mg, 41%) as a colorless oil. ^{1}H NMR (400 MHz, $CDCl_3$) δ ppm 7.62 (d, *J* = 8.1 Hz, 2H), 7.27 (d, *J* = 8.8 Hz, 2H), 7.20 (s, 1H), 5.17 (s, 1H), 4.74 (s, 1H), 4.13 (q, *J* = 7.1 Hz, 2H), 3.35 (s, 2H), 2.56 (t, *J* = 8.0 Hz, 2H), 2.40 (s, 3H), 1.86 (s, 3H), 1.42 – 1.35 (m, 2H), 1.24 (t, *J* = 7.1 Hz, 3H), 1.24 – 1.18 (m, 6H), 0.86 (t, *J* = 7.2 Hz, 3H); ^{13}C NMR (100 MHz, $CDCl_3$) δ 171.33, 144.49, 137.99, 136.88, 131.60, 129.85, 129.55, 126.61, 120.24, 118.11, 117.35, 60.72, 31.44, 31.41, 31.00, 29.34, 25.92, 24.18, 22.56, 21.58, 14.16, 14.06; HRMS (ESI): calc. for $C_{24}H_{33}NNaO_4S$ $[M+Na]^+$ 454.2023; found: 454.2021.

4-Butyl-3-(cyclopent-1-en-1-yl)-2-hexyl-1-tosyl-1H-pyrrole (**5f**)

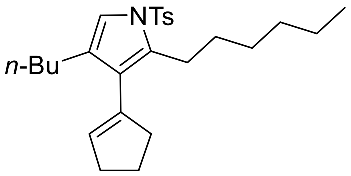

5f

The general procedure was followed using **3f** (85.5 mg, 0.2 mmol). Purification by column chromatography on silica gel (petroleum ether/EtOAc = 30 : 1) yielded **5f** (45.4 mg, 53%) as a colorless oil. ^{1}H NMR (400 MHz, $CDCl_3$) δ ppm 7.60 (d, *J* = 8.4 Hz, 2H), 7.25 (d, *J* = 8.0 Hz, 2H), 6.98 (s, 1H), 5.55 – 5.53 (m, 1H), 2.58 (t, *J* = 8.0 Hz, 2H), 2.45 – 2.40 (m, 4H), 2.40 (s,

3H), 2.31 (t, J = 7.0 Hz, 2H), 1.92 (tt, J = 7.2, 7.2 Hz, 2H), 1.52 – 1.44 (m, 2H), 1.41 – 1.19 (m, 10H), 0.90 (t, J = 7.3 Hz, 3H), 0.86 (t, J = 7.2 Hz, 3H); ^{13}C NMR (100 MHz, $CDCl_3$) δ 144.21, 137.09, 136.79, 132.23, 129.75, 129.68, 127.24, 126.53, 125.00, 118.36, 77.32, 77.00, 76.68, 36.47, 32.93, 31.43, 31.37, 31.10, 29.23, 25.99, 25.77, 23.97, 22.57, 22.50, 21.56, 14.07, 13.95; HRMS (ESI): calc. for $C_{26}H_{37}NNaO_2S$ $[M+Na]^+$ 450.2437; found: 450.2435.

2-Benzyl-4-butyl-3-(prop-1-en-2-yl)-1-tosyl-1H-pyrrole (**5g**)

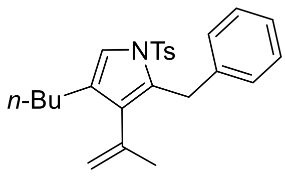

5g

The general procedure was followed using **3g** (81.4 mg, 0.2 mmol). Purification by column chromatography on silica gel (petroleum ether/EtOAc = 20 : 1) yielded **5g** (57.8 mg, 71%) as a colorless oil. ^{1}H NMR (400 MHz, $CDCl_3$) δ ppm 7.28 (d, J = 7.7 Hz, 2H), 7.09 – 7.06 (m, 4H), 7.02 (d, J = 8.1 Hz, 2H), 6.89 – 6.87 (m, 2H), 5.13 (s, 1H), 4.80 (s, 1H), 4.19 (s, 2H), 2.40 (t, J = 7.4 Hz, 2H), 2.34 (s, 3H), 1.87 (s, 3H), 1.58 (tt, J = 7.8, 7.8 Hz, 2H), 1.44 – 1.35 (m, 2H), 0.96 (t, J = 7.3 Hz, 3H); ^{13}C NMR (100 MHz, $CDCl_3$) δ 143.82, 139.75, 138.46, 136.16, 131.65, 129.38, 128.01, 127.96, 127.82, 126.68, 126.33, 125.40, 118.63, 116.76, 31.46, 30.72, 25.44, 24.21, 22.55, 21.47, 13.95; HRMS (ESI): calc. for $C_{25}H_{29}NNaO_2S$ $[M+Na]^+$ 430.1811; found: 430.1810.

4-Butyl-3-(prop-1-en-2-yl)-1-tosyl-2-((trimethylsilyl)methyl)-1H-pyrrole (**5j**)

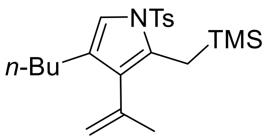

5j

The general procedure was followed using **3j** (80.6 mg, 0.2 mmol). Purification by column chromatography on silica gel (petroleum ether/EtOAc = 30 : 1) yielded **5j** (61.4 mg, 76%) as a colorless oil. ^{1}H NMR (400 MHz, $CDCl_3$) δ ppm 7.55 (d, J = 8.4 Hz, 2H), 7.24 (d, J = 8.0 Hz, 2H), 6.86 (s, 1H), 5.15 – 5.13 (m, 1H), 4.73 – 4.71 (m, 1H), 2.39 (s, 3H), 2.29 (t, J = 7.4 Hz, 2H), 2.22 (s, 2H), 1.86 (s, 3H), 1.45 (tt, J = 7.7, 7.7 Hz, 2H), 1.30 – 1.21 (m, 2H), 0.87 (t, J = 7.4 Hz, 3H), 0.02 (s, 9H); ^{13}C NMR (100 MHz, $CDCl_3$) δ 144.03, 138.89, 136.95, 130.35, 129.61, 128.12, 127.64, 126.31, 117.69, 116.83, 31.21, 25.47, 23.87, 22.31, 21.54, 15.90, 13.90, -0.70; HRMS (ESI): calc. for $C_{22}H_{33}NNaO_2SSi$ $[M+Na]^+$ 426.1893; found: 426.1895.

Deuterium-labeling experiments

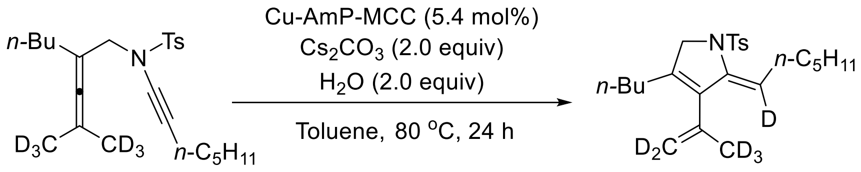

d^6-**3a** *d*^{6}-**4a**

To an oven-dried microwave vial equipped with a magnetic stir bar were added Cu-AmP-MCC (8.0 mg, 5.4 mol% of Cu) and Cs_2CO_3 (130.3 mg, 0.40 mmol, 2.0 equiv). The vial was then sealed and evacuated under vacuum and flushed with argon for three times before H_2O (7.2 μL, 0.40 mmol, 2.0 equiv) and the solution of *d*^{6}-**3a** (81.5 mg, 0.2 mmol, 1.0 equiv) in toluene (2.0 mL) was added. The reaction mixture was stirred under argon at 80 °C. After 24 h, the reaction mixture was cooled to rt, filtered through a pad of celite, concentrated in vacuo and purified by flash column chromatrography (petroleum ether/EtOAc = 30 : 1 as eluent) to give *d*^{6}-**4a**.

(Z)-4-Butyl-2-(hexylidene-1-d)-3-(prop-1-en-2-yl-d5)-1-tosyl-2,5-dihydro-1H-pyrrole (*d*^{6}-**4a**)

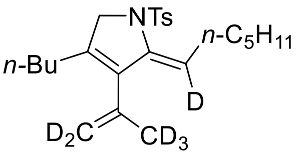

d^6-**4a**

d^6-**4a**: 52.9 mg, 65% isolated yield, colorless oil. ^{1}H NMR (400 MHz, $CDCl_3$) δ ppm 7.63 (d, *J* = 8.0 Hz, 2H), 7.20 (d, *J* = 8.0 Hz, 2H), 4.10 (s, 2H), 2.52 (t, *J* = 7.6 Hz, 2H), 2.37 (s, 3H), 1.88 (t, *J* = 7.5 Hz, 2H), 1.43 – 1.38 (m, 2H), 1.34 – 1.31 (m, 4H), 1.01 – 0.91 (m, 4H), 0.89 (t, *J* = 7.1 Hz, 3H), 0.75 (t, *J* = 6.9 Hz, 3H); ^{13}C NMR (100 MHz, $CDCl_3$) δ 143.57, 142.40, 138.18, 137.23, 134.94, 133.75, 128.99, 127.93, 117.73 (t, *J* = 22.0 Hz), 116.68 – 116.20 (m), 58.09, 31.67, 30.06, 29.74, 29.16, 26.27, 22.56, 22.22, 21.46, 14.07, 13.85. HRMS (ESI): calc. for $C_{24}H_{29}D_6NNaO_2S$ $[M+Na]^+$ 430.2657; found: 430.2658.

Recycling experiments and leaching test

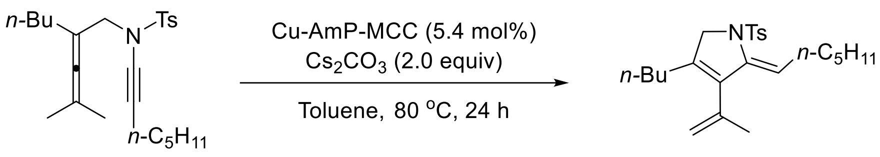

To an oven-dried microwave vial equipped with a magnetic stir bar were added Cu-AmP-MCC (8.0 mg, 5.4 mol% of Cu) and Cs_2CO_3 (130.3 mg, 0.40 mmol, 2.0 equiv). The vial was then sealed and evacuated under vacuum and flushed with argon three times before the solution of **3a** (80.2 mg, 0.2 mmol, 1.0 equiv) in toluene (2.0 mL) was added. The reaction mixture was stirred under argon at 80 °C. After 24 h, the reaction mixture was cooled to rt and centrifuged for 3 min at 10000 rpm. The catalyst was washed with toluene (2 × 2 mL), H_2O (2 × 2 mL), and acetone (3 × 2 mL) and dried for 12 h under vacuum before being used in the next run or for characterizations. The supernatant of toluene was combined for the determination of yield by NMR using 1,1,2,2-tetrachloroethane as the internal standard. The leaching test of the copper nanocatalyst was carried out by ICP-OES analysis of the recovered solution from the 1st run and the amount of copper in the reaction mixture was determined to be <1 ppm.

References

[1] (a) Li, M.-B.; Yang, Y.; Rafi, A. A.; Oschmann, M.; Grape, E. S.; Inge, A. K.; Córdova, A.; Bäckvall, J.-E. Silver-Triggered Activity of a Heterogeneous Palladium Catalyst in Oxidative Carbonylation Reactions. *Angew. Chem. Int. Ed.* **2020**, *59*, 10391-10395. (b) Alimohammadzadeh, R.; Osong, S. H.A.; Rafi, A.; Dahlström, C.; Córdova, A. Cellulosic Materials: Sustainable Surface Engineering of Lignocellulose and Cellulose by Synergistic Combination of Metal-Free Catalysis and Polyelectrolyte Complexes *Global Challenges* **2019**, *3,* 1900018.

[2] Rafi, A. A.; Ibrahem, I.; Córdova, A. Copper nanoparticles on controlled pore glass (CPG) as highly efficient heterogeneous catalysts for “click reactions”. *Sci. Rep.* **2020**, *10*, 20547.

[3] Li, M.-B.; Grape, E. S.; Bäckvall, J.-E. Palladium-Catalyzed Stereospecific Oxidative Cascade Reaction of Allenes for the Construction of Pyrrole Rings: Control of Reactivity and Selectivity. *ACS Catal.* **2019**, *9*, 5184-5190.

NMR spectra

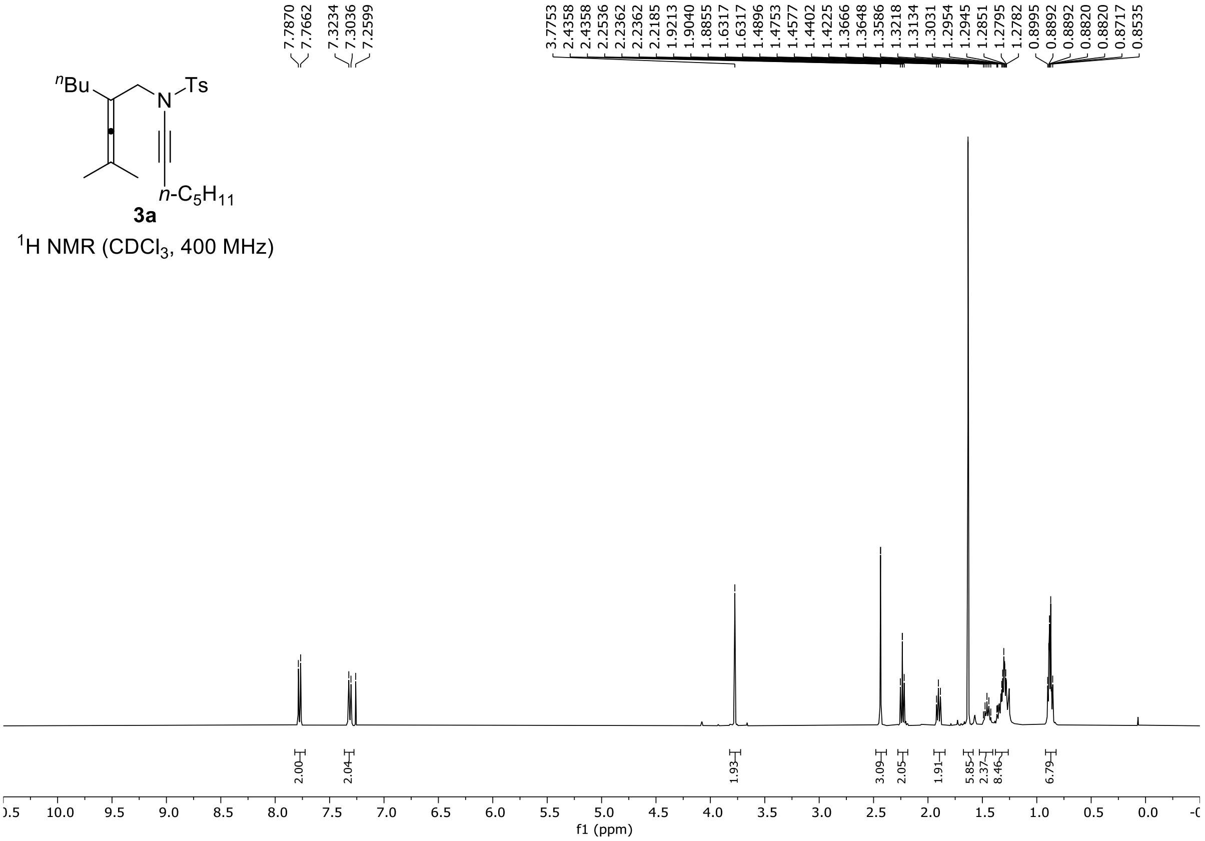

ⁿBu
N
Ts
n-C5H11
3a
1H NMR (CDCl3, 400 MHz)
7.7870
7.7662
7.3234
7.3036
7.2599
3.7753
2.4358
2.4358
2.2536
2.2362
2.2362
2.2185
1.9213
1.9040
1.8855
1.6317
1.6317
1.4896
1.4753
1.4577
1.4402
1.4225
1.3666
1.3648
1.3586
1.3218
1.3134
1.3031
1.2954
1.2945
1.2851
1.2795
1.2782
0.8995
0.8892
0.8892
0.8820
0.8820
0.8717
0.8535
2.00
2.04
1.93
3.09
2.05
1.91
5.85
2.37
8.46
6.79
f1 (ppm)

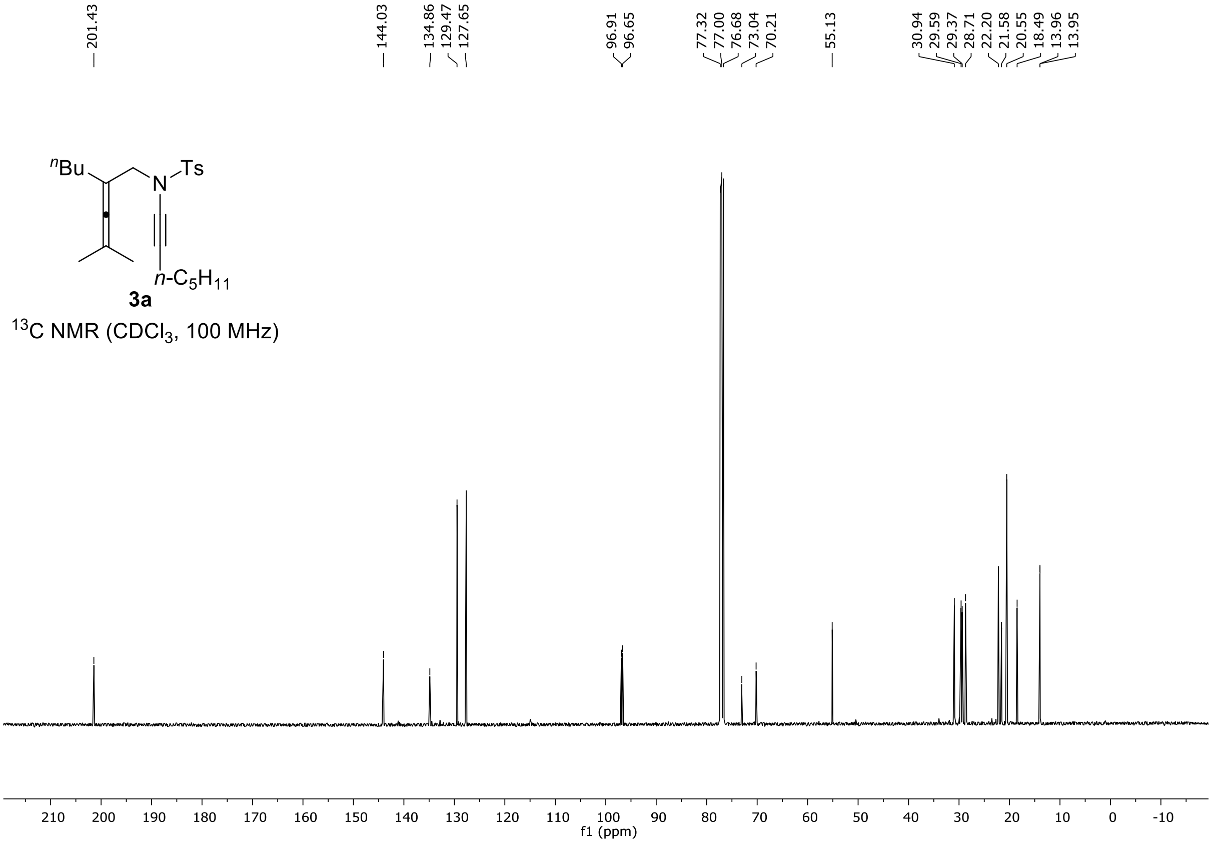

201.43
144.03
134.86
129.47
127.65
96.91
96.65
77.32
77.00
76.68
73.04
70.21
55.13
30.94
29.59
29.37
28.71
22.20
21.58
20.55
18.49
13.96
13.95
ⁿBu
N
Ts
n-C5H11
3a
13C NMR (CDCl3, 100 MHz)
f1 (ppm)

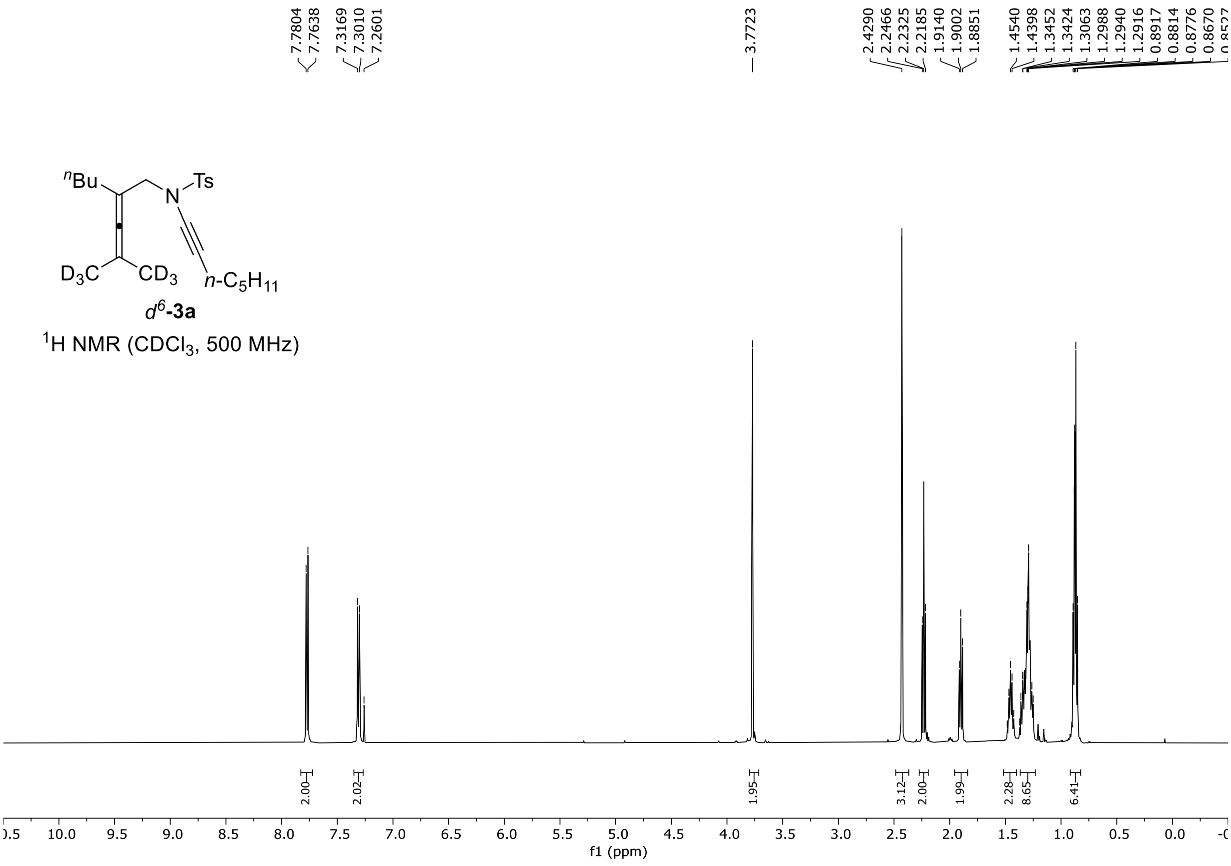

d^6-**3a**

^{1}H NMR ($CDCl_3$, 500 MHz)

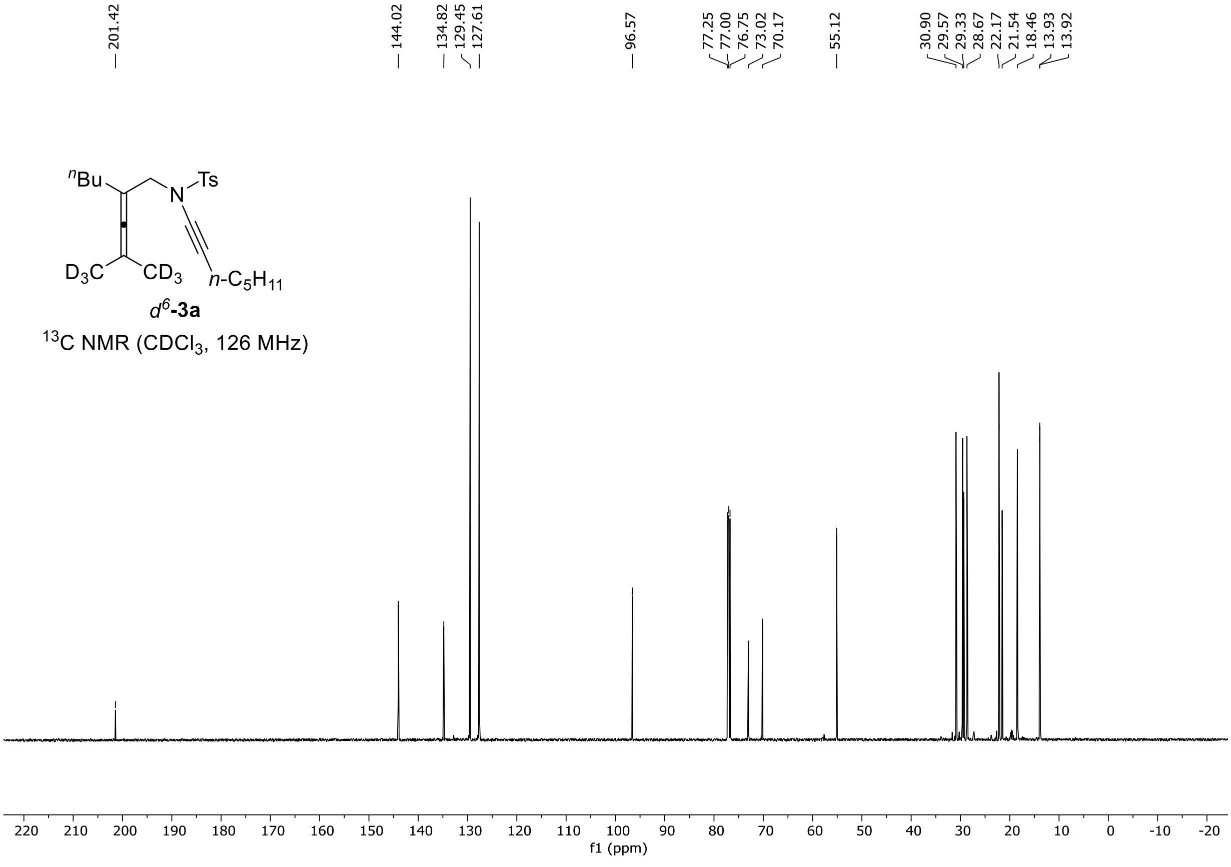

d^6-**3a**

^{13}C NMR ($CDCl_3$, 126 MHz)

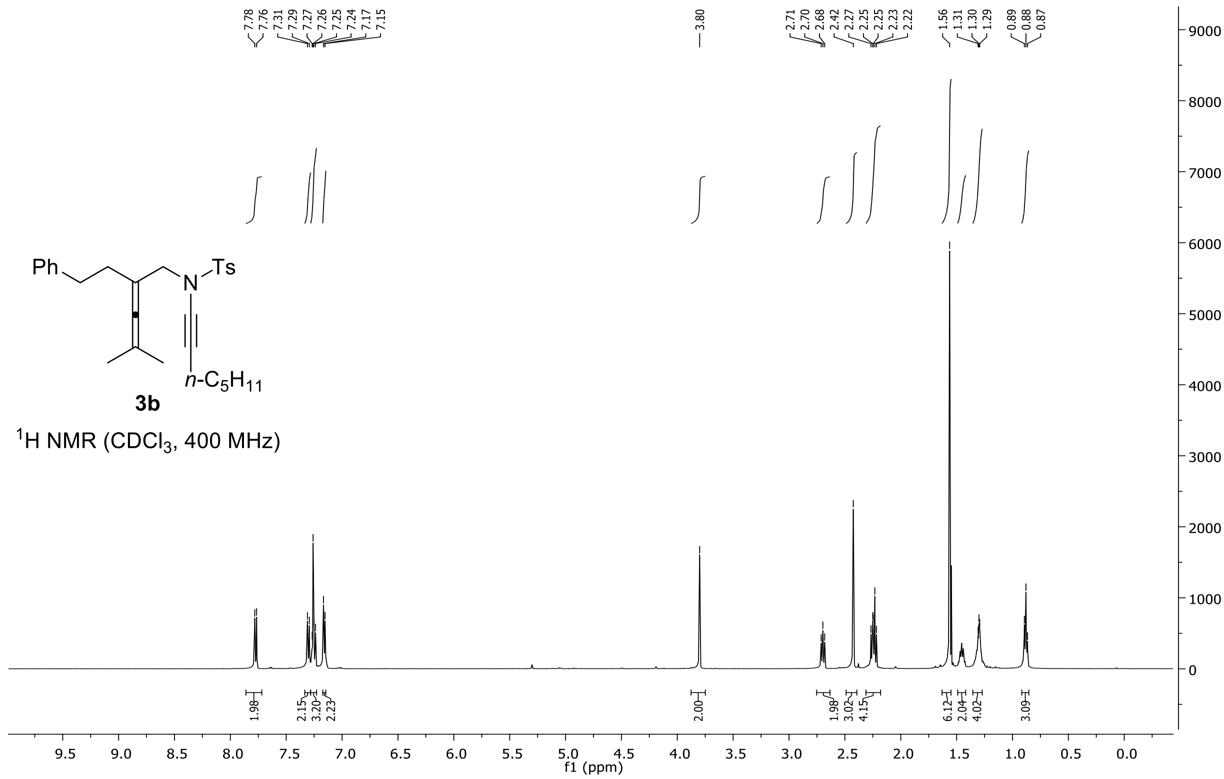
7.78
7.76
7.31
7.29
7.27
7.26
7.25
7.24
7.17
7.15
3.80
2.71
2.70
2.68
2.42
2.27
2.25
2.25
2.23
2.22
1.56
1.31
1.30
1.29
0.89
0.88
0.87
Ph
N
Ts
n-C5H11
3b
1H NMR (CDCl3, 400 MHz)
1.98
2.15
3.20
2.23
2.00
1.98
3.02
4.15
6.12
2.04
4.02
3.09
f1 (ppm)

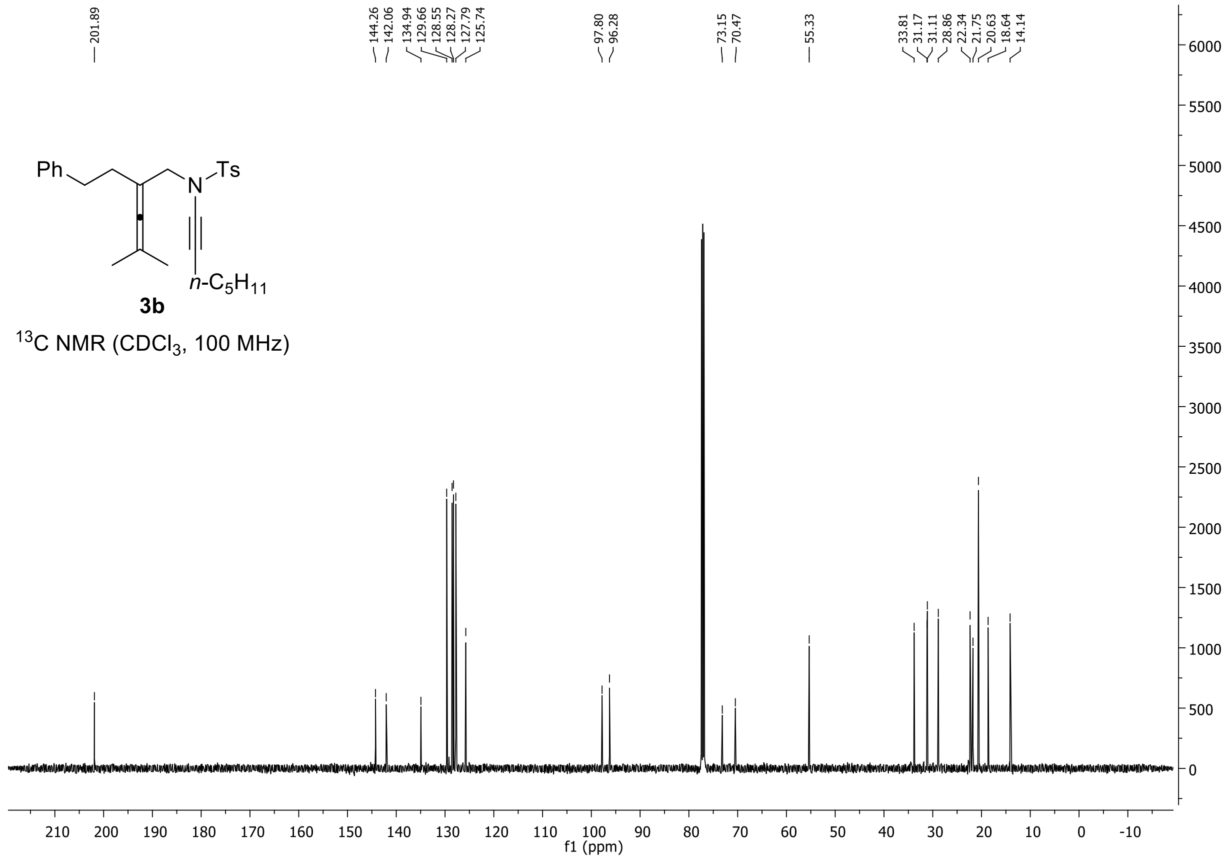
201.89
144.26
142.06
134.94
129.66
128.55
128.27
127.79
125.74
97.80
96.28
73.15
70.47
55.33
33.81
31.17
31.11
28.86
22.34
21.75
20.63
18.64
14.14
Ph
N
Ts
n-C5H11
3b
13C NMR (CDCl3, 100 MHz)
f1 (ppm)

3c

^{1}H NMR ($CDCl_3$, 400 MHz)

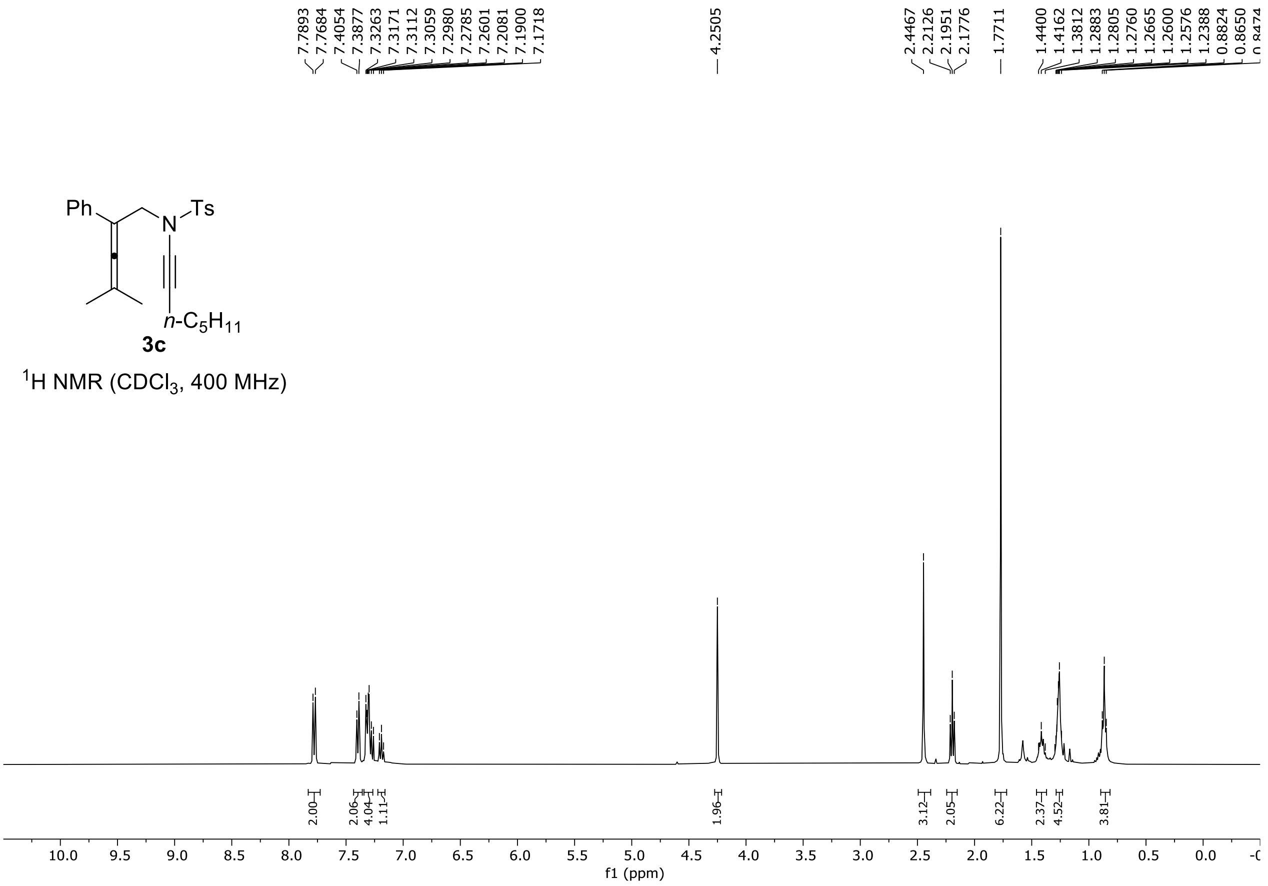

3c

^{13}C NMR ($CDCl_3$, 100 MHz)

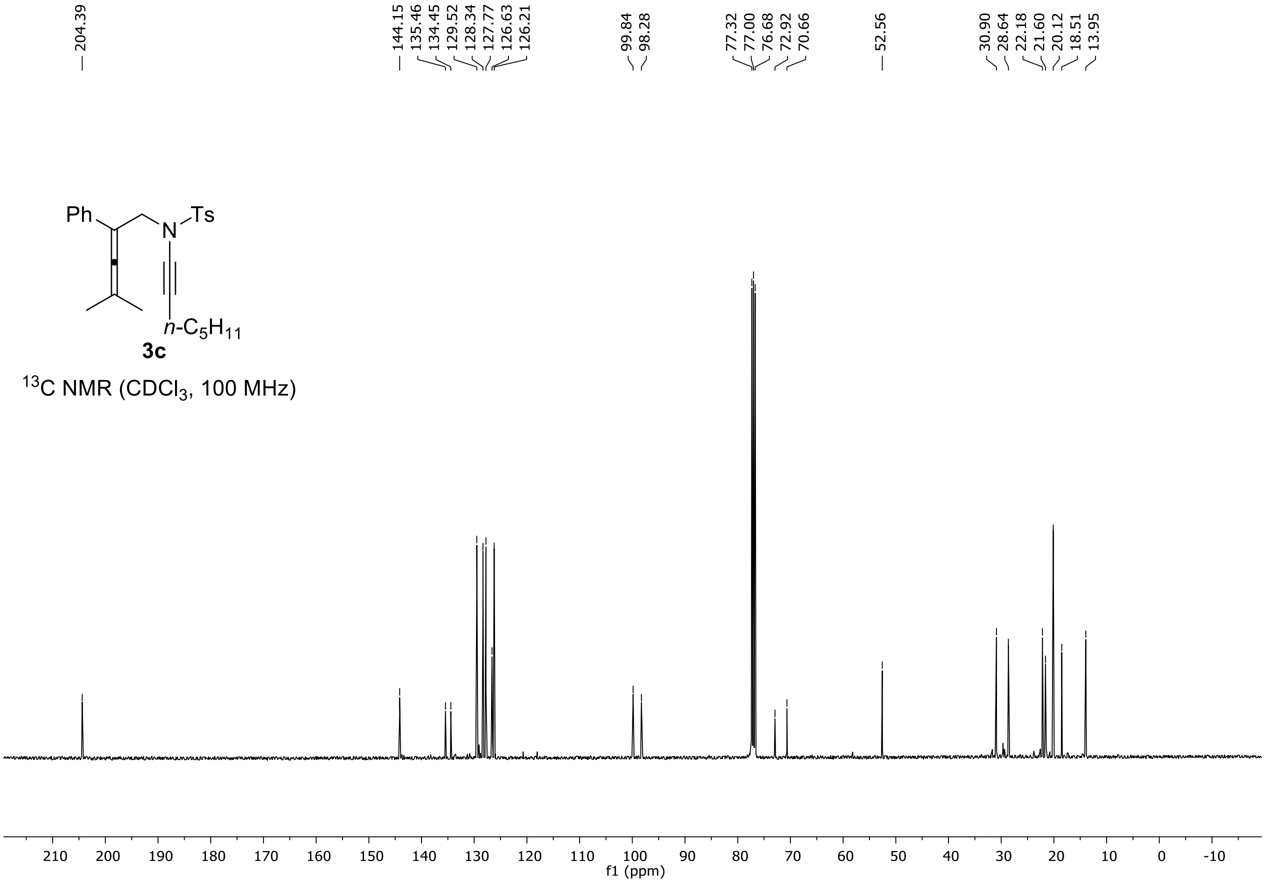

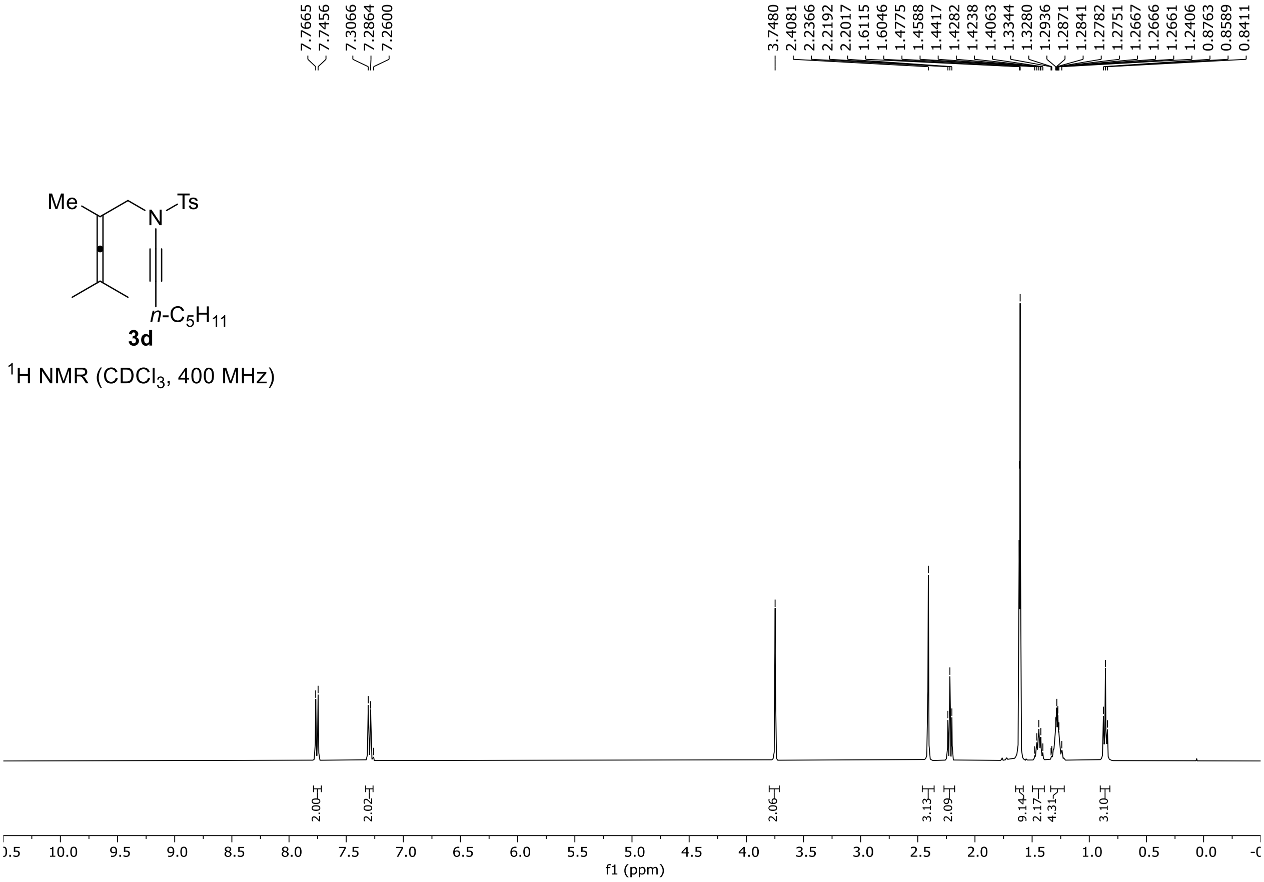
7.7665
7.7456
7.3066
7.2864
7.2600
3.7480
2.4081
2.2366
2.2192
2.2017
1.6115
1.6046
1.4775
1.4588
1.4417
1.4282
1.4238
1.4063
1.3344
1.3280
1.2936
1.2871
1.2841
1.2782
1.2751
1.2667
1.2666
1.2661
1.2406
0.8763
0.8589
0.8411
Me
N
Ts
n-C5H11
3d
1H NMR (CDCl3, 400 MHz)
2.00
2.02
2.06
3.13
2.09
9.14
2.17
4.31
3.10
f1 (ppm)

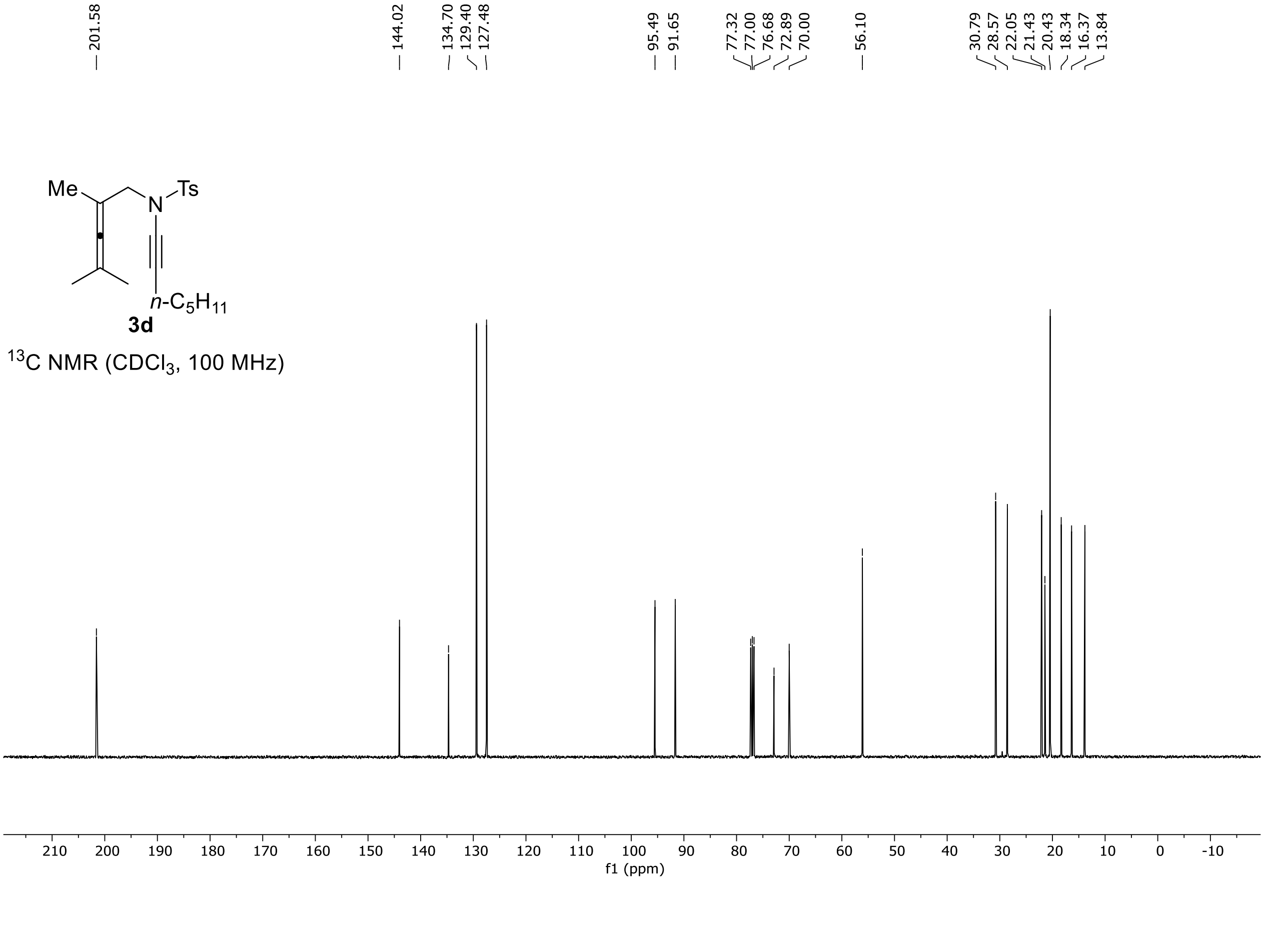
201.58
144.02
134.70
129.40
127.48
95.49
91.65
77.32
77.00
76.68
72.89
70.00
56.10
30.79
28.57
22.05
21.43
20.43
18.34
16.37
13.84
Me
N
Ts
n-C5H11
3d
13C NMR (CDCl3, 100 MHz)
f1 (ppm)

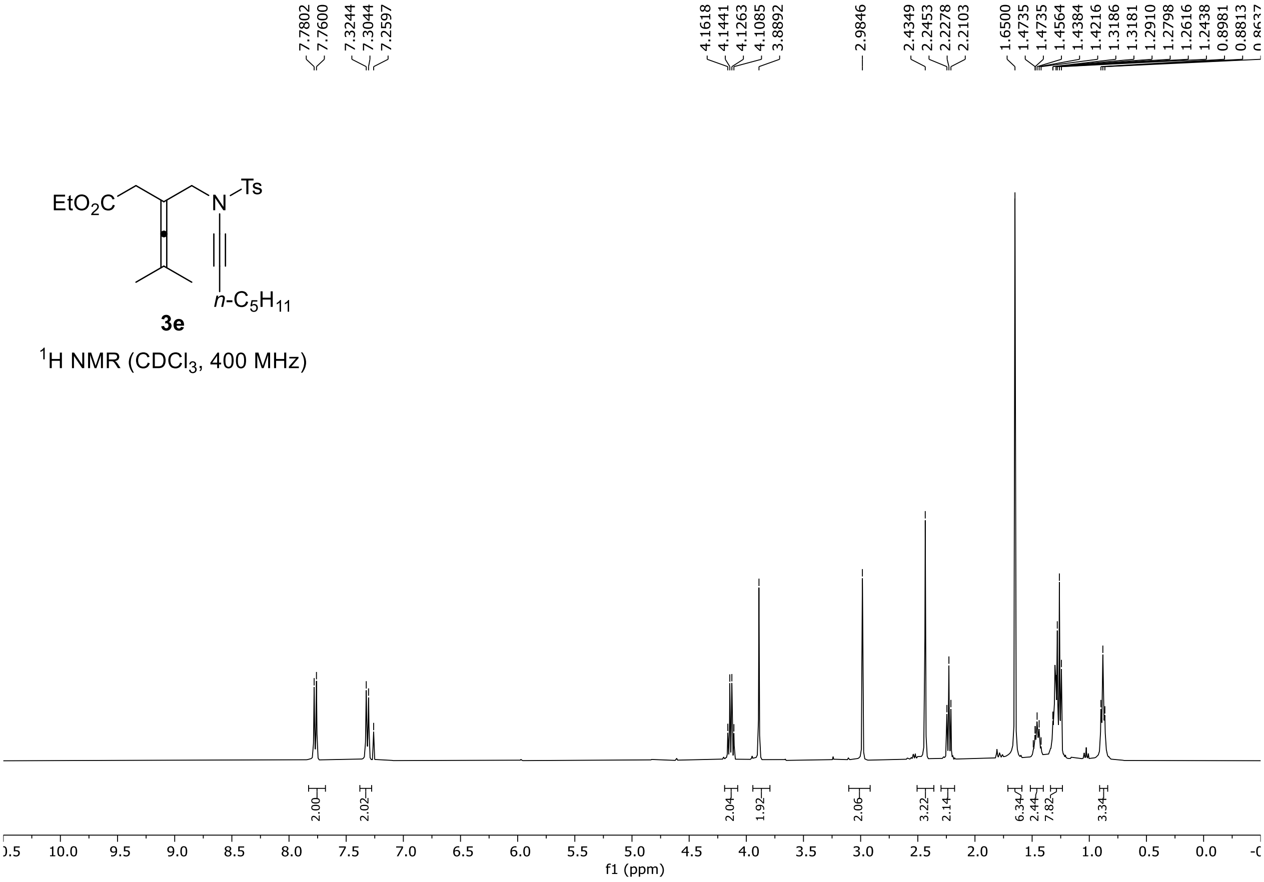
7.7802
7.7600
7.3244
7.3044
7.2597
4.1618
4.1441
4.1263
4.1085
3.8892
2.9846
2.4349
2.2453
2.2278
2.2103
1.6500
1.4735
1.4735
1.4564
1.4384
1.4216
1.3186
1.3181
1.2910
1.2798
1.2616
1.2438
0.8981
0.8813
EtO2C
N
Ts
n-C5H11
3e
1H NMR (CDCl3, 400 MHz)
f1 (ppm)

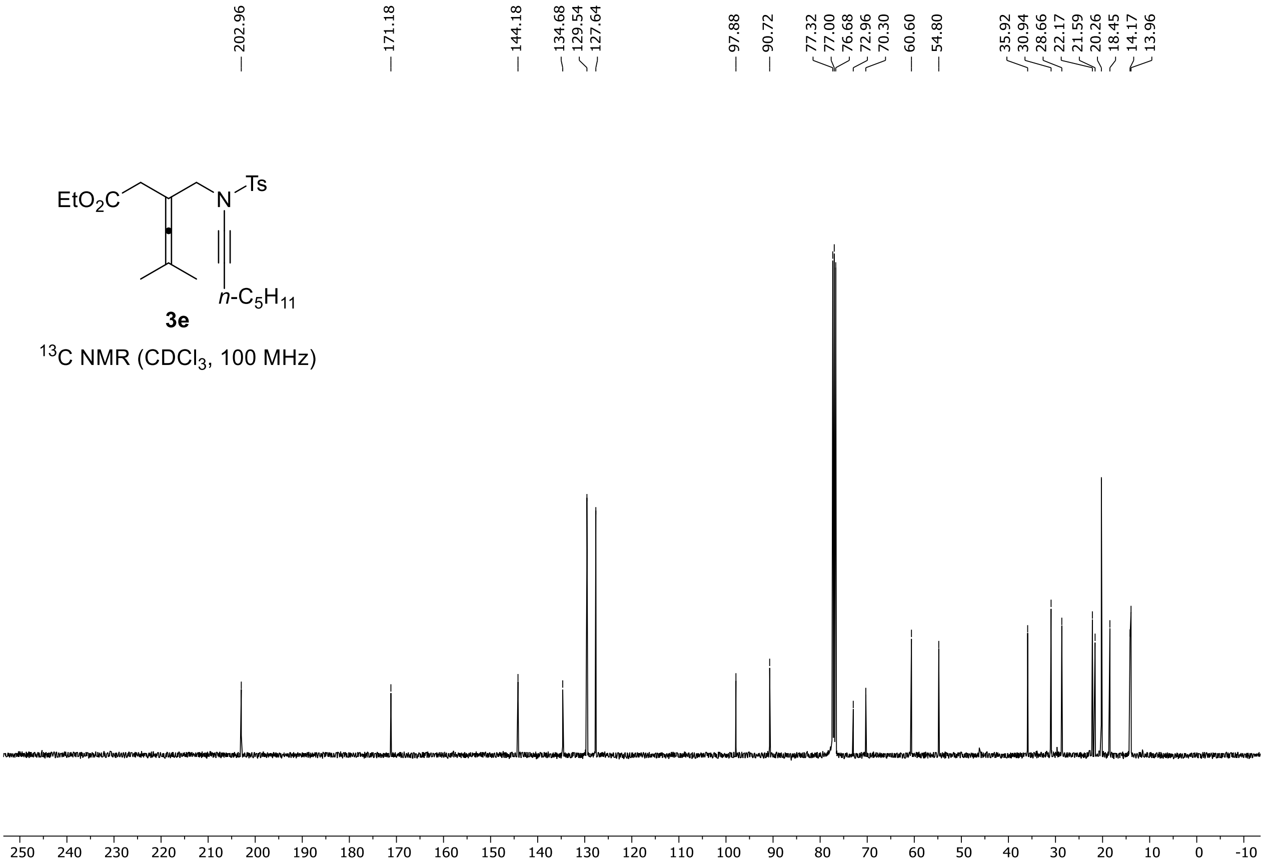
202.96
171.18
144.18
134.68
129.54
127.64
97.88
90.72
77.32
77.00
76.68
72.96
70.30
60.60
54.80
35.92
30.94
28.66
22.17
21.59
20.26
18.45
14.17
13.96
EtO2C
N
Ts
n-C5H11
3e
13C NMR (CDCl3, 100 MHz)
f1 (ppm)

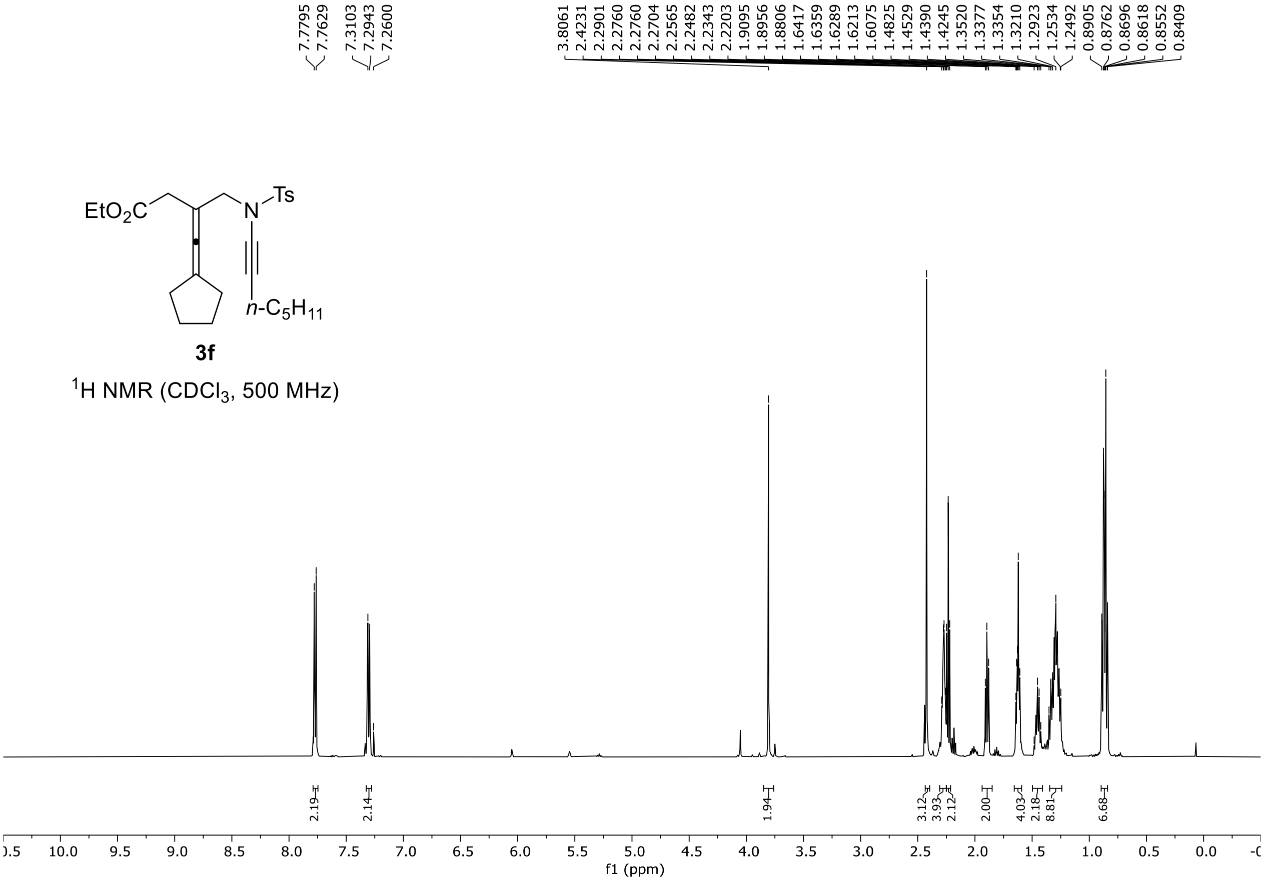
7.7795
7.7629
7.3103
7.2943
7.2600
3.8061
2.4231
2.2901
2.2760
2.2760
2.2704
2.2565
2.2482
2.2343
2.2203
1.9095
1.8956
1.8806
1.6417
1.6359
1.6289
1.6213
1.6075
1.4825
1.4529
1.4390
1.4245
1.3520
1.3377
1.3354
1.3210
1.2923
1.2534
1.2492
0.8905
0.8762
0.8696
0.8618
0.8552
0.8409
EtO2C
N
Ts
n-C5H11
3f
1H NMR (CDCl3, 500 MHz)
2.19
2.14
1.94
3.12
3.93
2.12
2.00
4.03
2.18
8.81
6.68
f1 (ppm)

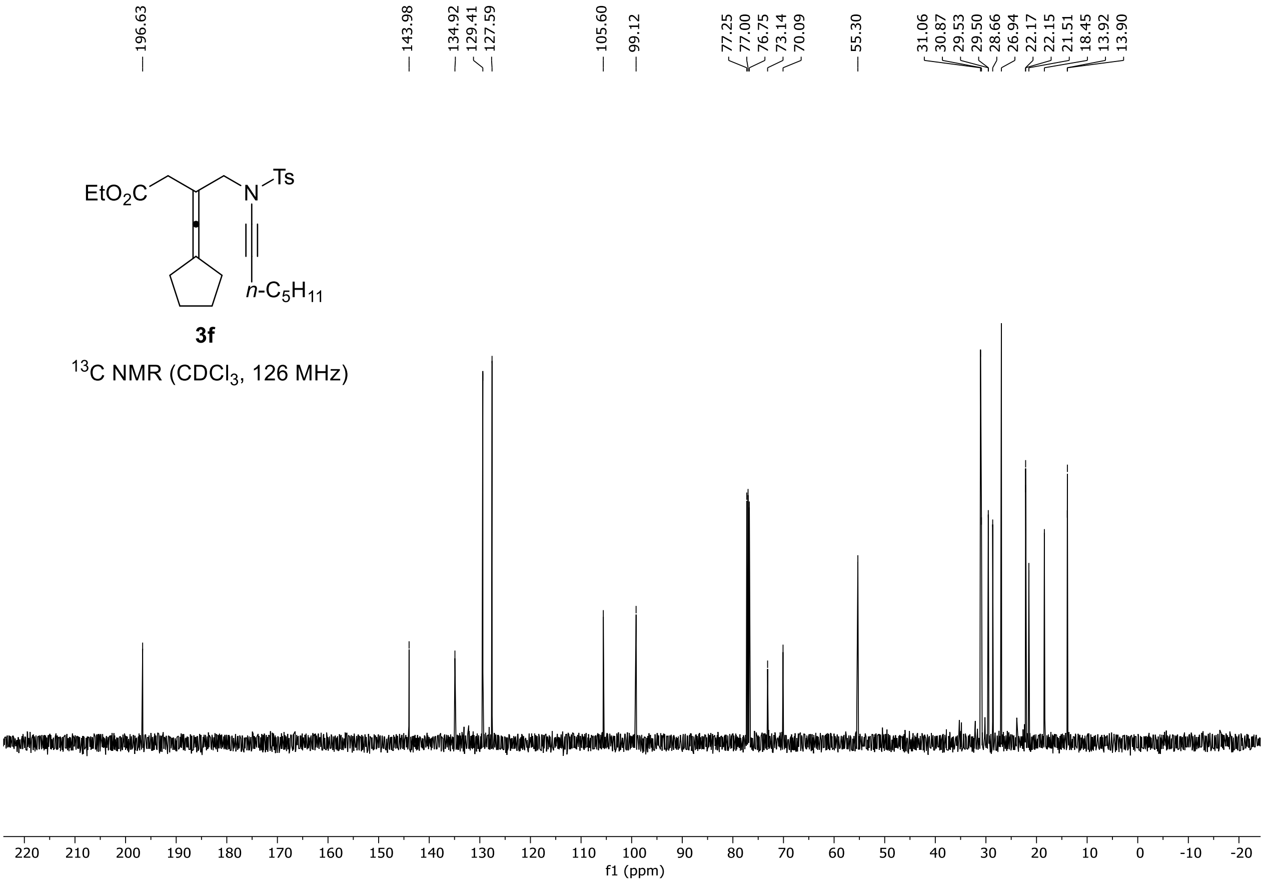
196.63
143.98
134.92
129.41
127.59
105.60
99.12
77.25
77.00
76.75
73.14
70.09
55.30
31.06
30.87
29.53
29.50
28.66
26.94
22.17
22.15
21.51
18.45
13.92
13.90
EtO2C
N
Ts
n-C5H11
3f
13C NMR (CDCl3, 126 MHz)
f1 (ppm)

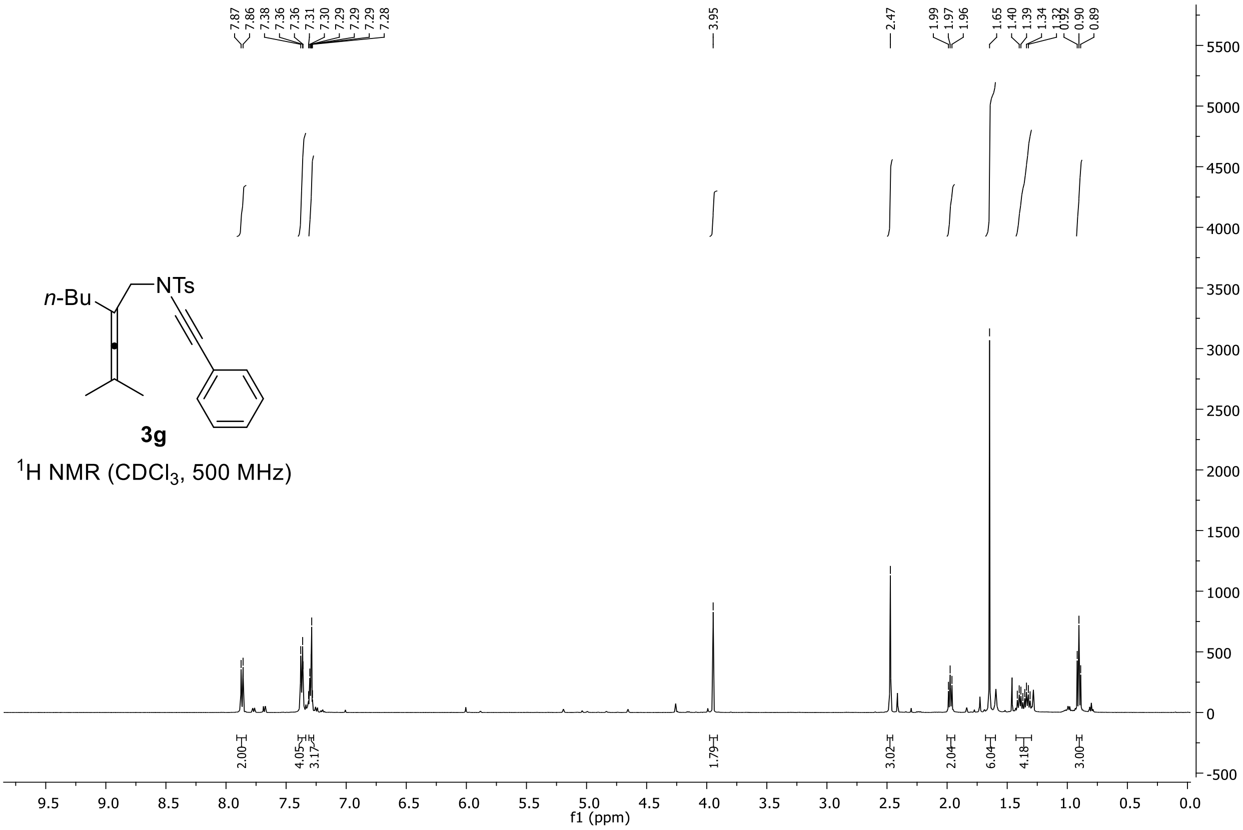

3g

^1H NMR ($CDCl_3$, 500 MHz)

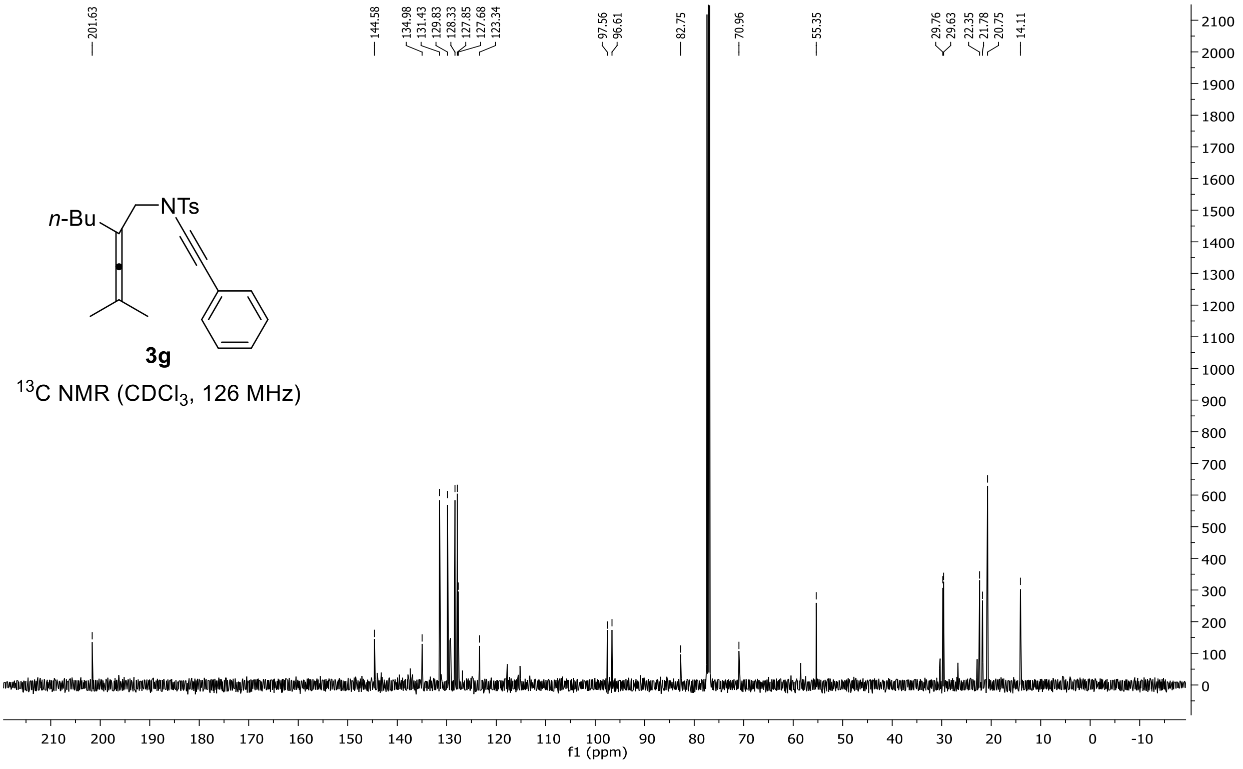

3g

^{13}C NMR ($CDCl_3$, 126 MHz)

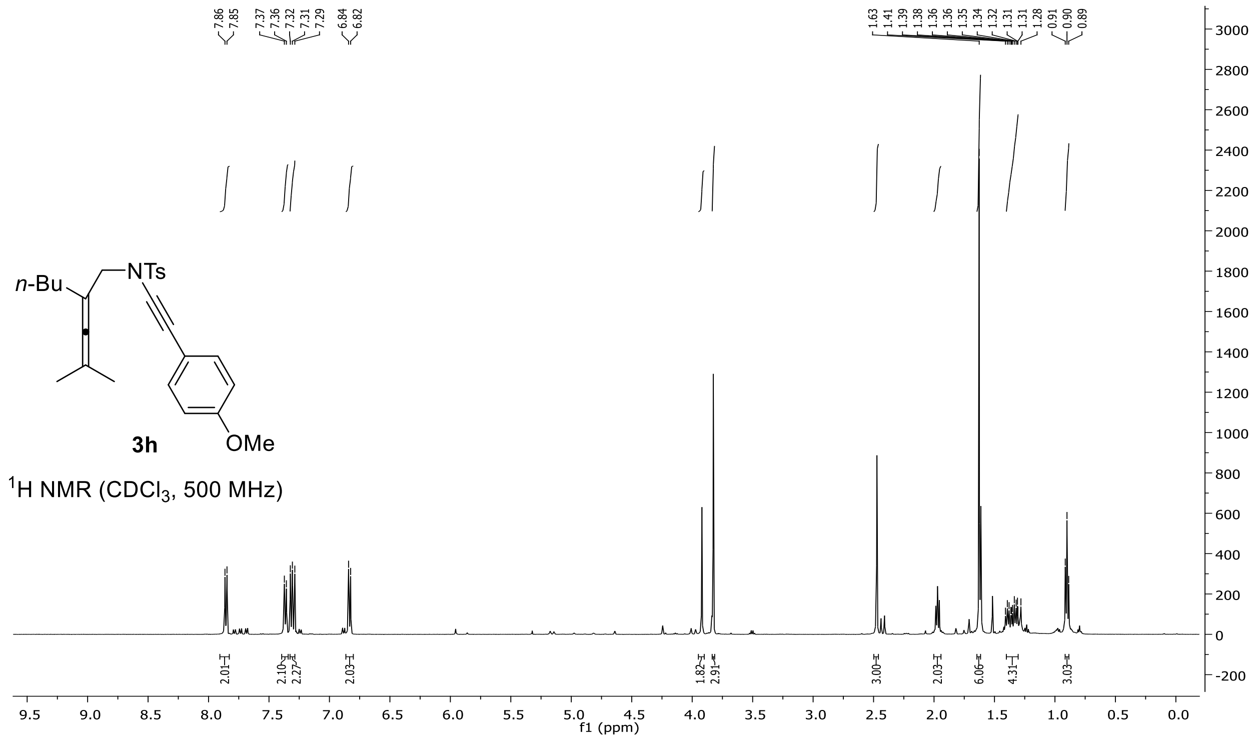
7.86
7.85
7.37
7.36
7.32
7.31
7.29
6.84
6.82
1.63
1.41
1.39
1.38
1.36
1.36
1.35
1.34
1.32
1.31
1.31
1.28
0.91
0.90
0.89
n-Bu
NTs
3h
OMe
^{1}H NMR ($CDCl_3$, 500 MHz)
2.01
2.10
2.27
2.03
1.82
2.91
3.00
2.03
6.06
4.31
3.03
f1 (ppm)

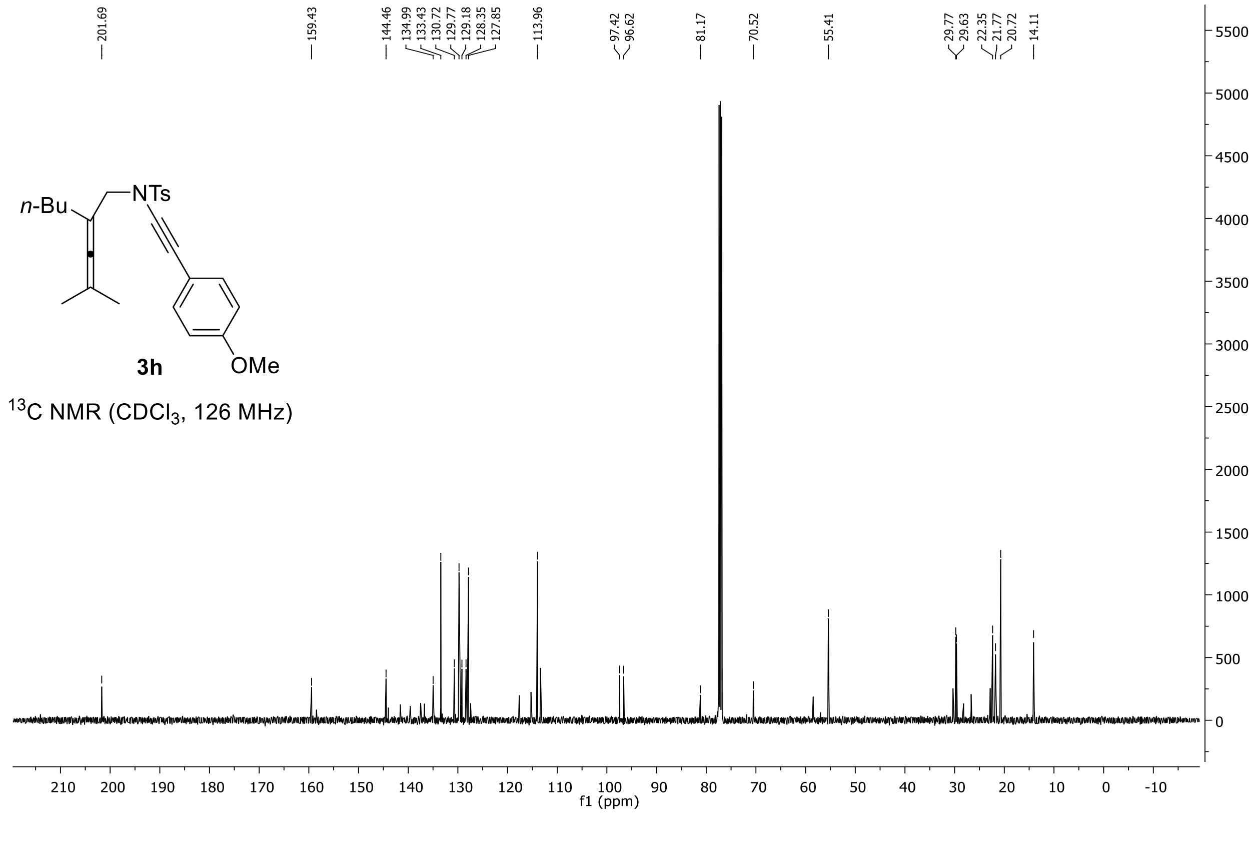
201.69
159.43
144.46
134.99
133.43
130.72
129.77
129.18
128.35
127.85
113.96
97.42
96.62
81.17
70.52
55.41
29.77
29.63
22.35
21.77
20.72
14.11
n-Bu
NTs
3h
OMe
^{13}C NMR ($CDCl_3$, 126 MHz)
f1 (ppm)

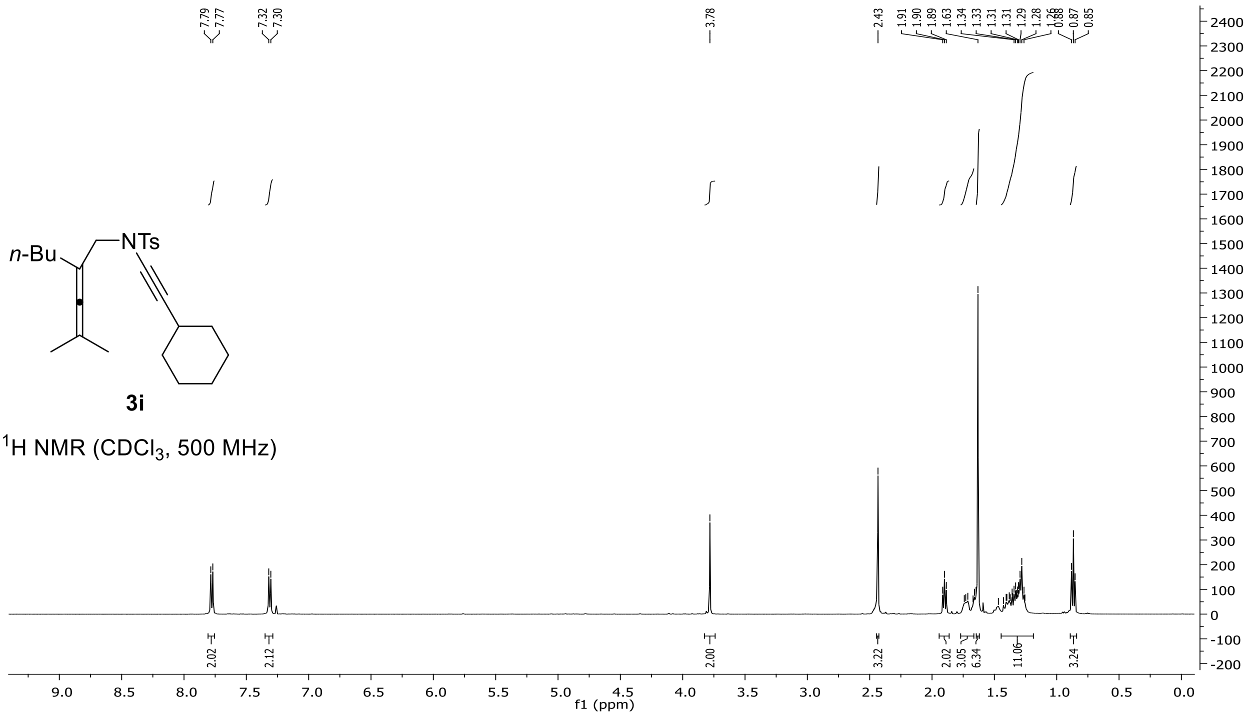

n-Bu
NTs
3i
1H NMR (CDCl3, 500 MHz)
7.79 7.77 7.32 7.30 3.78 2.43 1.91 1.90 1.89 1.63 1.34 1.33 1.31 1.31 1.29 1.28 1.26 0.88 0.87 0.85
2.02 2.12 2.00 3.22 2.02 3.05 6.34 11.06 3.24
f1 (ppm)

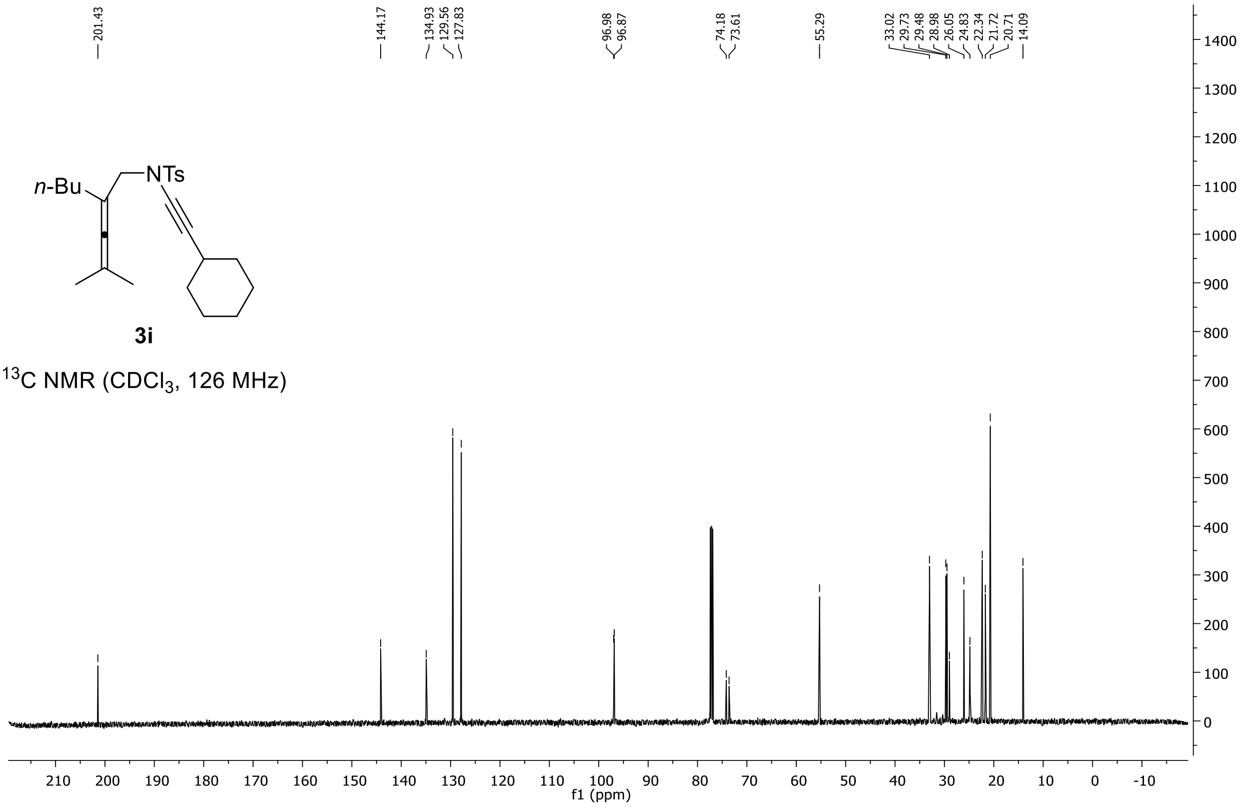

n-Bu
NTs
3i
13C NMR (CDCl3, 126 MHz)
201.43 144.17 134.93 129.56 127.83 96.98 96.87 74.18 73.61 55.29 33.02 29.73 29.48 28.98 26.05 24.83 22.34 21.72 20.71 14.09
f1 (ppm)

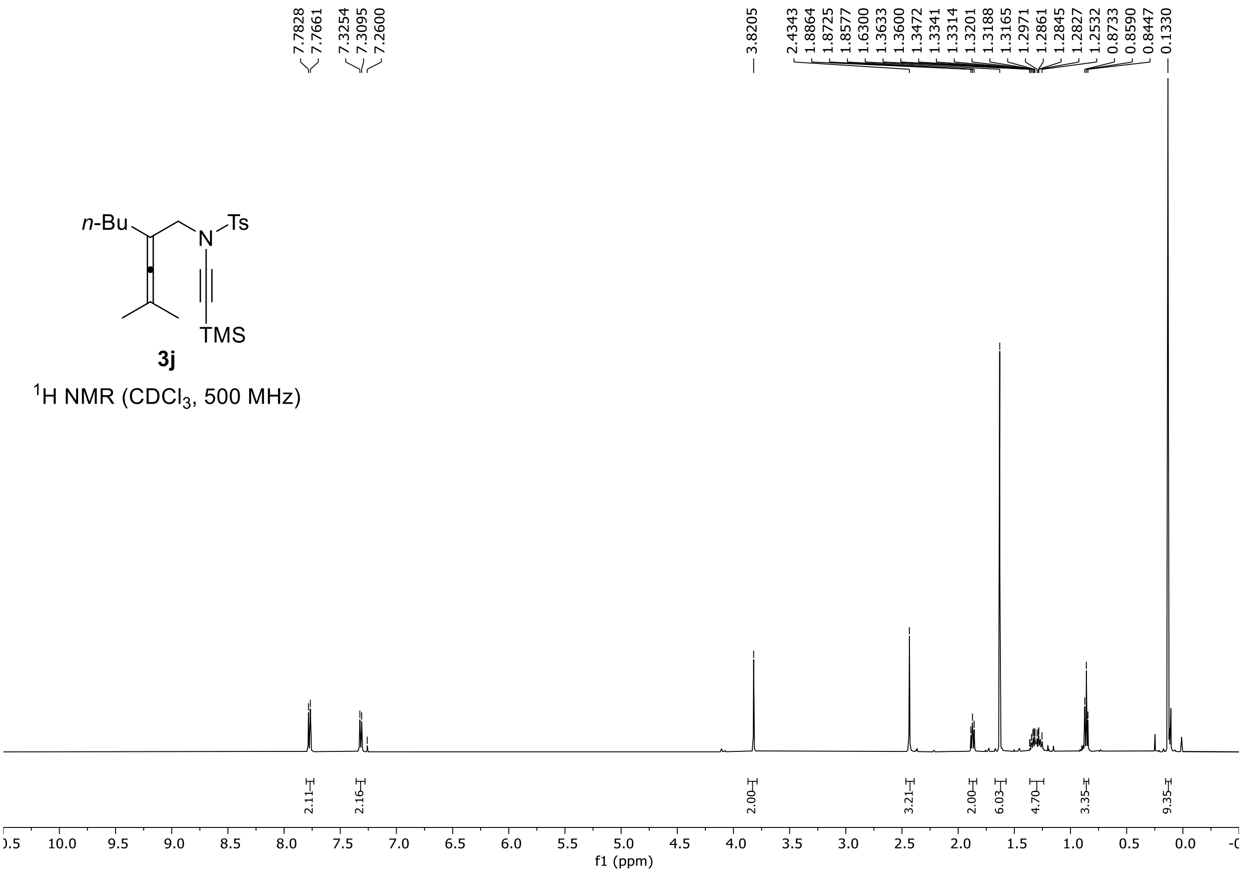
7.7828
7.7661
7.3254
7.3095
7.2600
3.8205
2.4343
1.8864
1.8725
1.8577
1.6300
1.3633
1.3600
1.3472
1.3341
1.3314
1.3201
1.3188
1.3165
1.2971
1.2861
1.2845
1.2827
1.2532
0.8733
0.8590
0.8447
0.1330
n-Bu
N
Ts
TMS
3j
¹H NMR (CDCl₃, 500 MHz)
2.11
2.16
2.00
3.21
2.00
6.03
4.70
3.35
9.35
f1 (ppm)

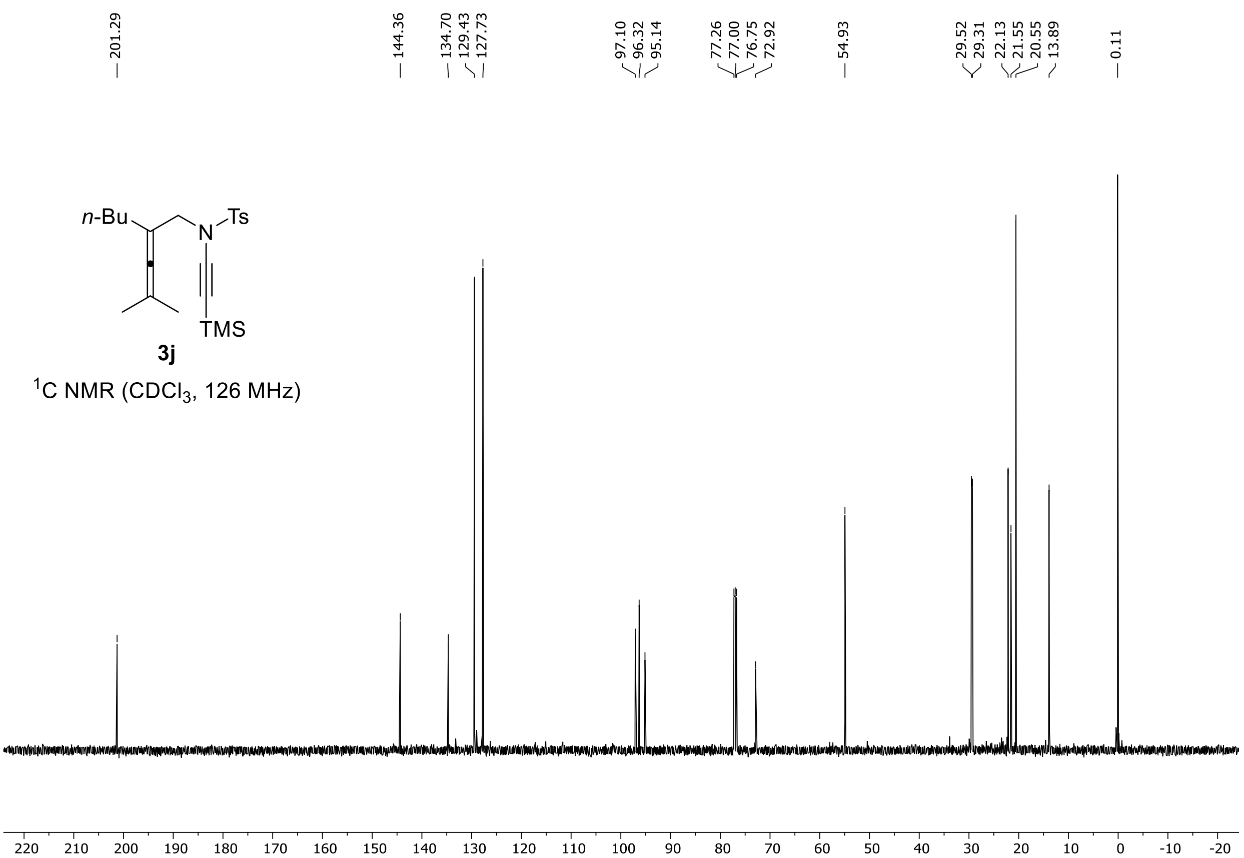
201.29
144.36
134.70
129.43
127.73
97.10
96.32
95.14
77.26
77.00
76.75
72.92
54.93
29.52
29.31
22.13
21.55
20.55
13.89
0.11
n-Bu
N
Ts
TMS
3j
¹C NMR (CDCl₃, 126 MHz)
f1 (ppm)

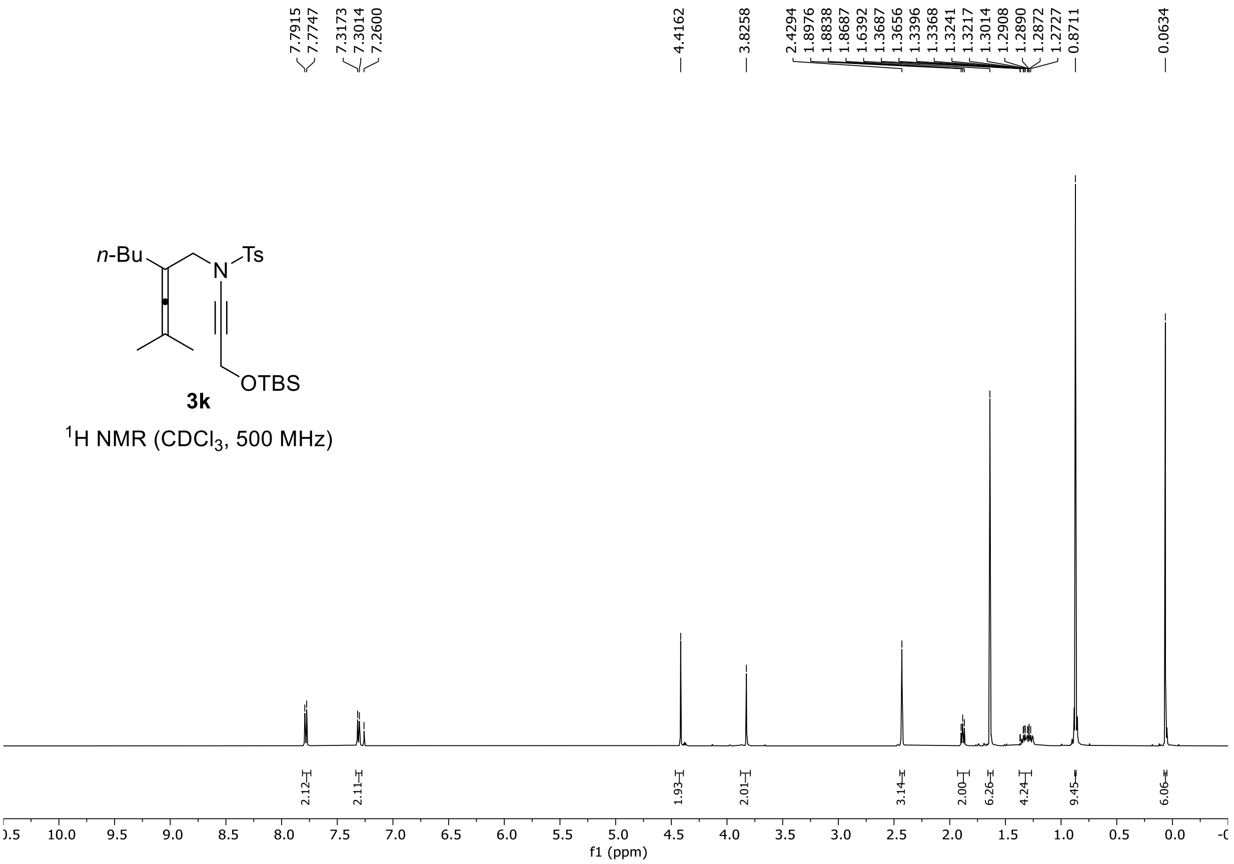
7.7915
7.7747
7.3173
7.3014
7.2600
4.4162
3.8258
2.4294
1.8976
1.8838
1.8687
1.6392
1.3687
1.3656
1.3396
1.3368
1.3241
1.3217
1.3014
1.2908
1.2890
1.2872
1.2727
0.8711
0.0634
n-Bu
N
Ts
OTBS
3k
^{1}H NMR ($CDCl_3$, 500 MHz)
2.12
2.11
1.93
2.01
3.14
2.00
6.26
4.24
9.45
6.06
f1 (ppm)

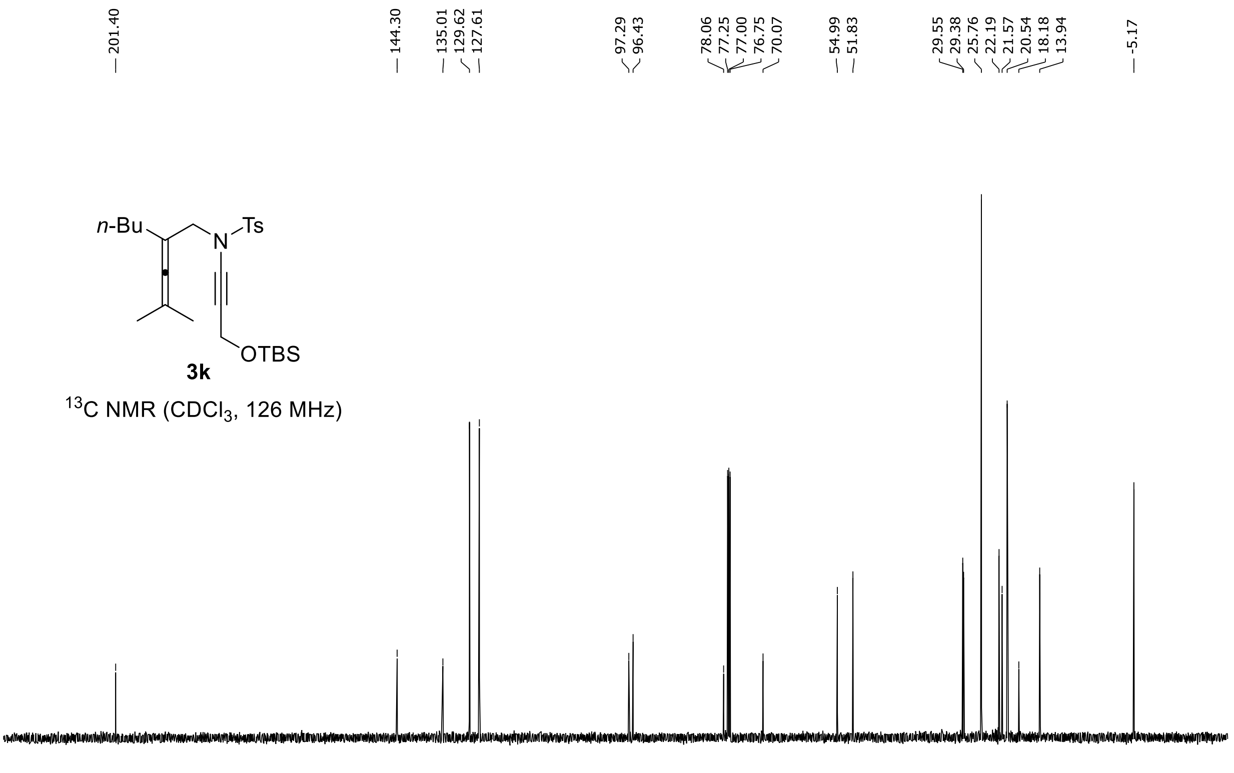
201.40
144.30
135.01
129.62
127.61
97.29
96.43
78.06
77.25
77.00
76.75
70.07
54.99
51.83
29.55
29.38
25.76
22.19
21.57
20.54
18.18
13.94
-5.17
n-Bu
N
Ts
OTBS
3k
^{13}C NMR ($CDCl_3$, 126 MHz)
f1 (ppm)

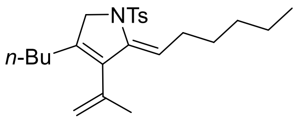

4a

^{1}H NMR ($CDCl_3$, 400 MHz)

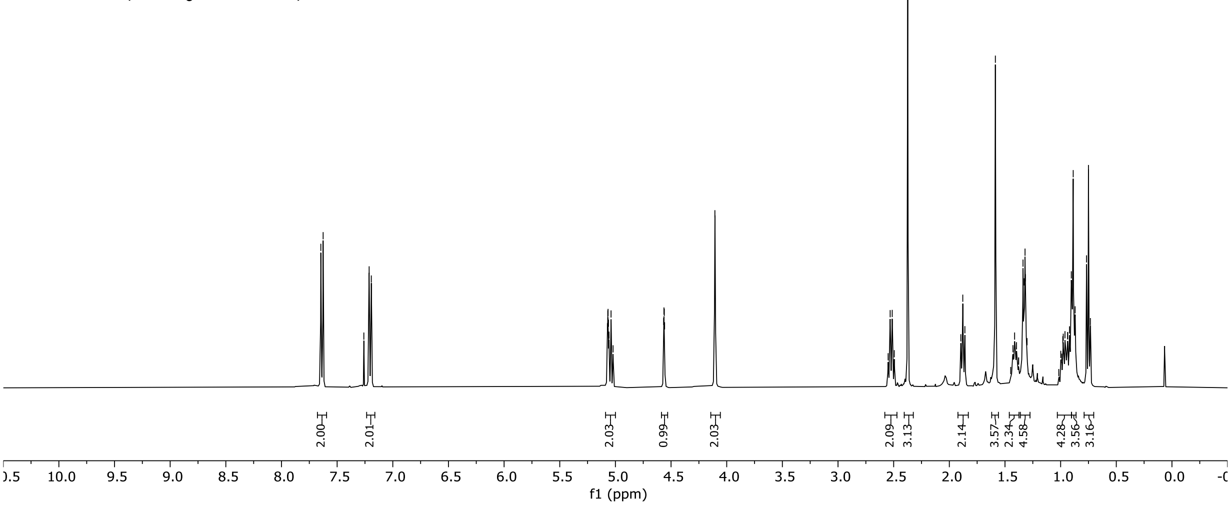

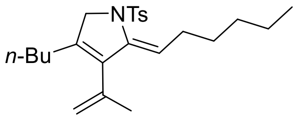

4a

^{13}C NMR ($CDCl_3$, 100 MHz)

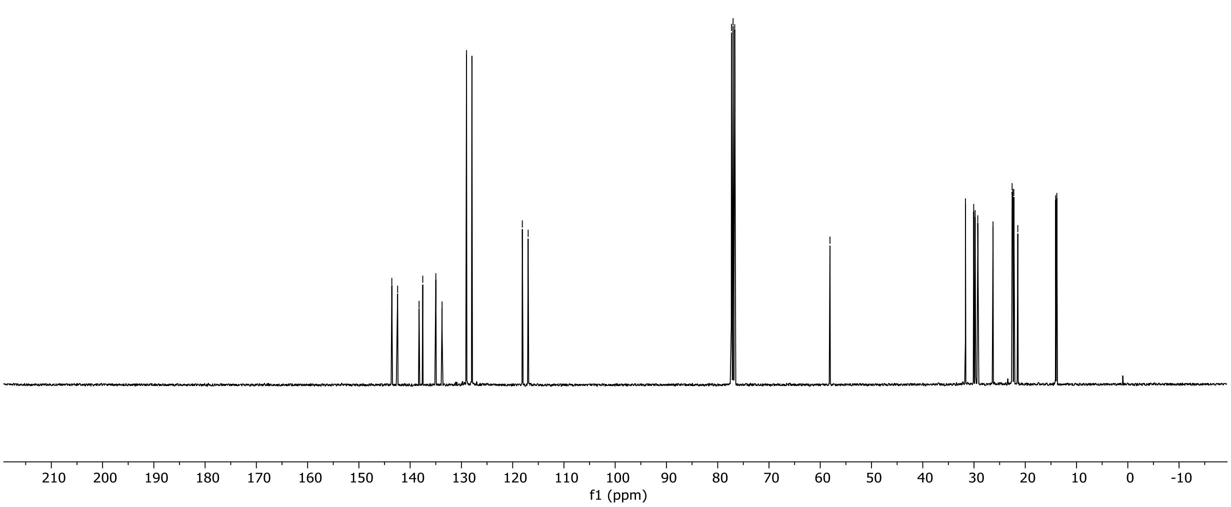

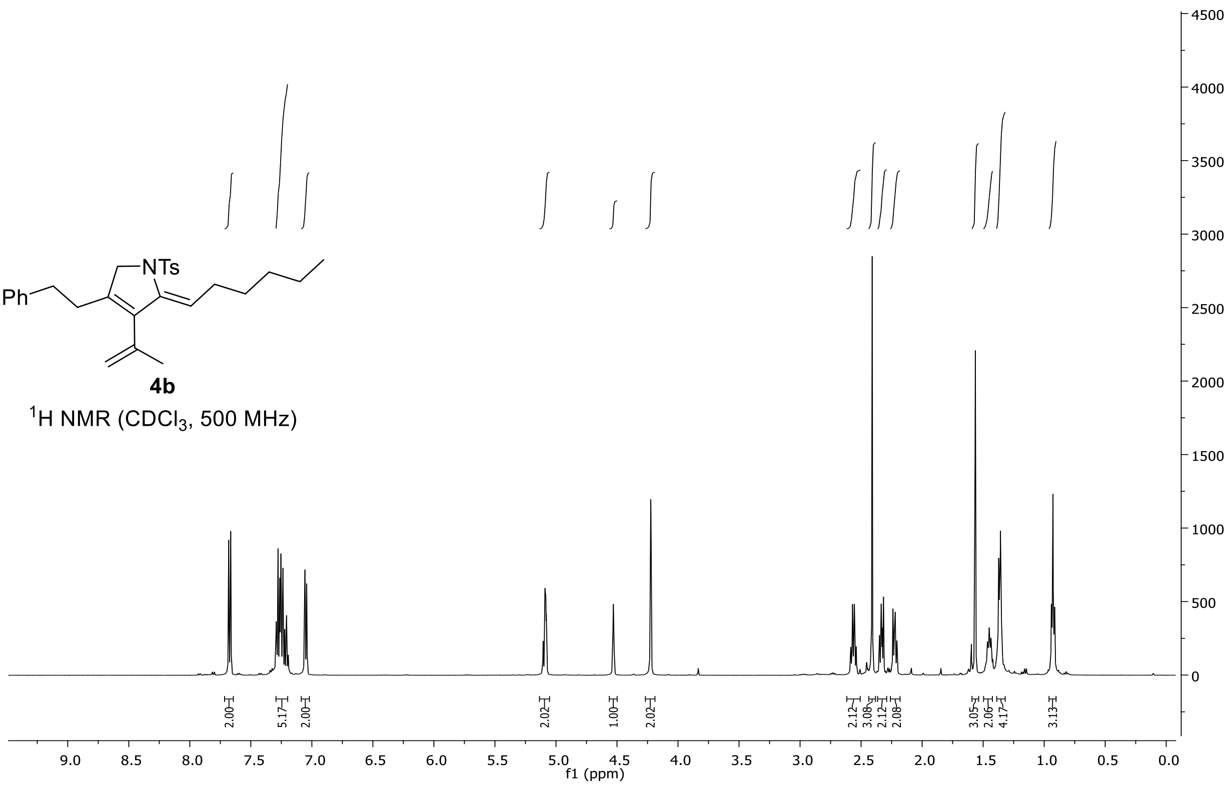

4b

^{1}H NMR ($CDCl_3$, 500 MHz)

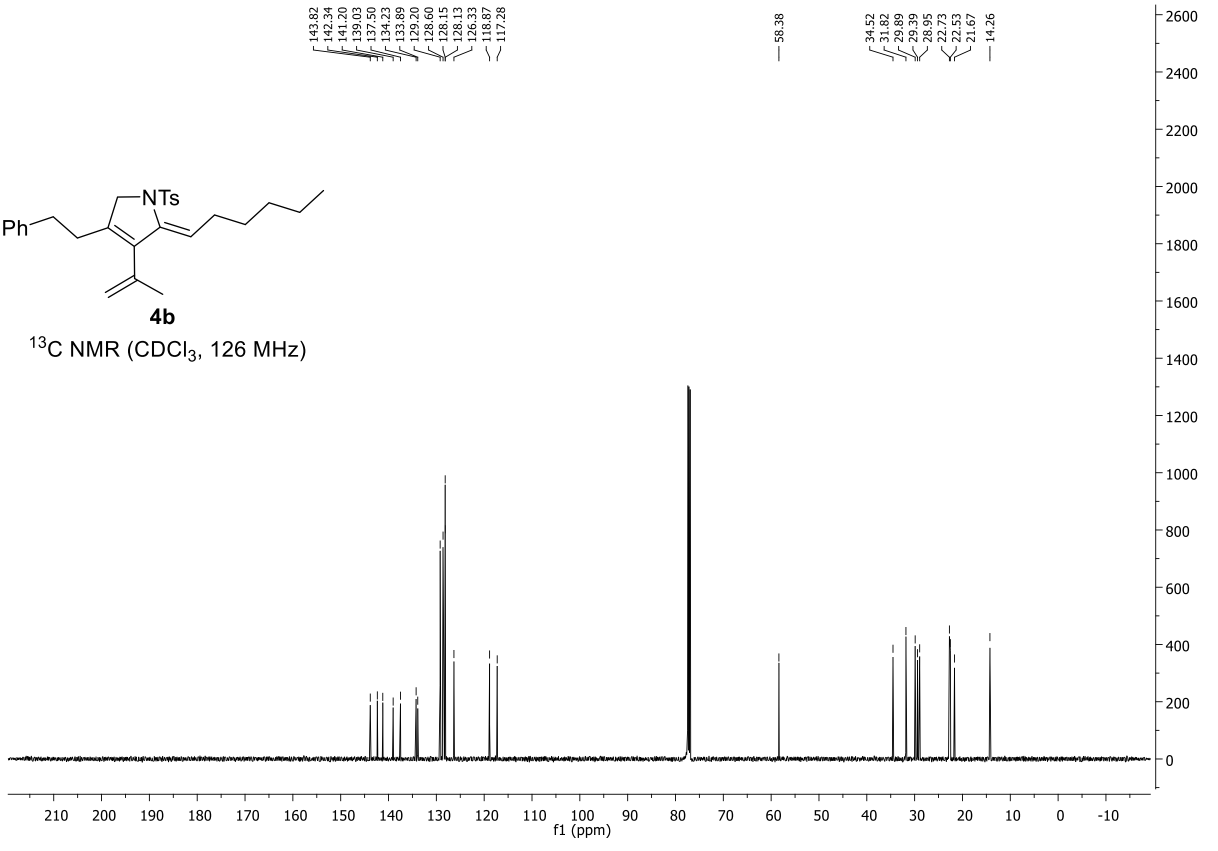

4b

^{13}C NMR ($CDCl_3$, 126 MHz)

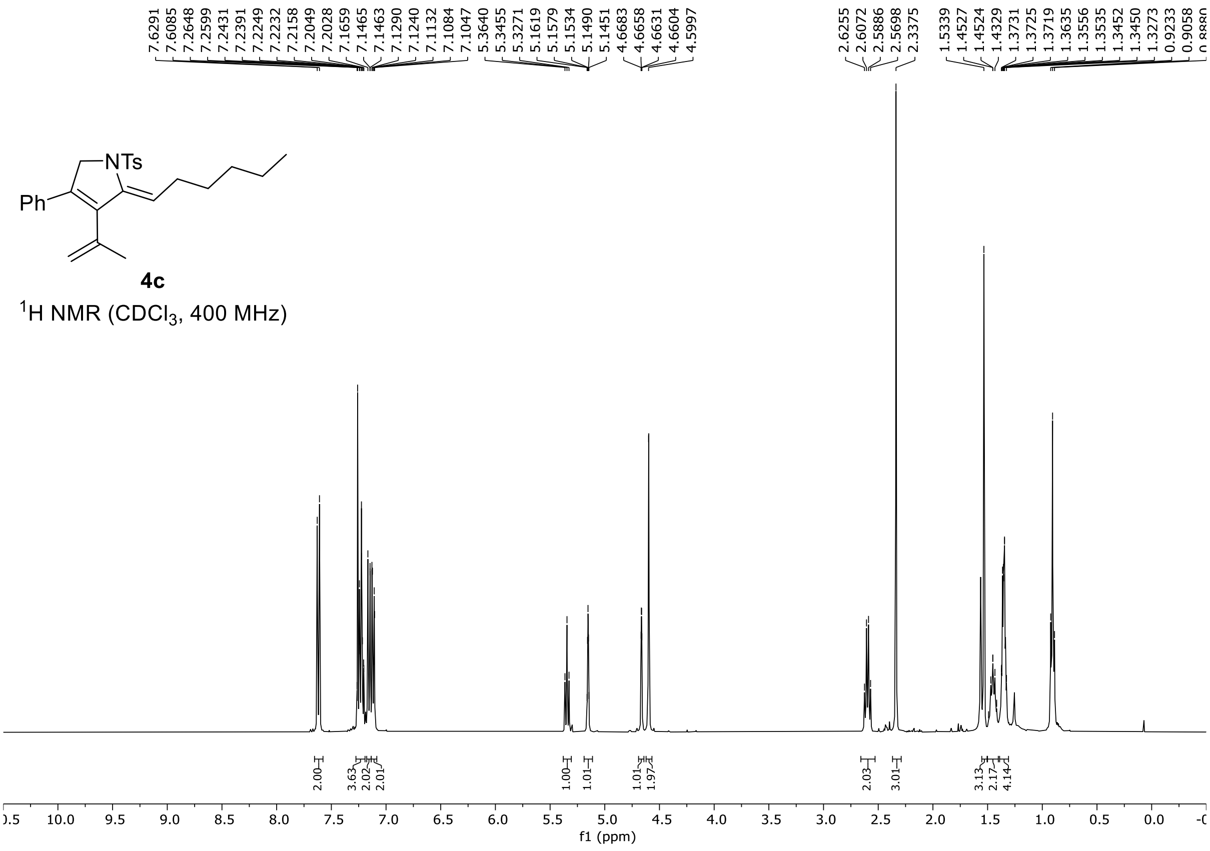
7.6291
7.6085
7.2648
7.2599
7.2431
7.2391
7.2249
7.2232
7.2158
7.2049
7.2028
7.1659
7.1465
7.1463
7.1290
7.1240
7.1132
7.1084
7.1047
5.3640
5.3455
5.3271
5.1619
5.1579
5.1534
5.1490
5.1451
4.6683
4.6658
4.6631
4.6604
4.5997
2.6255
2.6072
2.5886
2.5698
2.3375
1.5339
1.4527
1.4524
1.4329
1.3731
1.3725
1.3719
1.3635
1.3556
1.3535
1.3452
1.3450
1.3273
0.9233
0.9058
NTs
Ph
4c
¹H NMR (CDCl₃, 400 MHz)
2.00
3.63
2.02
2.01
1.00
1.01
1.01
1.97
2.03
3.01
3.13
2.17
4.14
f1 (ppm)

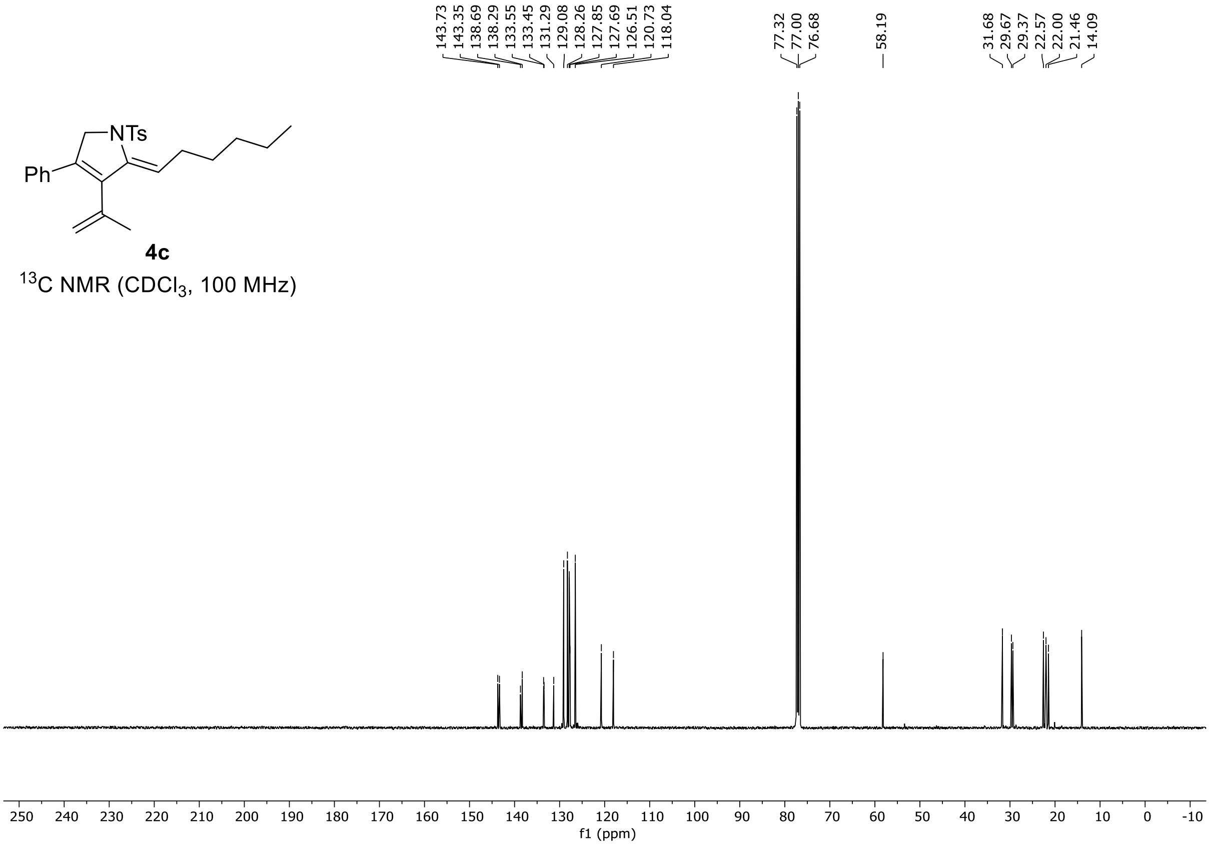
143.73
143.35
138.69
138.29
133.55
133.45
131.29
129.08
128.26
127.85
127.69
126.51
120.73
118.04
77.32
77.00
76.68
58.19
31.68
29.67
29.37
22.57
22.00
21.46
14.09
NTs
Ph
4c
¹³C NMR (CDCl₃, 100 MHz)
f1 (ppm)

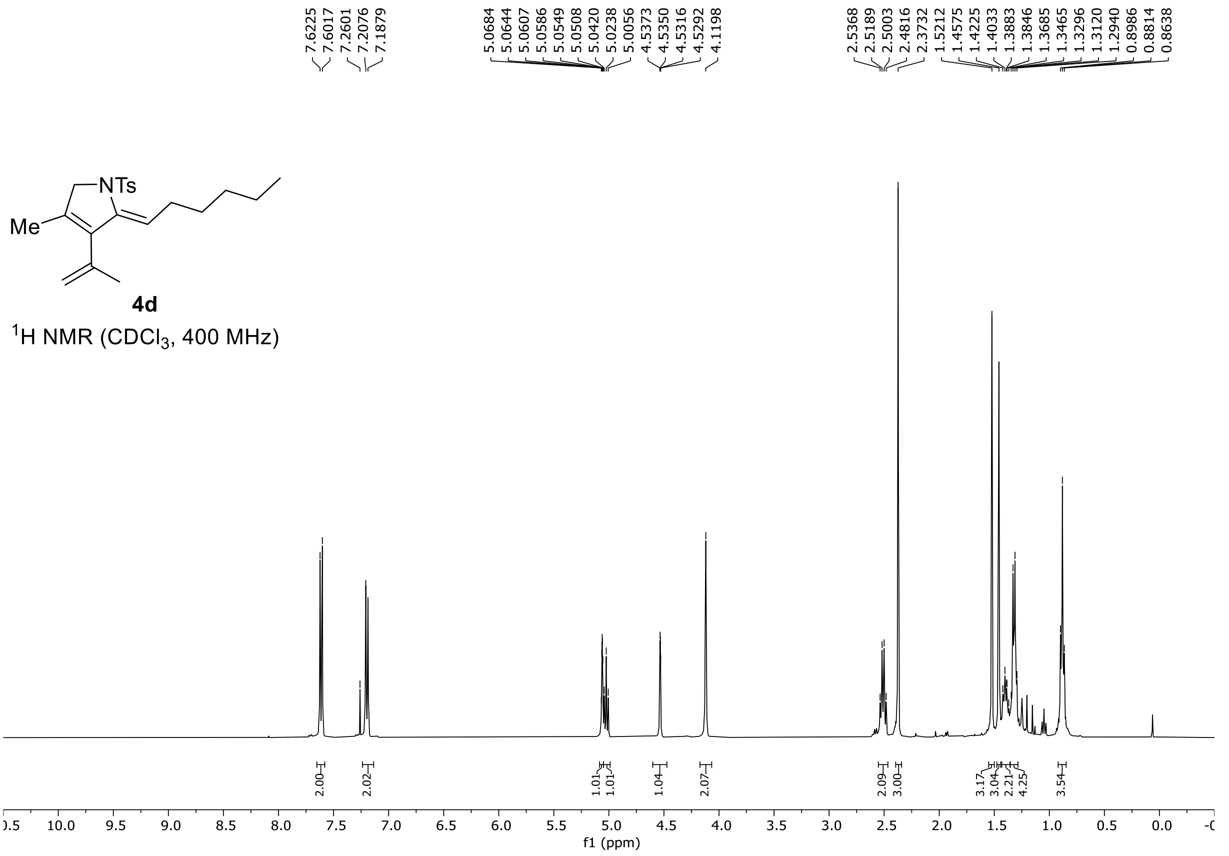

4d

^{1}H NMR (CDCl$_3$, 400 MHz)

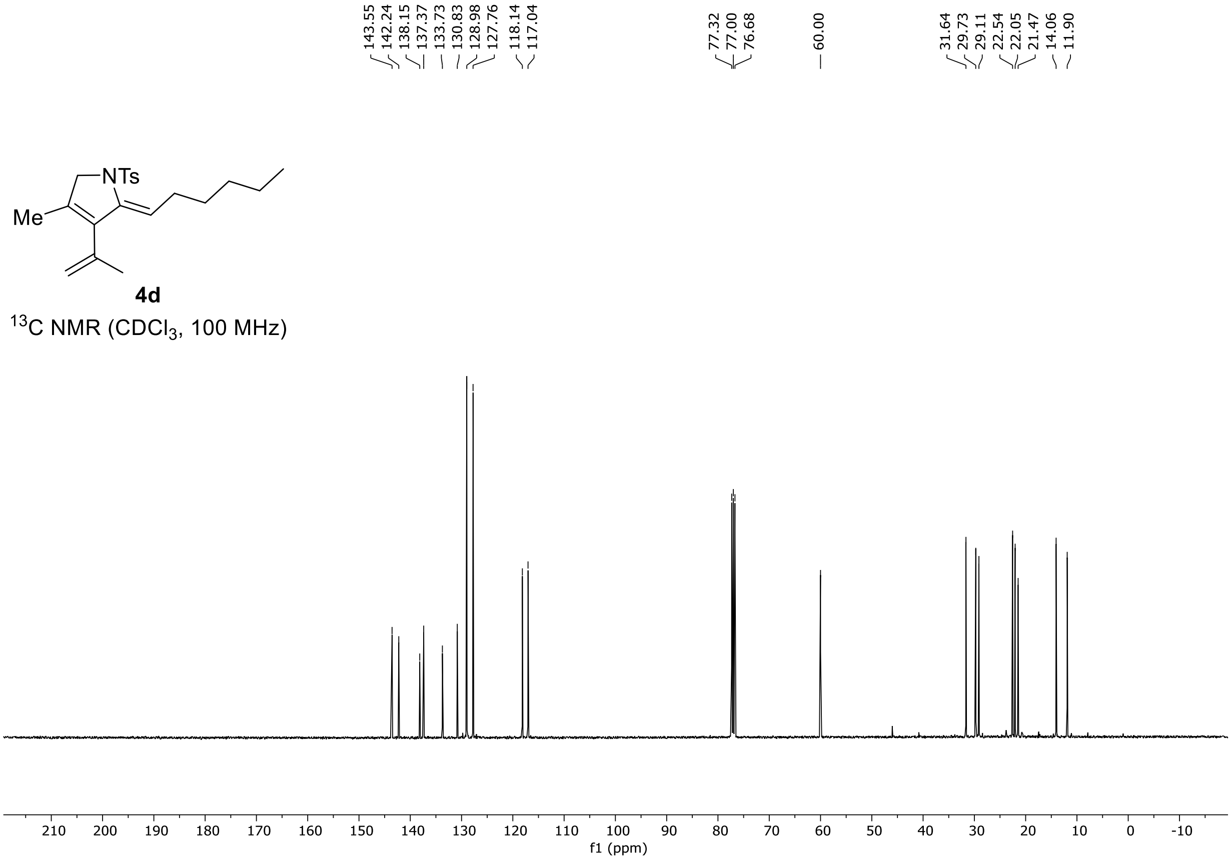

4d

^{13}C NMR (CDCl$_3$, 100 MHz)

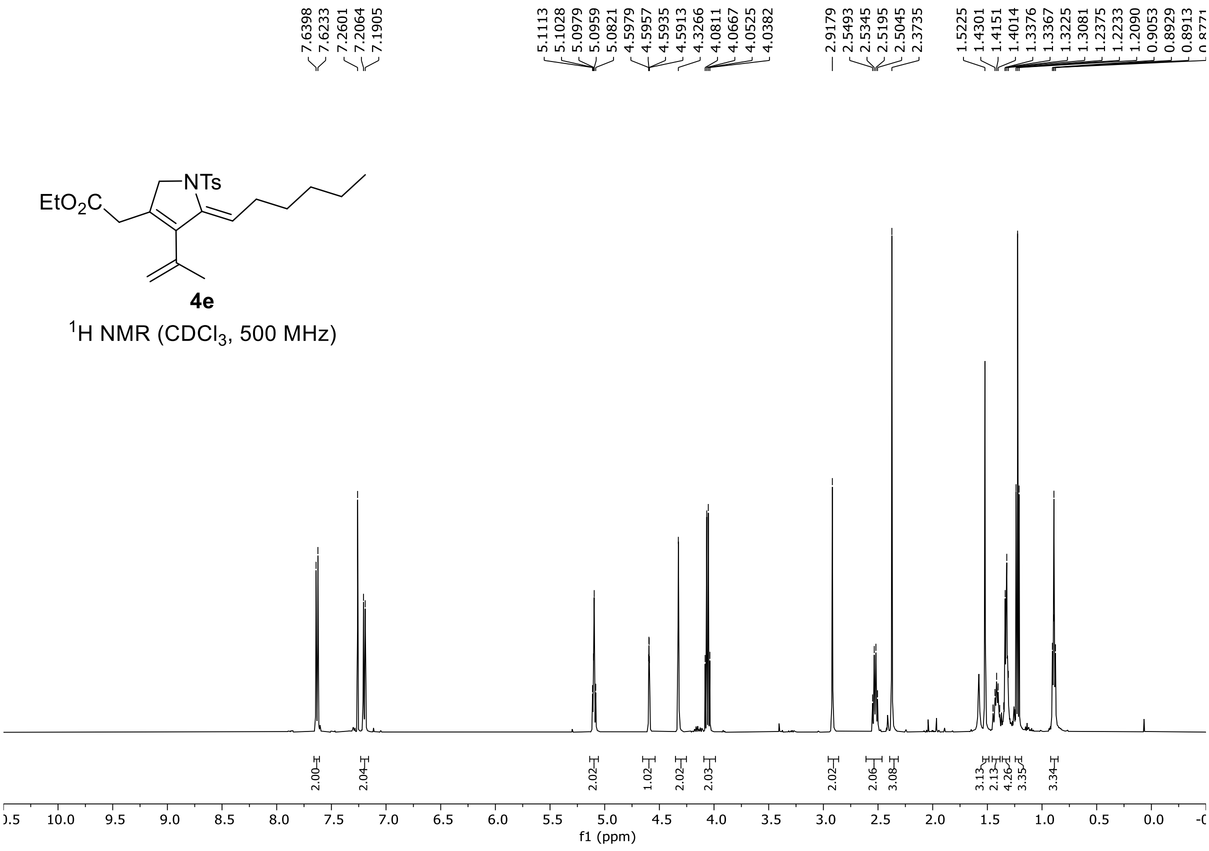

4e

^{1}H NMR ($CDCl_3$, 500 MHz)

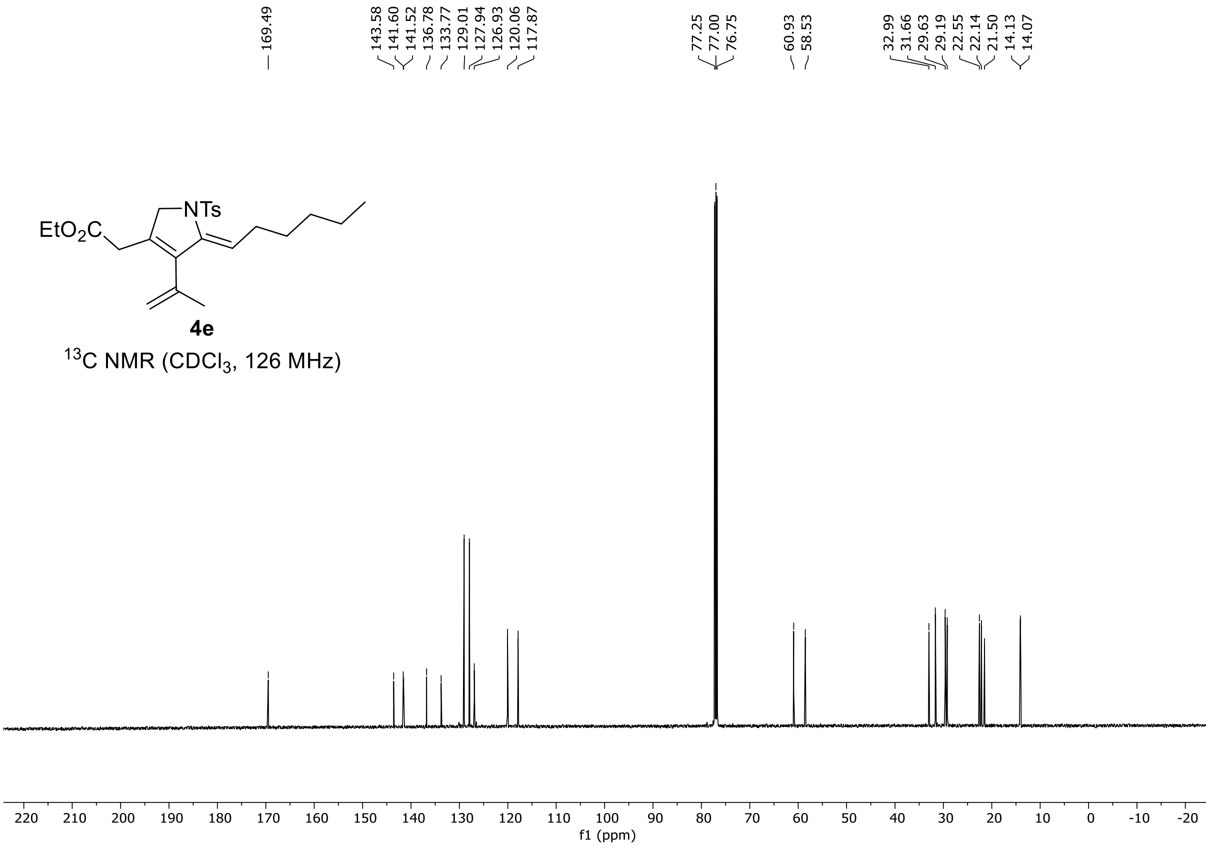

4e

^{13}C NMR ($CDCl_3$, 126 MHz)

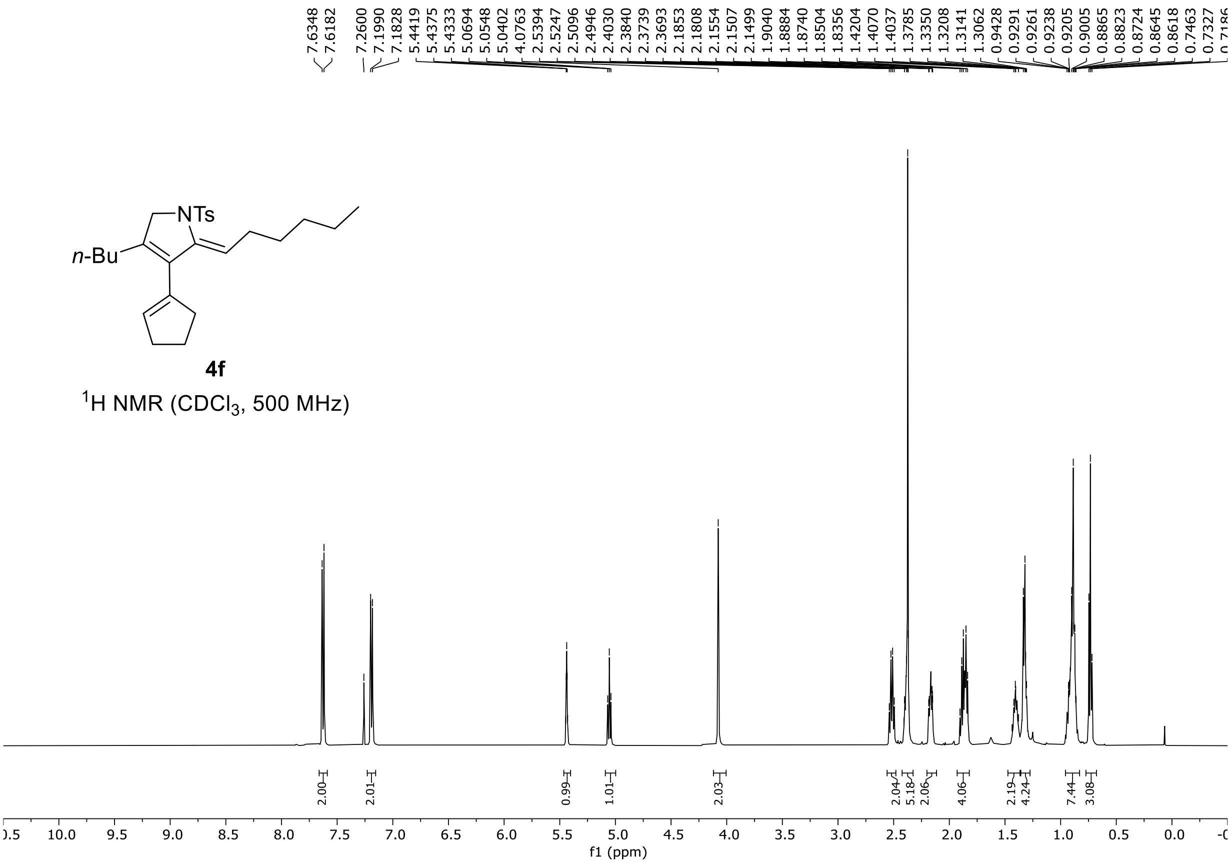
7.6348
7.6182
7.2600
7.1990
7.1828
5.4419
5.4375
5.4333
5.0694
5.0548
5.0402
4.0763
2.5394
2.5247
2.5096
2.4946
2.4030
2.3840
2.3739
2.3693
2.1853
2.1808
2.1554
2.1507
2.1499
1.9040
1.8884
1.8740
1.8504
1.8356
1.4204
1.4070
1.4037
1.3785
1.3350
1.3208
1.3141
1.3062
0.9428
0.9291
0.9261
0.9238
0.9205
0.9005
0.8865
0.8823
0.8724
0.8645
0.8618
0.7463
0.7327
NTs
n-Bu
4f
1H NMR (CDCl3, 500 MHz)
2.00
2.01
0.99
1.01
2.03
2.04
5.18
2.06
4.06
2.19
4.24
7.44
3.08
f1 (ppm)

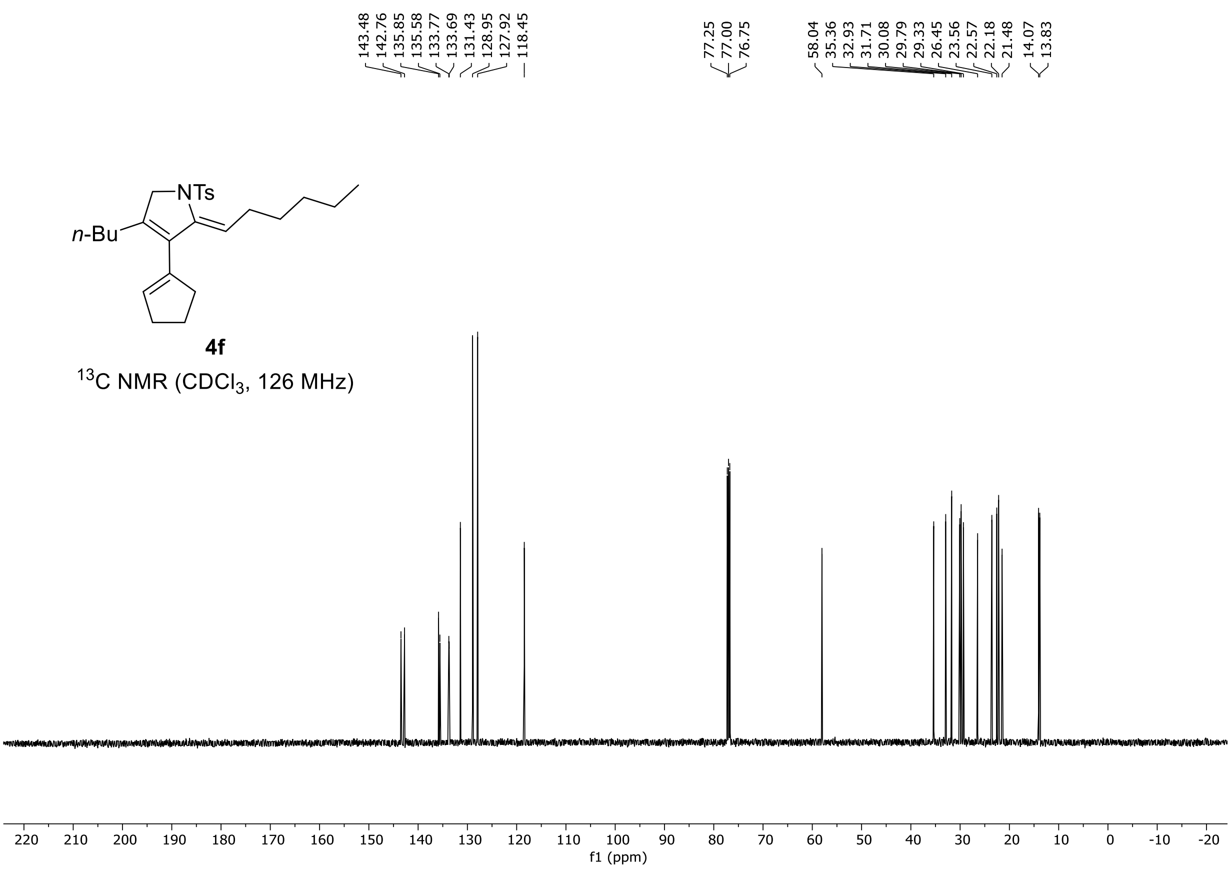
143.48
142.76
135.85
135.58
133.77
133.69
131.43
128.95
127.92
118.45
77.25
77.00
76.75
58.04
35.36
32.93
31.71
30.08
29.79
29.33
26.45
23.56
22.57
22.18
21.48
14.07
13.83
NTs
n-Bu
4f
13C NMR (CDCl3, 126 MHz)
f1 (ppm)

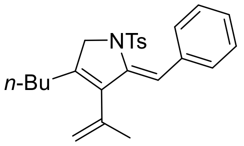

4g

^{1}H NMR ($CDCl_3$, 500 MHz)

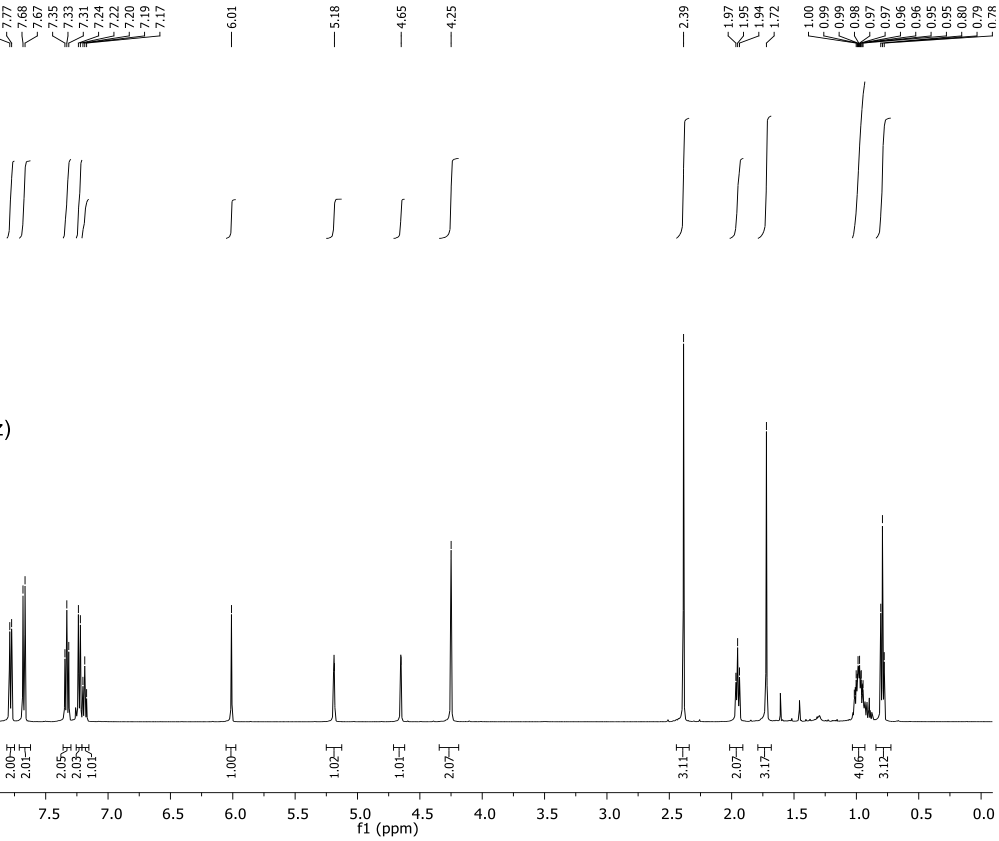

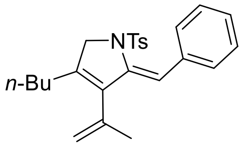

4g

^{13}C NMR ($CDCl_3$, 126 MHz)

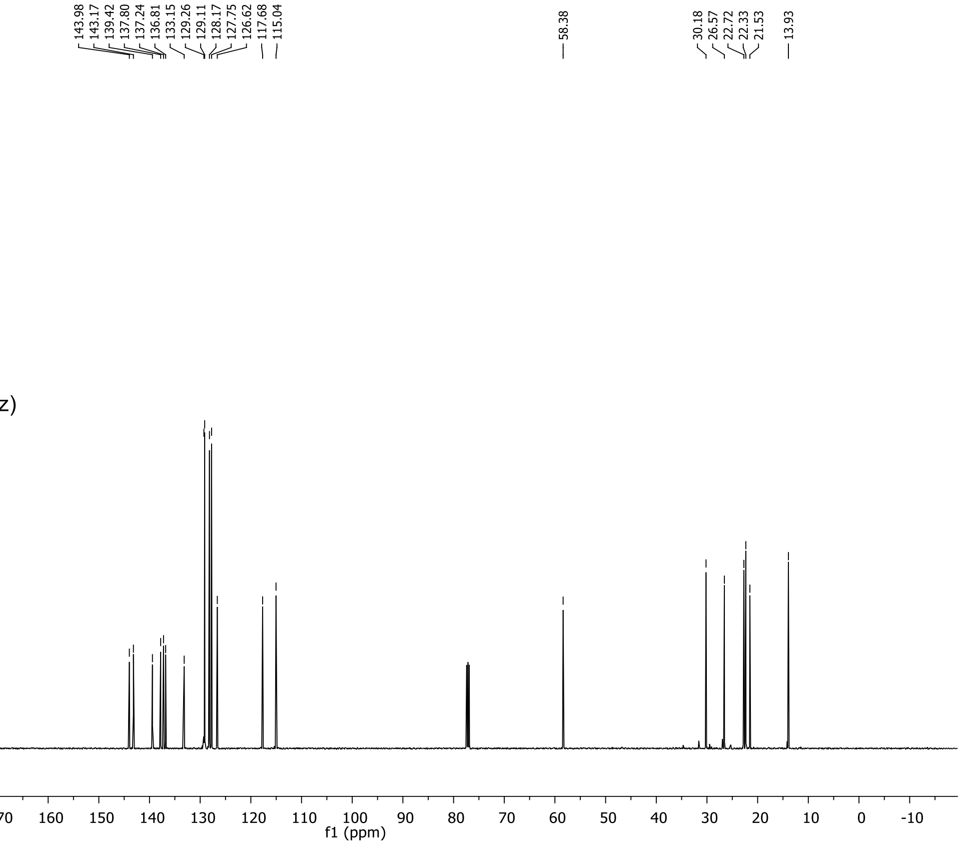

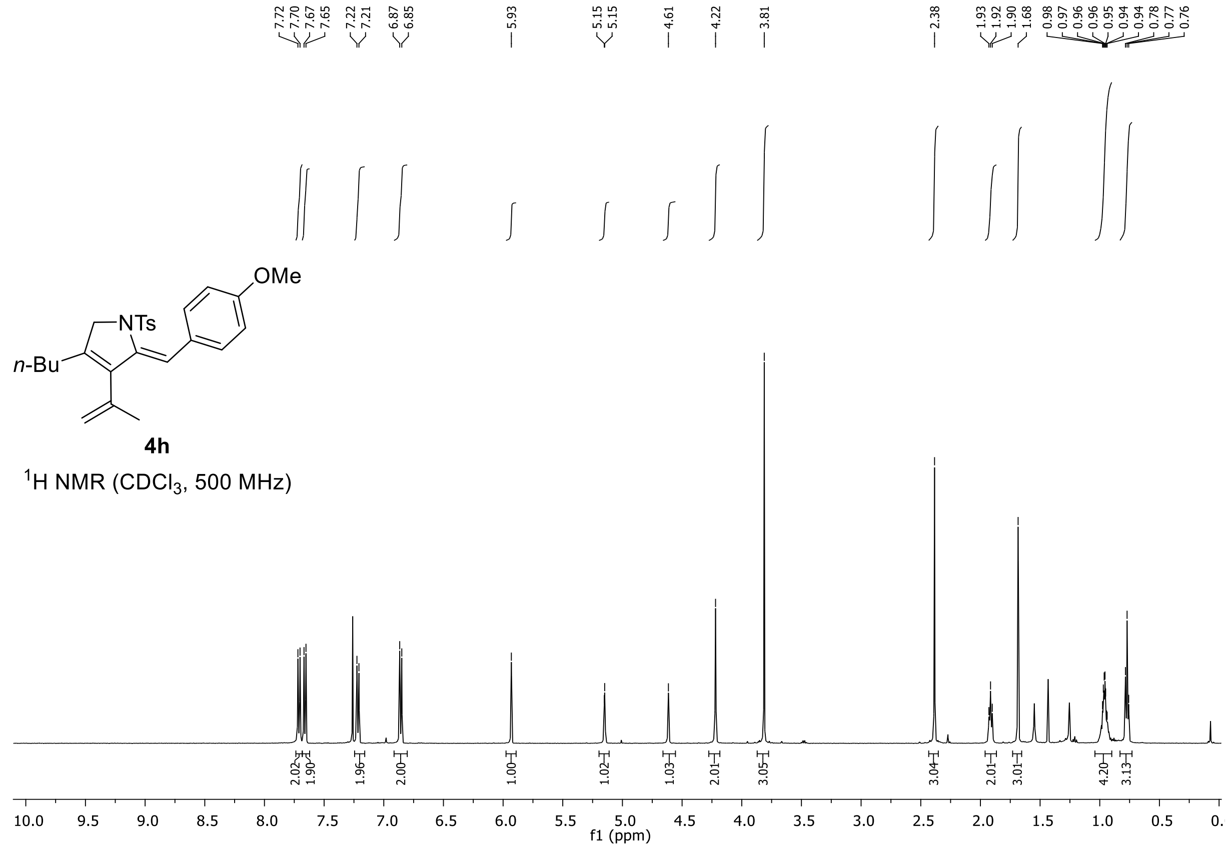
OMe
NTs
n-Bu
4h
1H NMR (CDCl3, 500 MHz)
f1 (ppm)

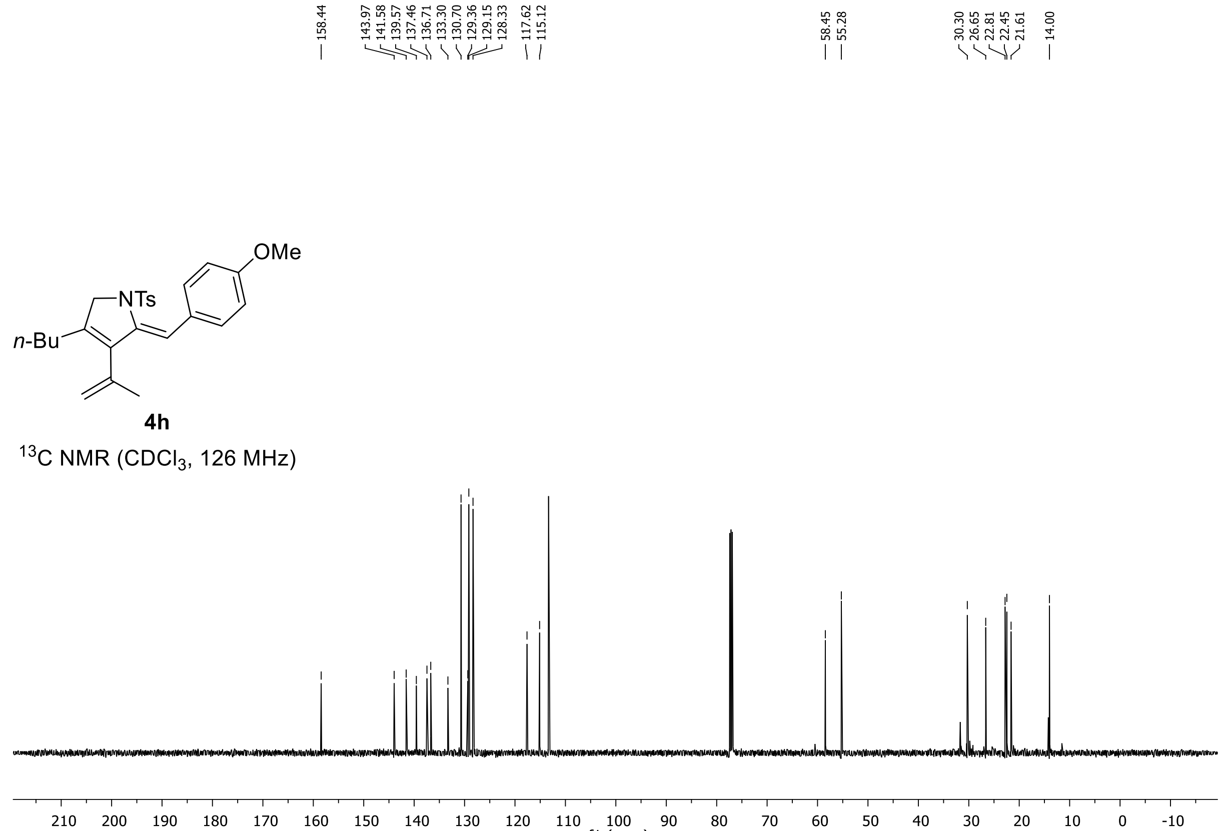
OMe
NTs
n-Bu
4h
13C NMR (CDCl3, 126 MHz)
f1 (ppm)

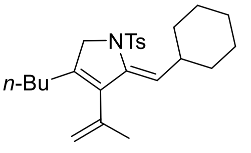

4i

^{1}H NMR ($CDCl_3$, 500 MHz)

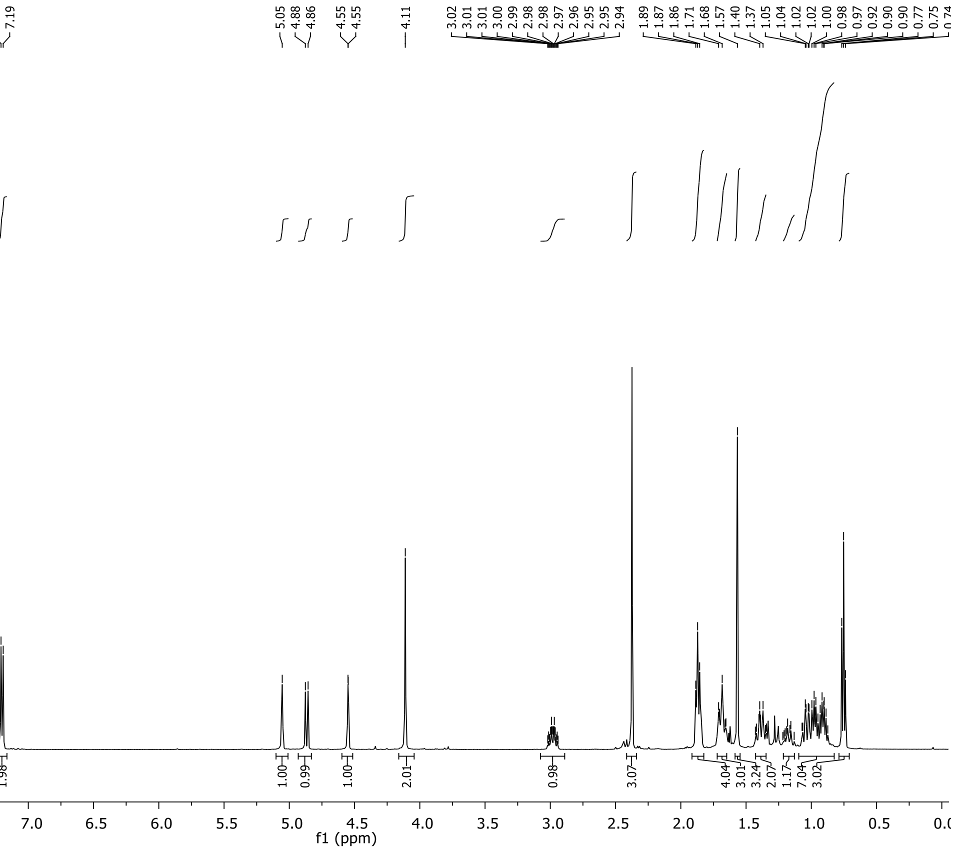

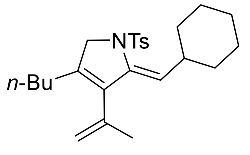

4i

^{13}C NMR ($CDCl_3$, 126 MHz)

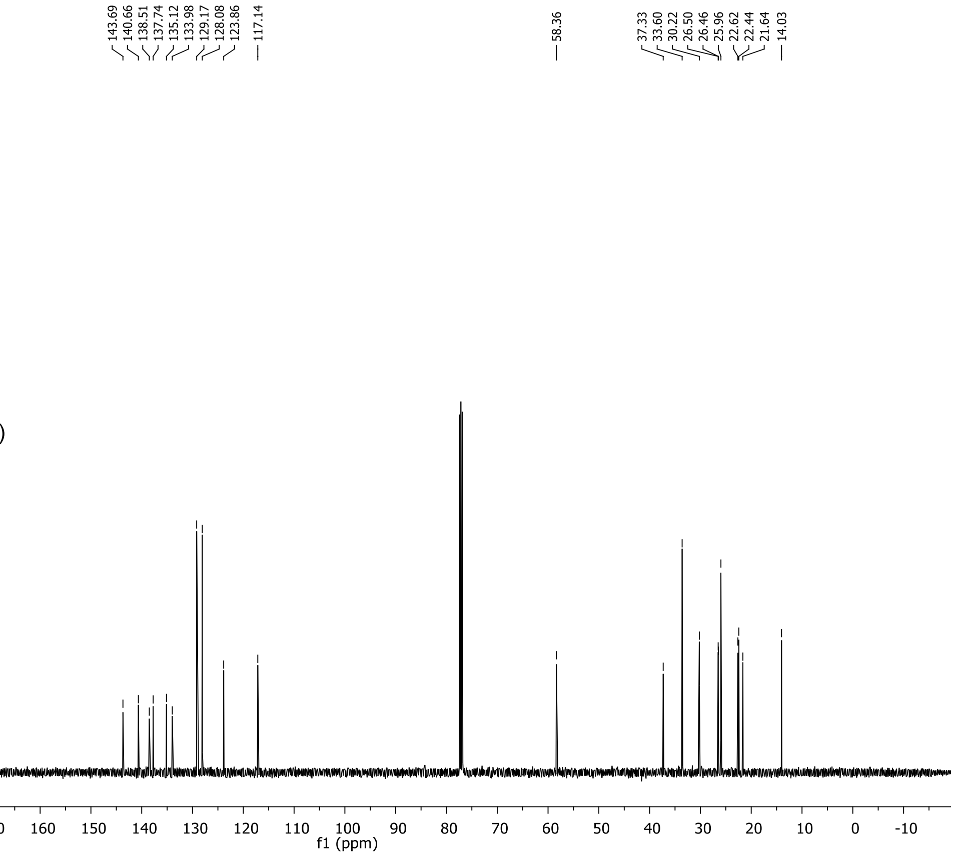

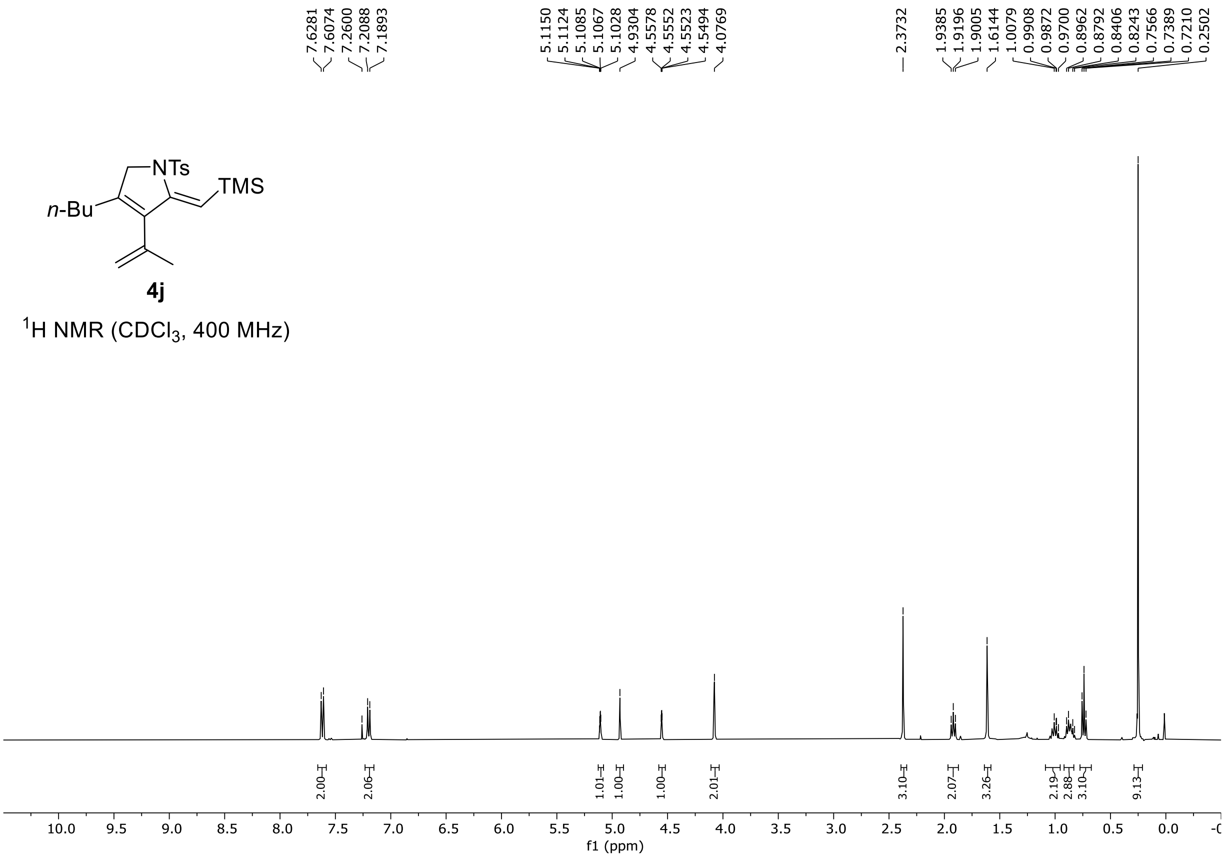
7.6281
7.6074
7.2600
7.2088
7.1893
5.1150
5.1124
5.1085
5.1067
5.1028
4.9304
4.5578
4.5552
4.5523
4.5494
4.0769
2.3732
1.9385
1.9196
1.9005
1.6144
1.0079
0.9908
0.9872
0.9700
0.8962
0.8792
0.8406
0.8243
0.7566
0.7389
0.7210
0.2502
NTs
TMS
n-Bu
4j
^{1}H NMR ($CDCl_3$, 400 MHz)
2.00
2.06
1.01
1.00
1.00
2.01
3.10
2.07
3.26
2.19
2.88
3.10
9.13
f1 (ppm)

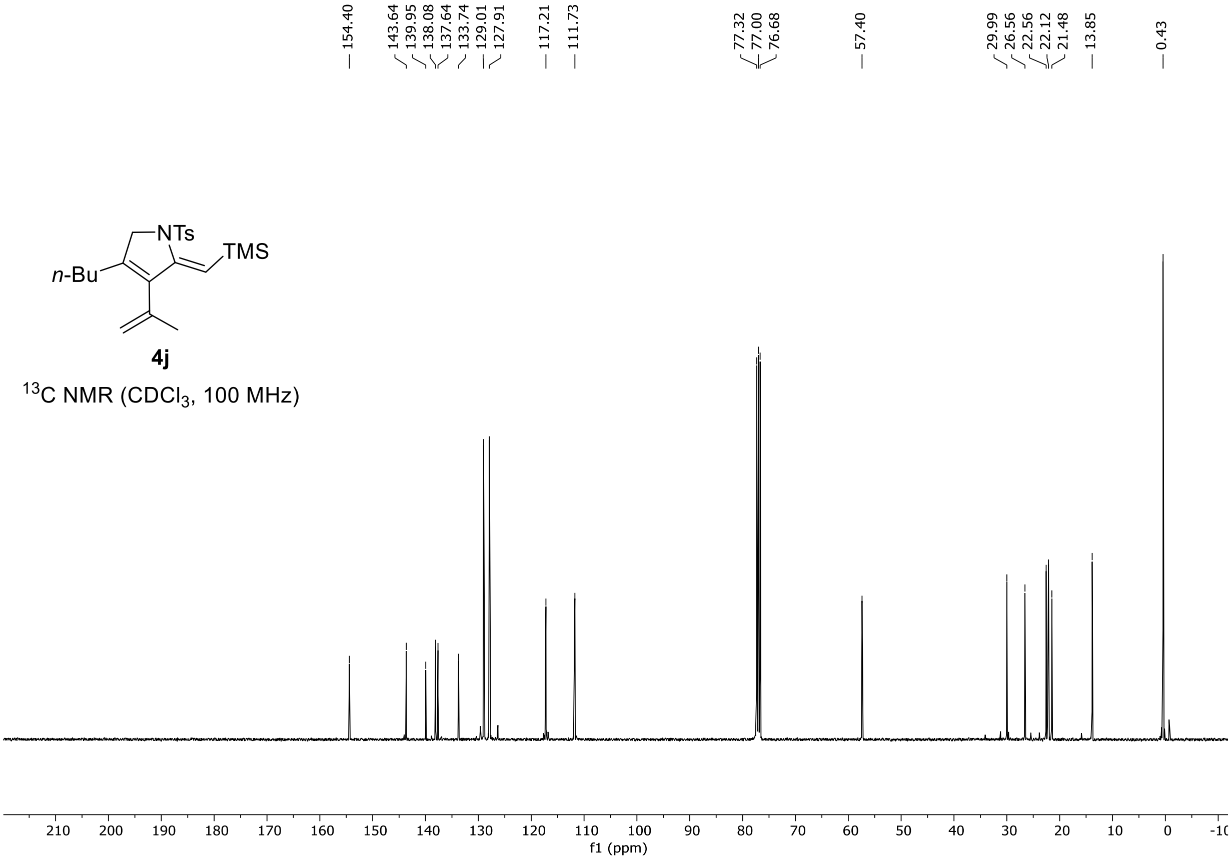
154.40
143.64
139.95
138.08
137.64
133.74
129.01
127.91
117.21
111.73
77.32
77.00
76.68
57.40
29.99
26.56
22.56
22.12
21.48
13.85
0.43
NTs
TMS
n-Bu
4j
^{13}C NMR ($CDCl_3$, 100 MHz)
f1 (ppm)

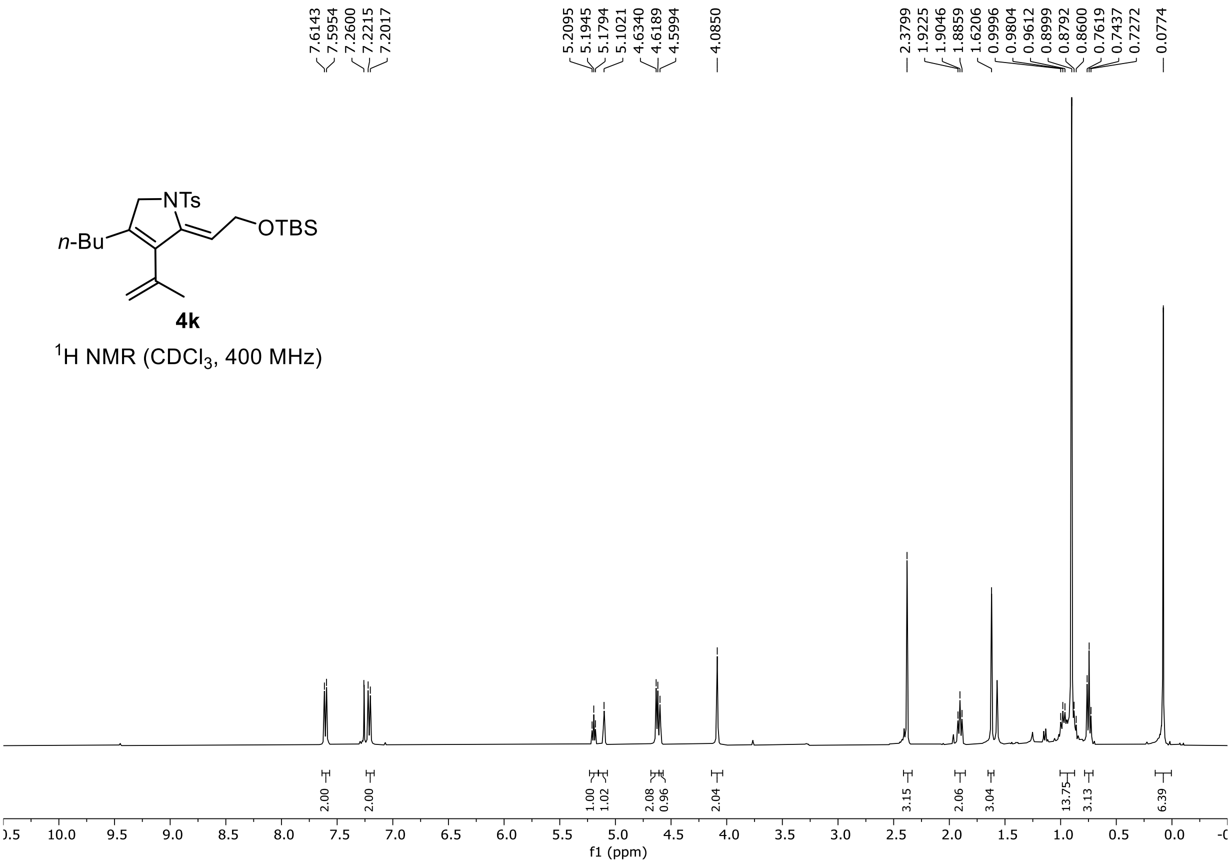
7.6143
7.5954
7.2600
7.2215
7.2017
5.2095
5.1945
5.1794
5.1021
4.6340
4.6189
4.5994
4.0850
2.3799
1.9225
1.9046
1.8859
1.6206
0.9996
0.9804
0.9612
0.8999
0.8792
0.8600
0.7619
0.7437
0.7272
0.0774
NTs
n-Bu
OTBS
4k
1H NMR (CDCl3, 400 MHz)
2.00
2.00
1.00
1.02
2.08
0.96
2.04
3.15
2.06
3.04
13.75
3.13
6.39
f1 (ppm)

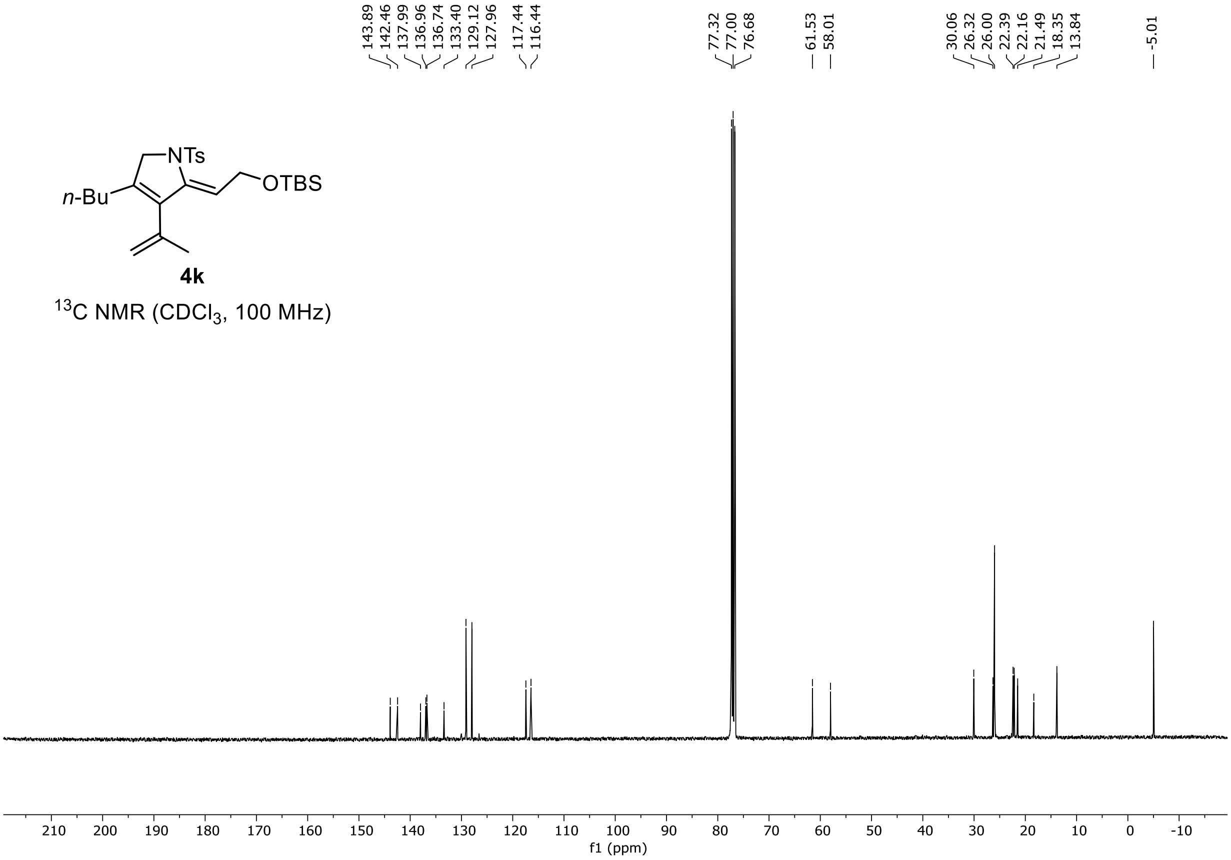
143.89
142.46
137.99
136.96
136.74
133.40
129.12
127.96
117.44
116.44
77.32
77.00
76.68
61.53
58.01
30.06
26.32
26.00
22.39
22.16
21.49
18.35
13.84
-5.01
NTs
n-Bu
OTBS
4k
13C NMR (CDCl3, 100 MHz)
f1 (ppm)

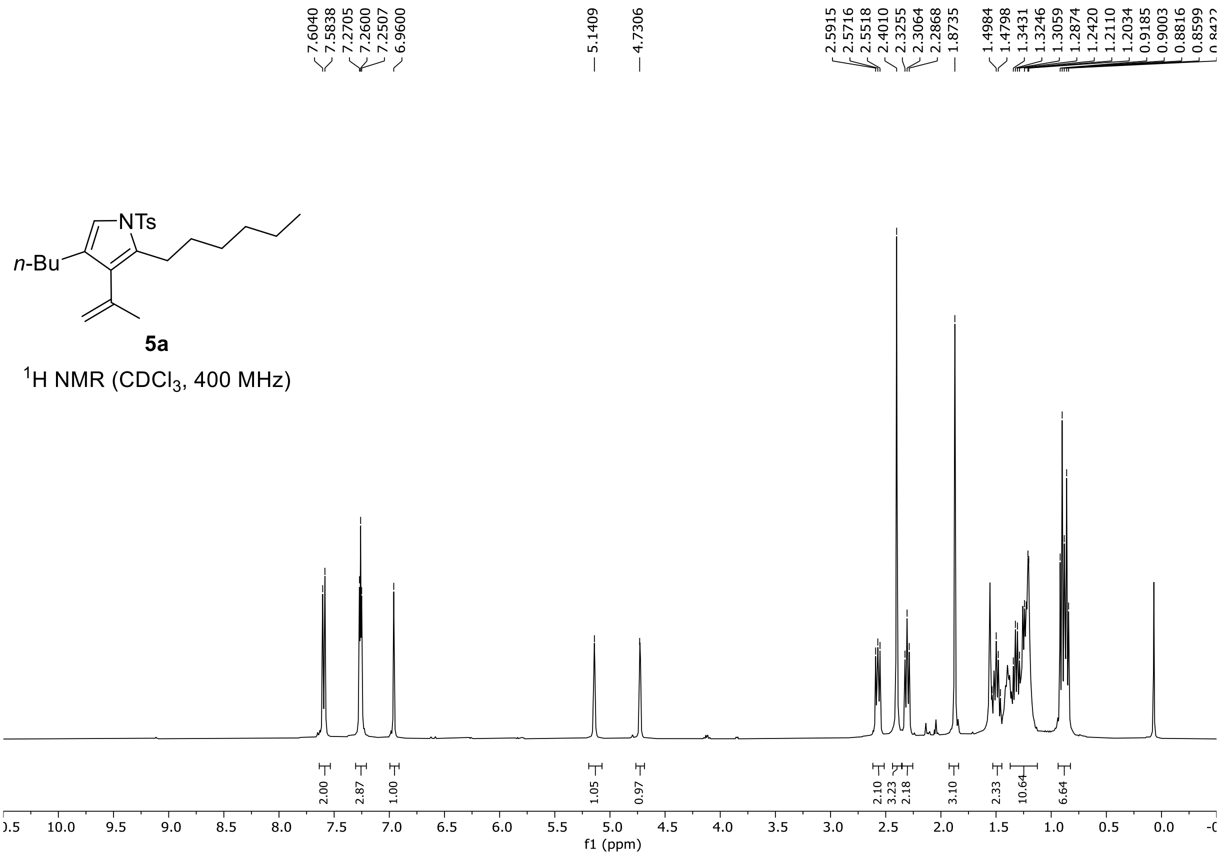

5a

^{1}H NMR ($CDCl_3$, 400 MHz)

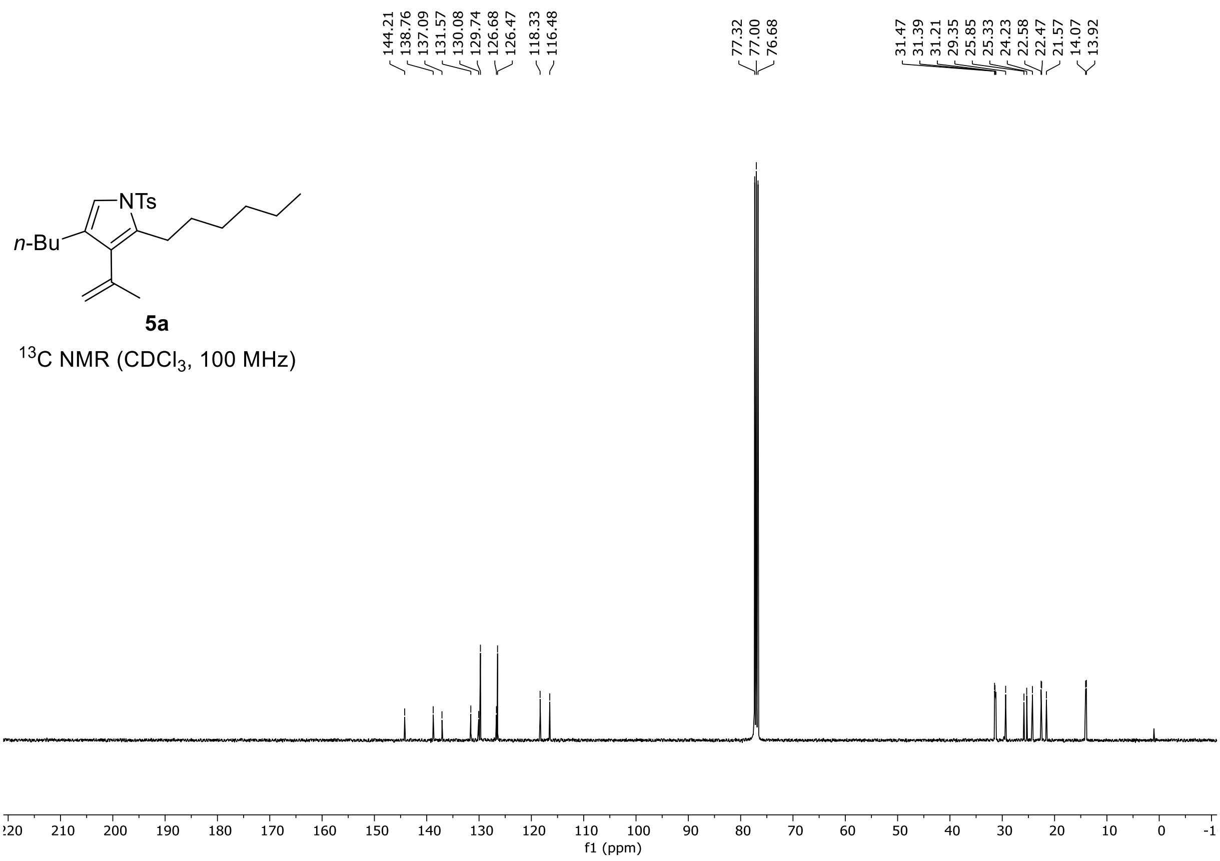

5a

^{13}C NMR ($CDCl_3$, 100 MHz)

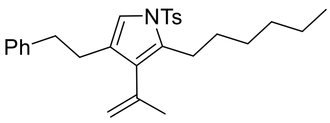

5b

^{1}H NMR ($CDCl_3$, 400 MHz)

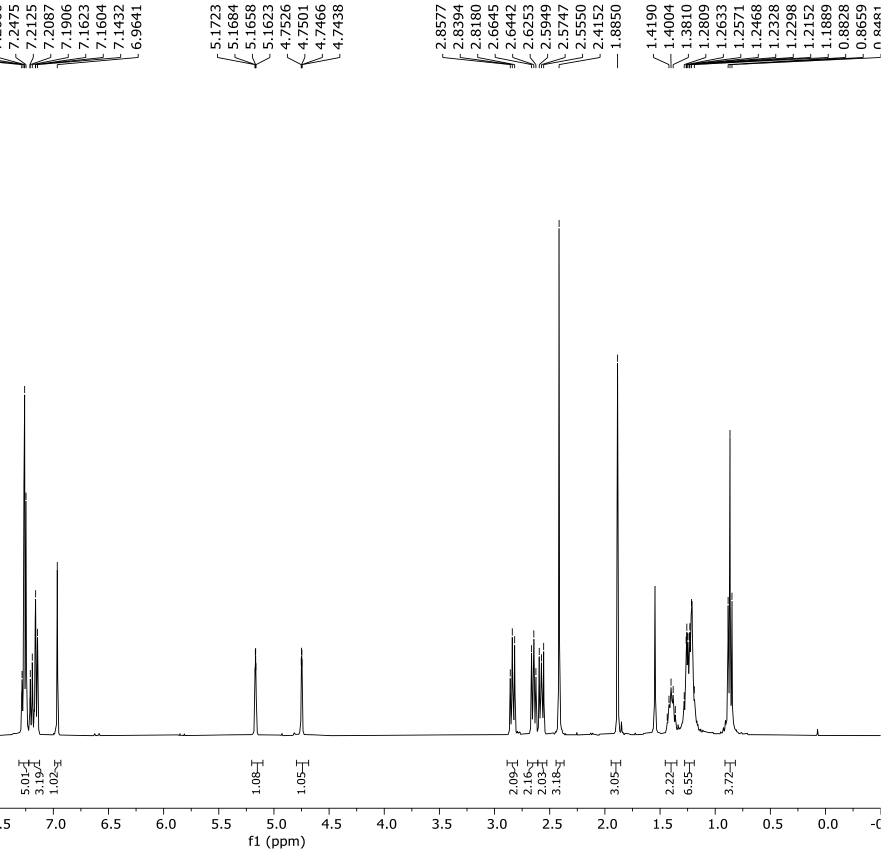

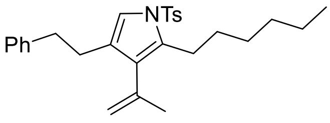

5b

^{13}C NMR ($CDCl_3$, 100 MHz)

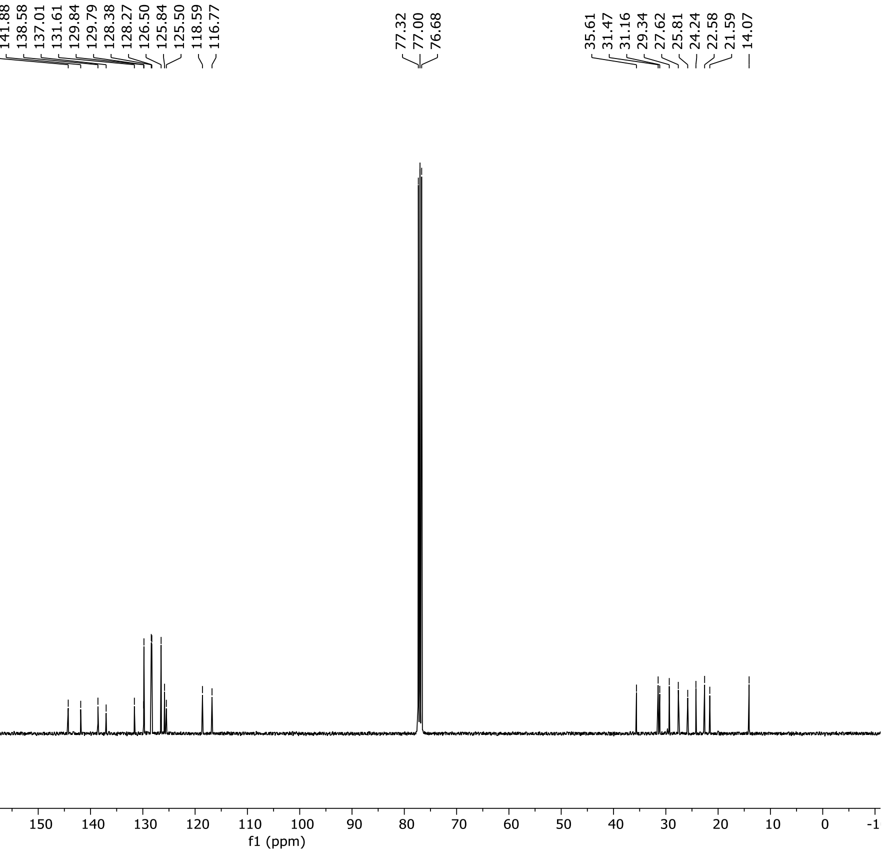

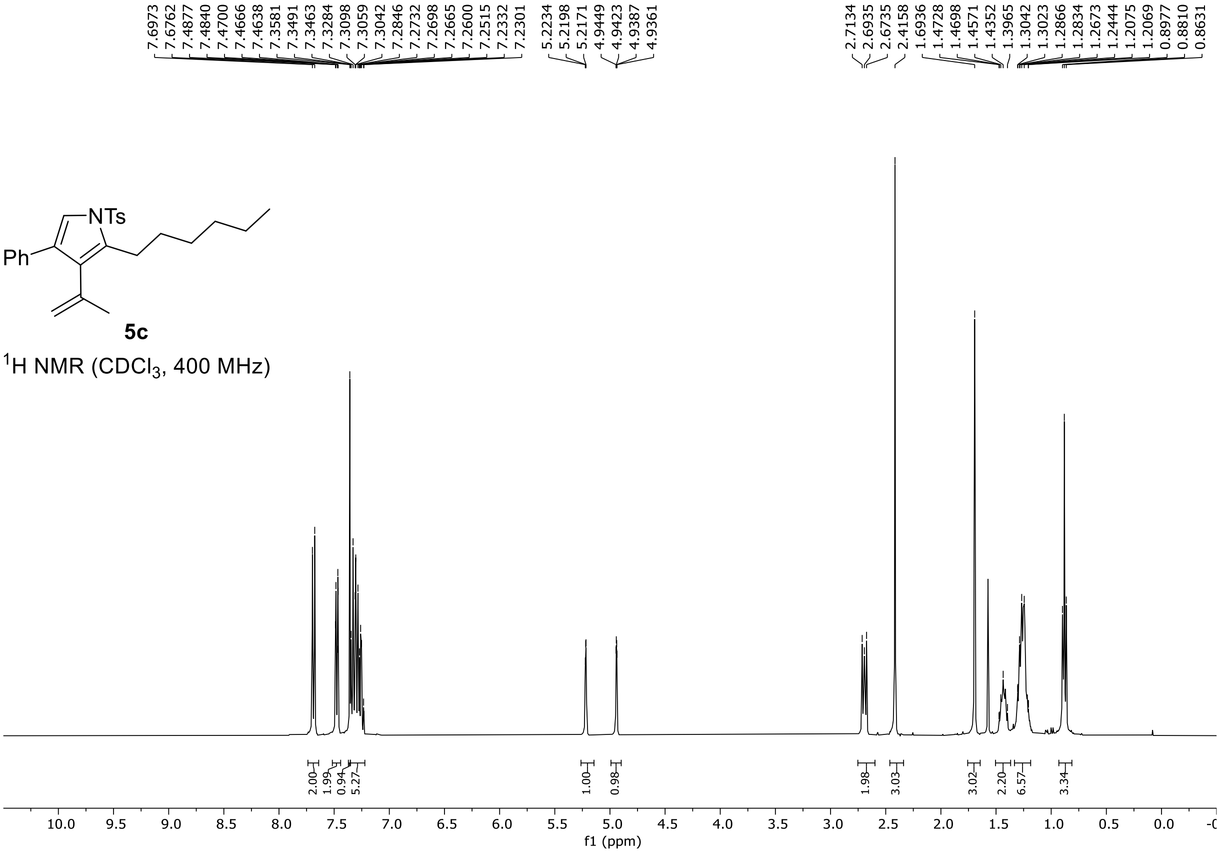
7.6973 7.6762 7.4877 7.4840 7.4700 7.4666 7.4638 7.3581 7.3491 7.3463 7.3284 7.3098 7.3059 7.3042 7.2846 7.2732 7.2698 7.2665 7.2600 7.2515 7.2332 7.2301
5.2234 5.2198 5.2171 4.9449 4.9423 4.9387 4.9361
2.7134 2.6935 2.6735 2.4158 1.6936 1.4728 1.4698 1.4571 1.4352 1.3965 1.3042 1.3023 1.2866 1.2834 1.2673 1.2444 1.2075 1.2069 0.8977 0.8810 0.8631
NTs
Ph
5c
1H NMR (CDCl3, 400 MHz)
2.00 1.99 0.94 5.27 1.00 0.98 1.98 3.03 3.02 2.20 6.57 3.34
f1 (ppm)

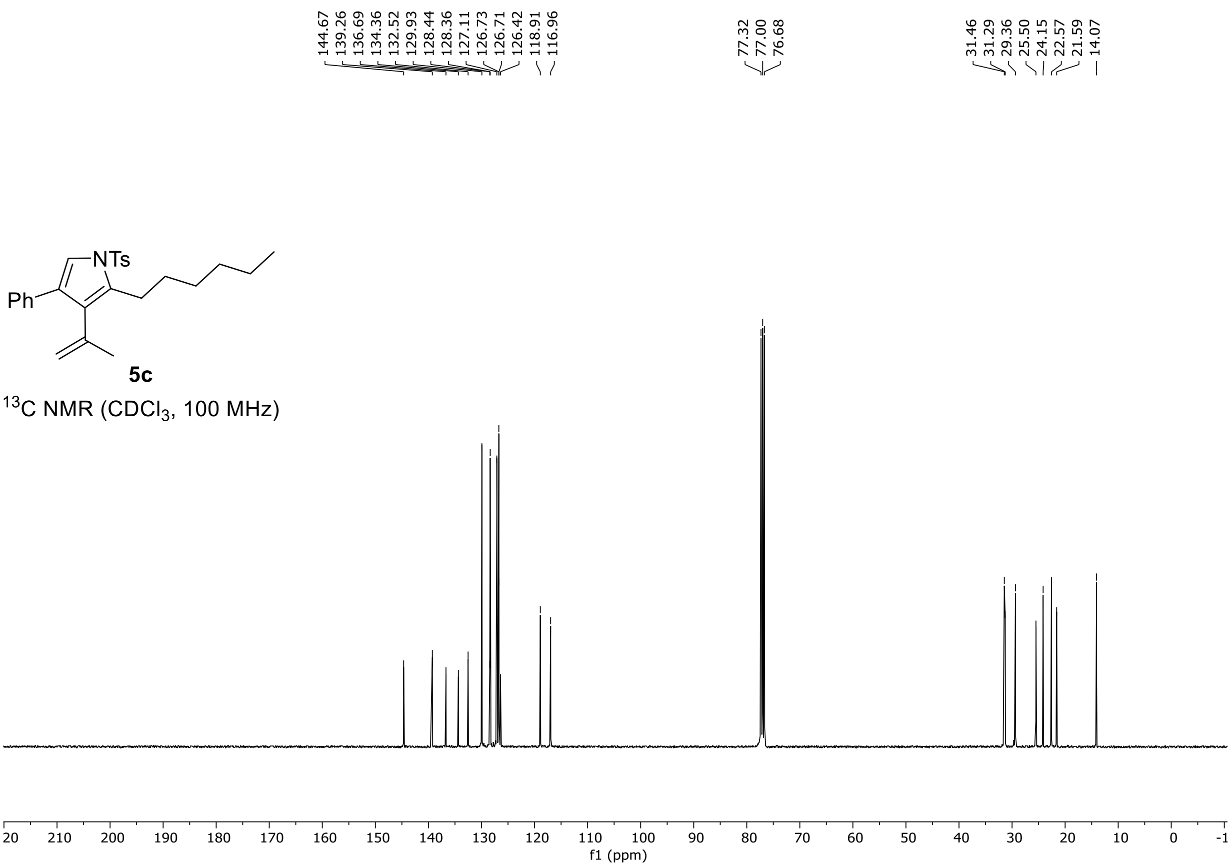
144.67 139.26 136.69 134.36 132.52 129.93 128.44 128.36 127.11 126.73 126.71 126.42 118.91 116.96
77.32 77.00 76.68
31.46 31.29 29.36 25.50 24.15 22.57 21.59 14.07
NTs
Ph
5c
13C NMR (CDCl3, 100 MHz)
f1 (ppm)

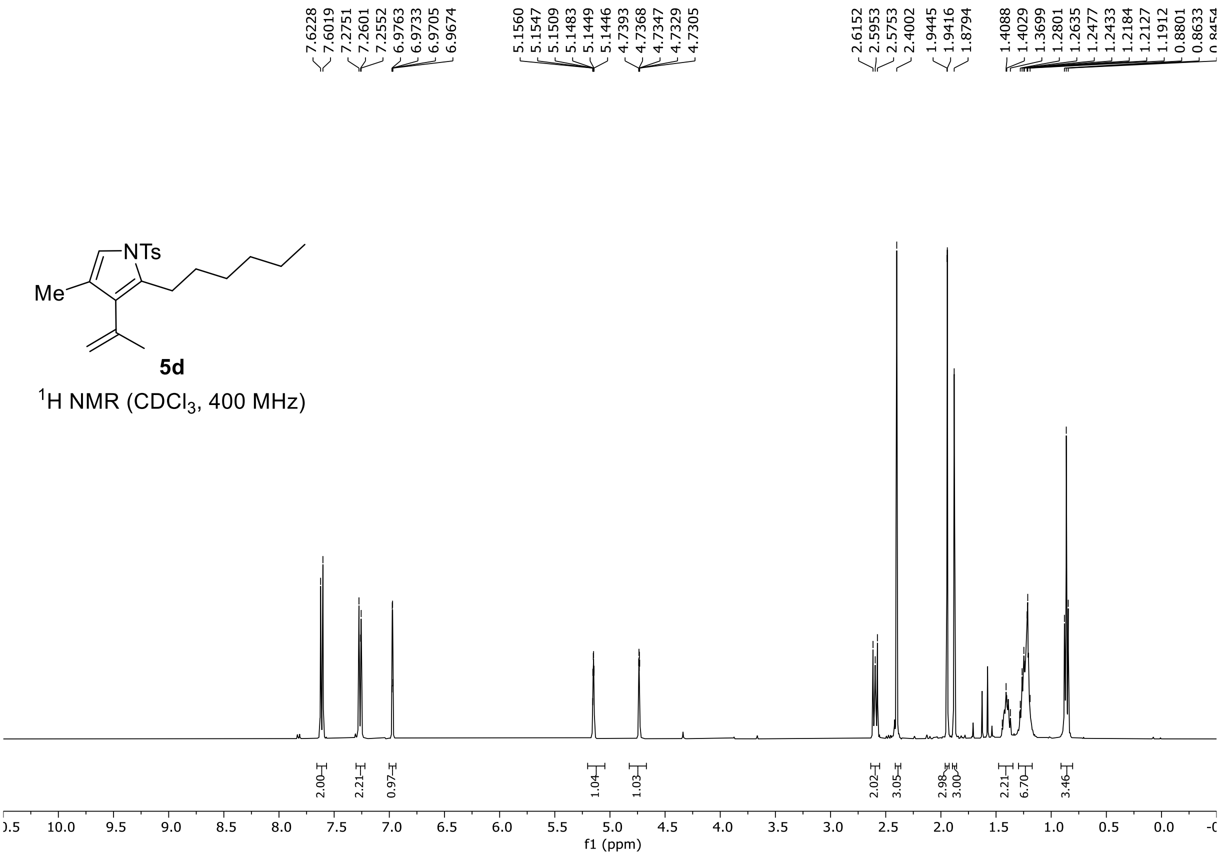

5d

^{1}H NMR ($CDCl_3$, 400 MHz)

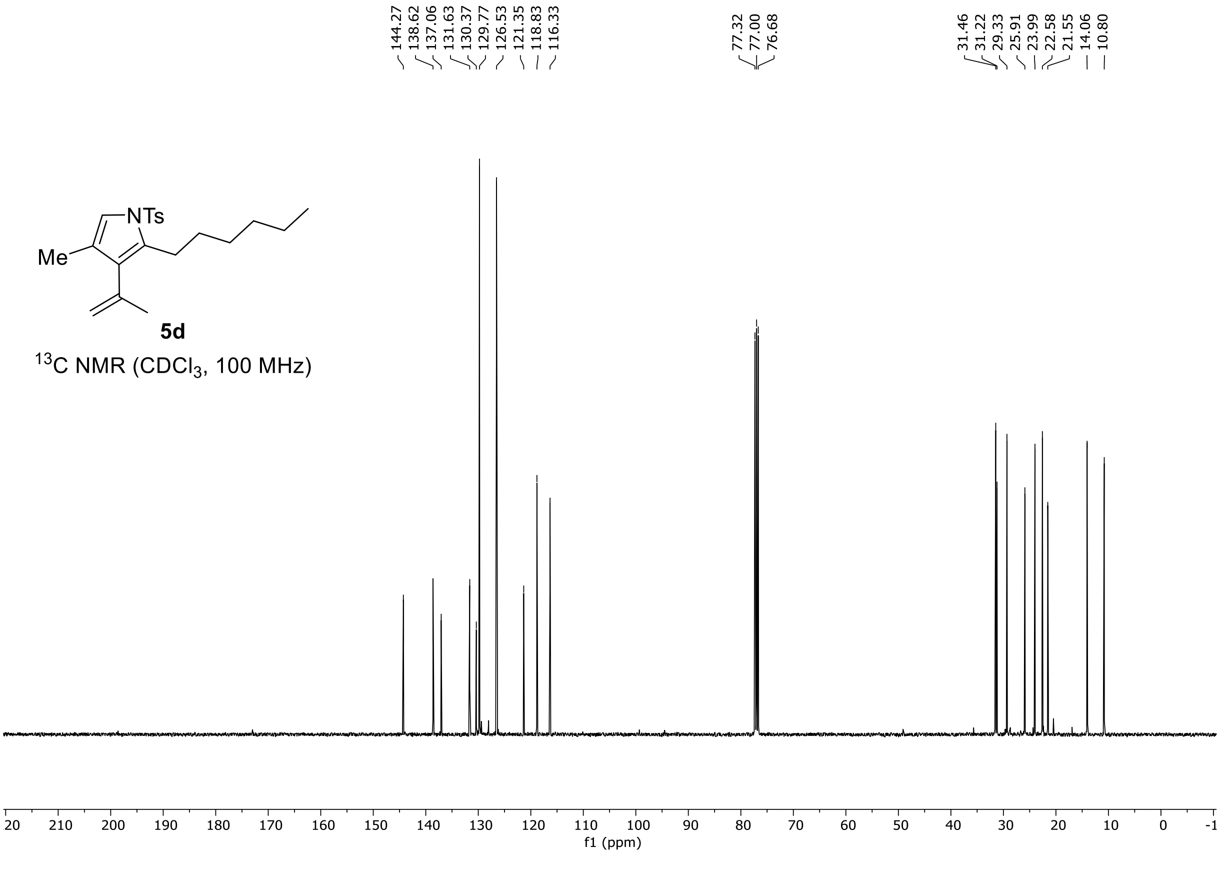

5d

^{13}C NMR ($CDCl_3$, 100 MHz)

5e

^{1}H NMR ($CDCl_3$, 400 MHz)

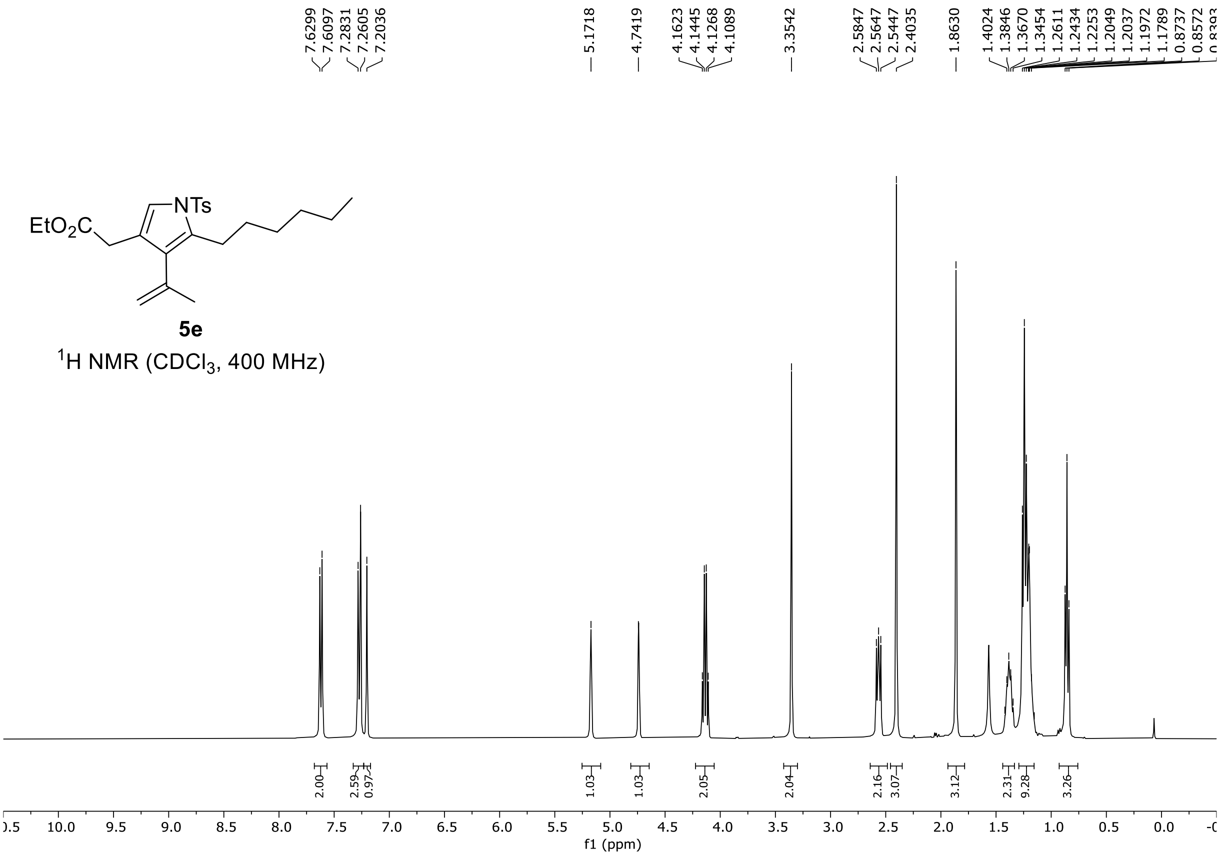

5e

^{13}C NMR ($CDCl_3$, 100 MHz)

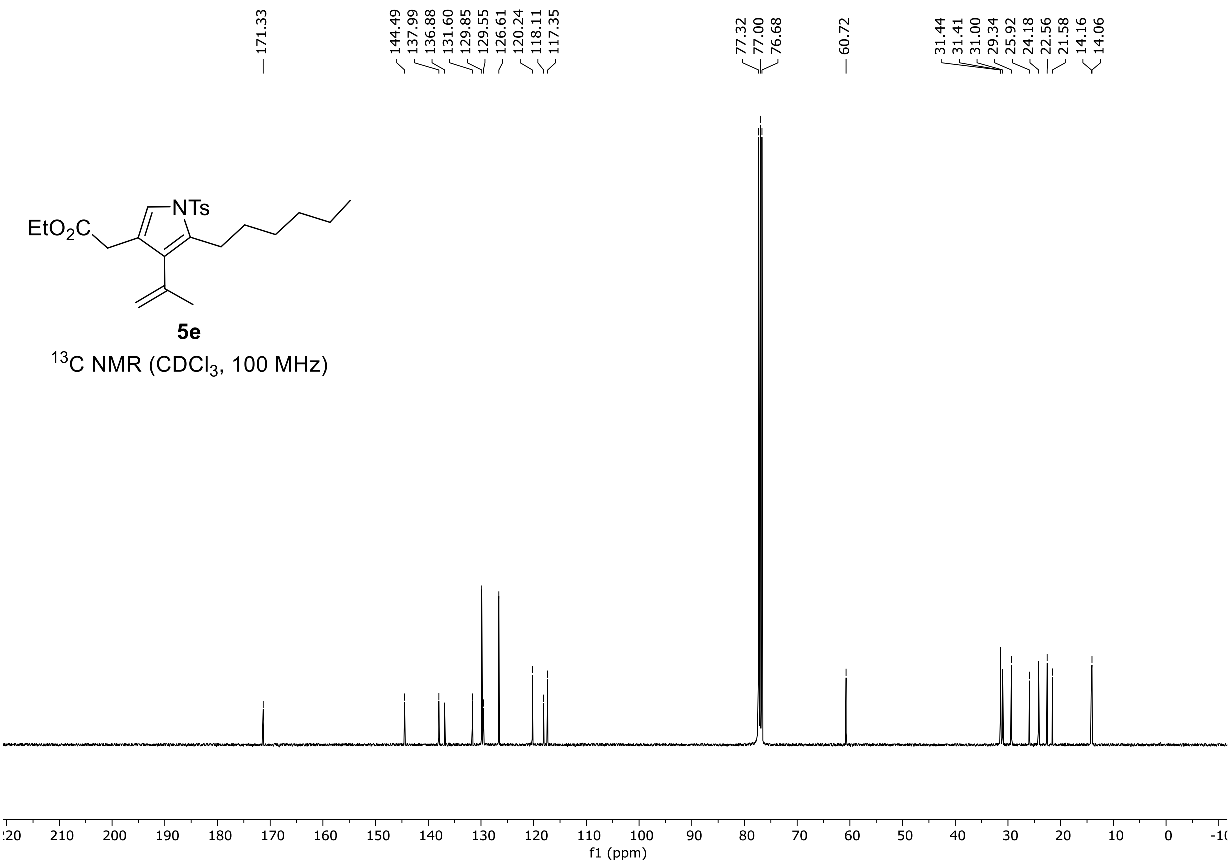

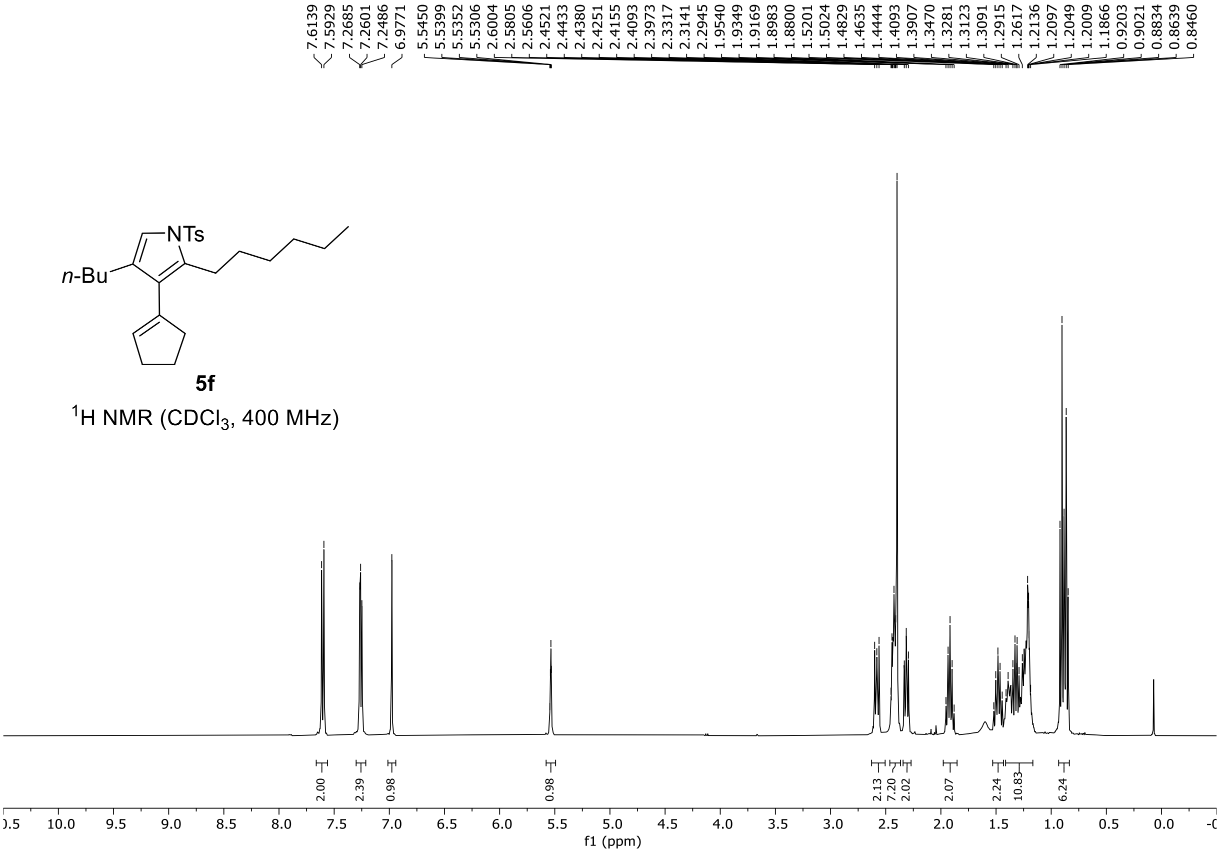

5f

^{1}H NMR ($CDCl_3$, 400 MHz)

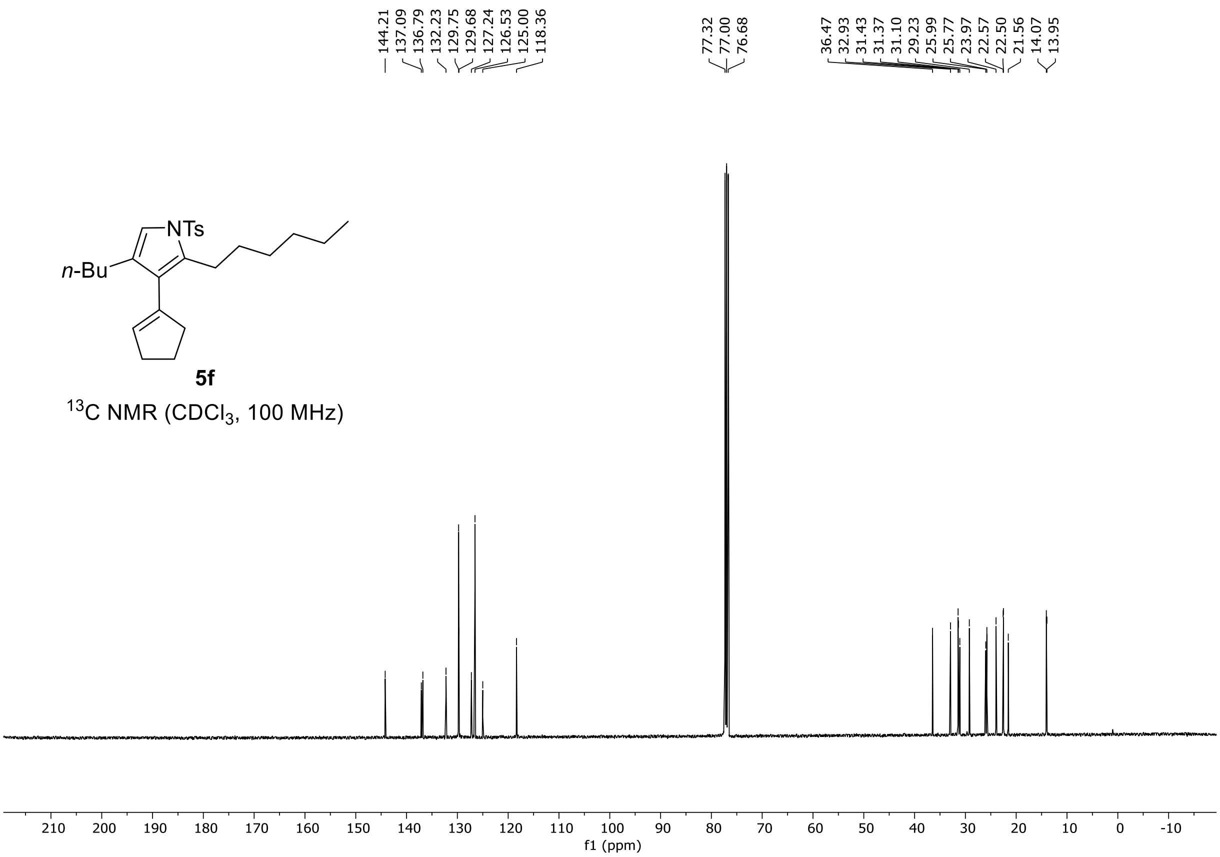

5f

^{13}C NMR ($CDCl_3$, 100 MHz)

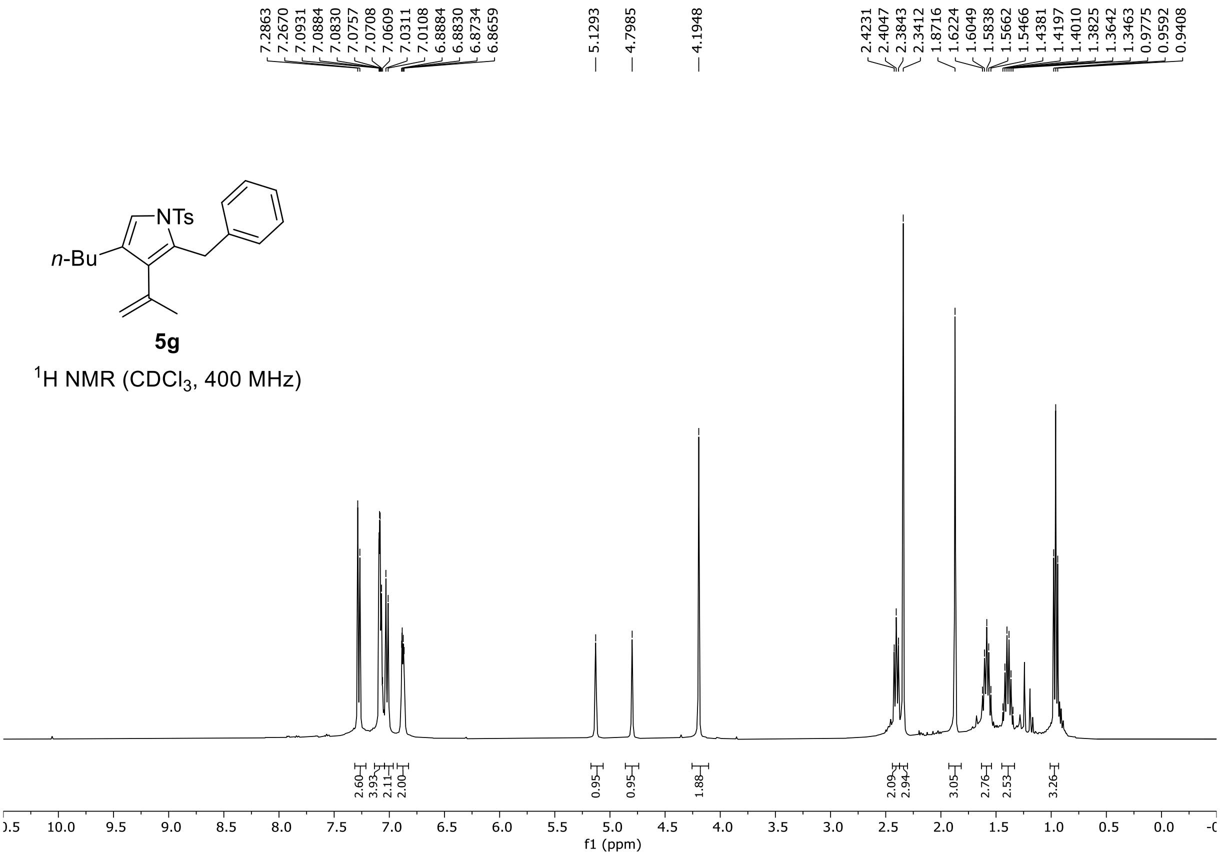
7.2863 7.2670 7.0931 7.0884 7.0830 7.0757 7.0708 7.0609 7.0311 7.0108 6.8884 6.8830 6.8734 6.8659
5.1293
4.7985
4.1948
2.4231 2.4047 2.3843 2.3412 1.8716 1.6224 1.6049 1.5838 1.5662 1.5466 1.4381 1.4197 1.4010 1.3825 1.3642 1.3463 0.9775 0.9592 0.9408
NTs
n-Bu
5g
1H NMR (CDCl3, 400 MHz)
2.60 3.93 2.11 2.00 0.95 0.95 1.88 2.09 2.94 3.05 2.76 2.53 3.26
10.0 9.5 9.0 8.5 8.0 7.5 7.0 6.5 6.0 5.5 5.0 4.5 4.0 3.5 3.0 2.5 2.0 1.5 1.0 0.5 0.0
f1 (ppm)

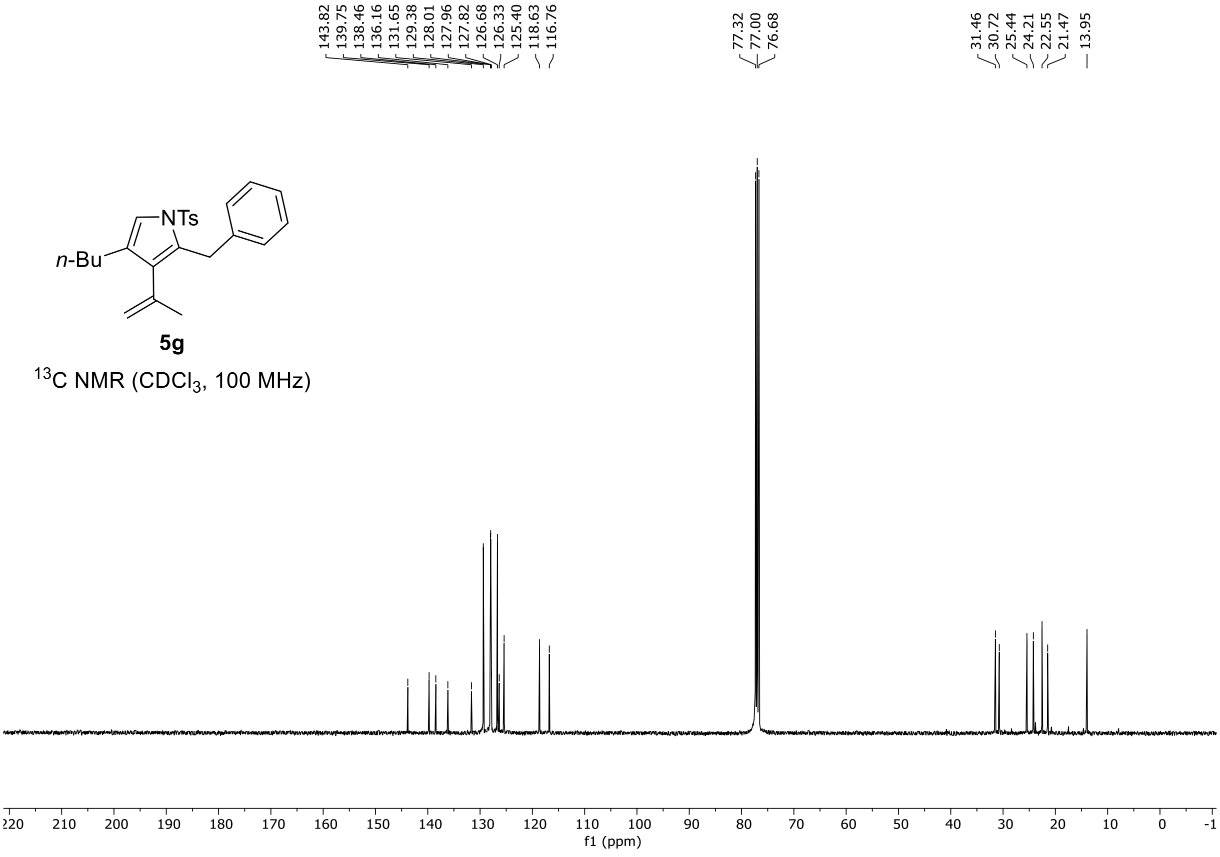
143.82 139.75 138.46 136.16 131.65 129.38 128.01 127.96 127.82 126.68 126.33 125.40 118.63 116.76
77.32 77.00 76.68
31.46 30.72 25.44 24.21 22.55 21.47 13.95
NTs
n-Bu
5g
13C NMR (CDCl3, 100 MHz)
210 200 190 180 170 160 150 140 130 120 110 100 90 80 70 60 50 40 30 20 10 0
f1 (ppm)

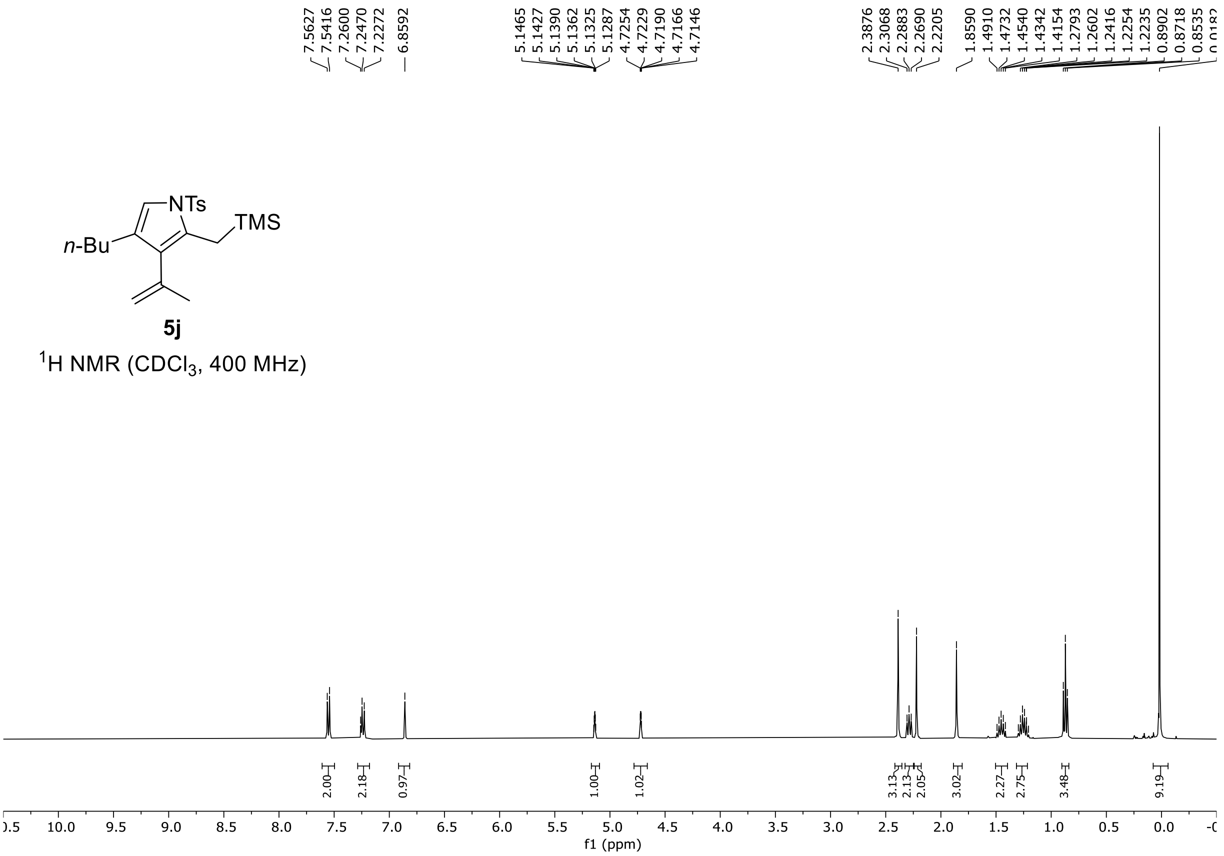
7.5627
7.5416
7.2600
7.2470
7.2272
6.8592
5.1465
5.1427
5.1390
5.1362
5.1325
5.1287
4.7254
4.7229
4.7190
4.7166
4.7146
2.3876
2.3068
2.2883
2.2690
2.2205
1.8590
1.4910
1.4732
1.4540
1.4342
1.4154
1.2793
1.2602
1.2416
1.2254
1.2235
0.8902
0.8718
0.8535
NTs
TMS
n-Bu
5j
1H NMR (CDCl3, 400 MHz)
2.00
2.18
0.97
1.00
1.02
3.13
2.13
2.05
3.02
2.27
2.75
3.48
9.19
f1 (ppm)

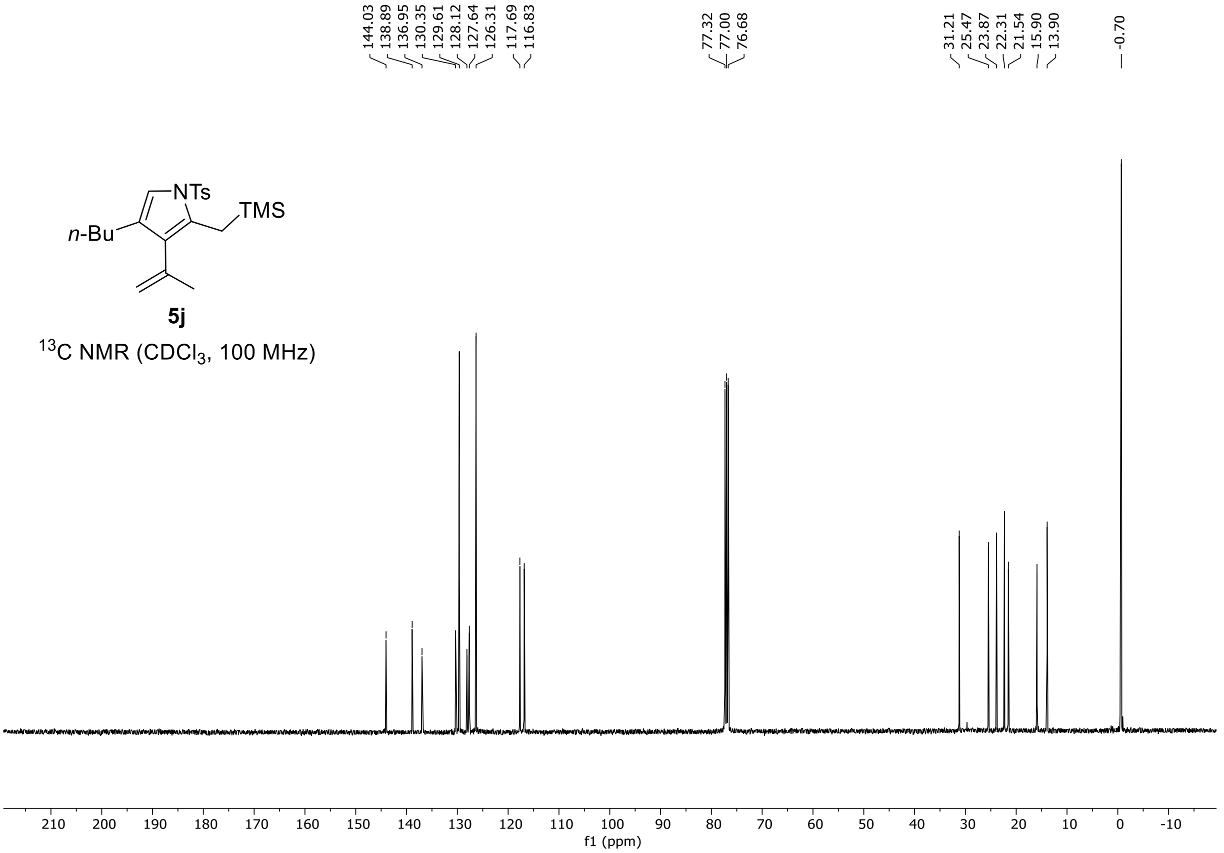
144.03
138.89
136.95
130.35
129.61
128.12
127.64
126.31
117.69
116.83
77.32
77.00
76.68
31.21
25.47
23.87
22.31
21.54
15.90
13.90
-0.70
NTs
TMS
n-Bu
5j
13C NMR (CDCl3, 100 MHz)
f1 (ppm)

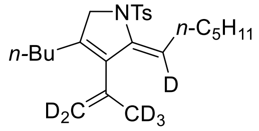

d[6]-**4a**

^{1}H NMR ($CDCl_3$, 400 MHz)

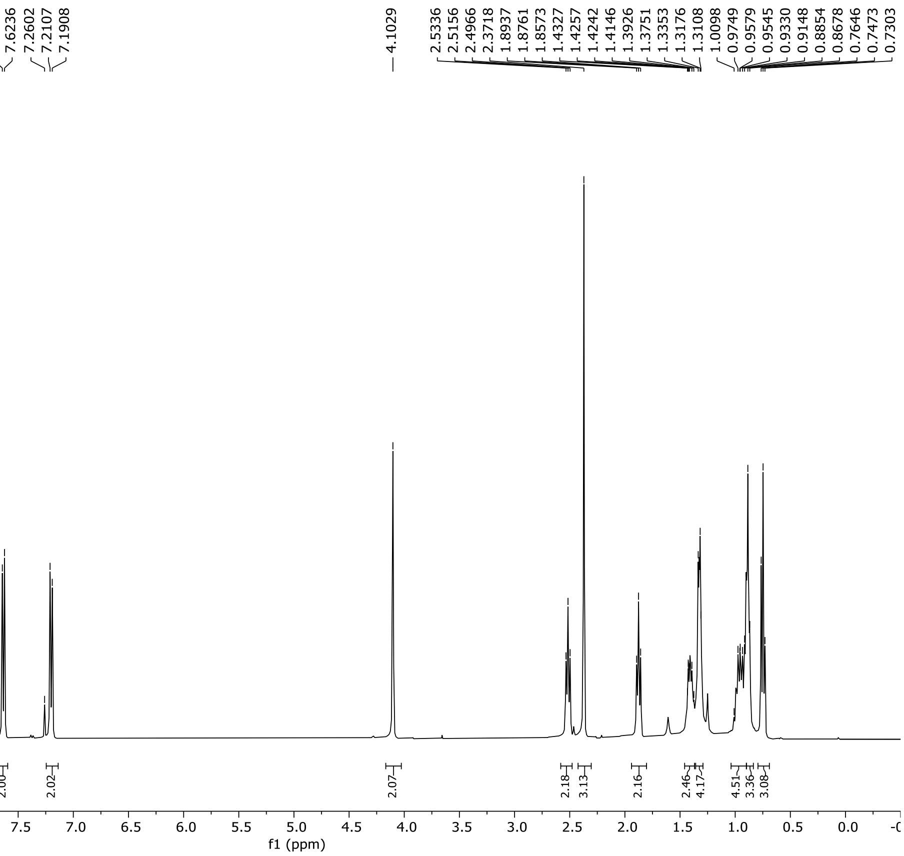

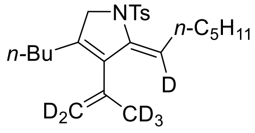

d[6]-**4a**

^{13}C NMR ($CDCl_3$, 100 MHz)

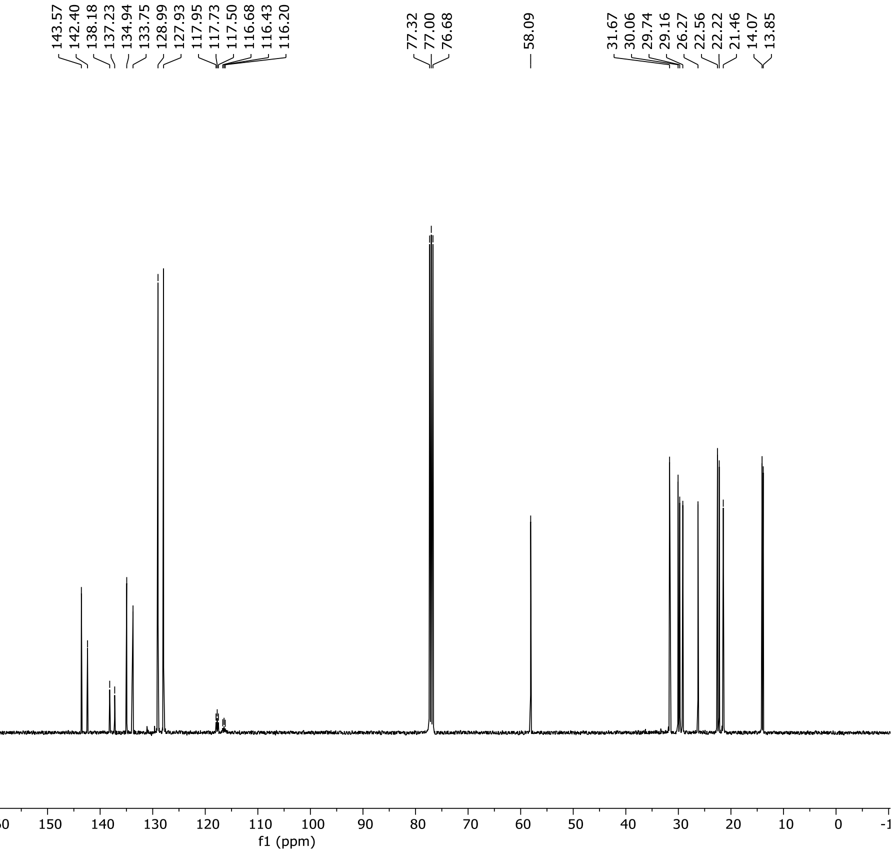

Supplement: Supplementary file 1 — cs1c05147_si_001.pdf [file cs1c05147_si_001.pdf]
